# Joint modeling of longitudinal and competing-risk data using cumulative incidence functions for the failure submodels accounting for potential failure cause misclassification through double sampling

**DOI:** 10.1093/biostatistics/kxac043

**Published:** 2022-11-04

**Authors:** Christos Thomadakis, Loukia Meligkotsidou, Constantin T Yiannoutsos, Giota Touloumi

**Affiliations:** Department of Hygiene, Epidemiology and Medical Statistics, Medical School, National and Kapodistrian University of Athens, Athens, Greece; Department of Mathematics, National and Kapodistrian University of Athens, Athens, Greece; Department of Biostatistics and Health Data Science, Indiana University, 410 West 10th Street, Suite 3000, Indianapolis, IN 46202, USA; Department of Hygiene, Epidemiology and Medical Statistics, Medical School, National and Kapodistrian University of Athens, Athens, Greece

**Keywords:** Bayesian approach, Cumulative incidence function, Joint modeling, Misclassification, Multistate model, Shared parameter models

## Abstract

Most of the literature on joint modeling of longitudinal and competing-risk data is based on cause-specific hazards, although modeling of the cumulative incidence function (CIF) is an easier and more direct approach to evaluate the prognosis of an event. We propose a flexible class of shared parameter models to jointly model a normally distributed marker over time and multiple causes of failure using CIFs for the survival submodels, with CIFs depending on the “true” marker value over time (i.e., removing the measurement error). The generalized odds rate transformation is applied, thus a proportional subdistribution hazards model is a special case. The requirement that the all-cause CIF should be bounded by 1 is formally considered. The proposed models are extended to account for potential failure cause misclassification, where the true failure causes are available in a small random sample of individuals. We also provide a multistate representation of the whole population by defining mutually exclusive states based on the marker values and the competing risks. Based solely on the assumed joint model, we derive fully Bayesian posterior samples for state occupation and transition probabilities. The proposed approach is evaluated in a simulation study and, as an illustration, it is fitted to real data from people with HIV.

## 1. Introduction

A special feature of epidemiological cohort studies is that surrogate markers (e.g., markers related to disease progression) are usually collected over time along with multiple time-to-event outcomes. Such outcomes are often mutually exclusive events, referred to as competing risks (Beyersmann *and others*, 2011). In our motivating example from the epidemiology of human immunodeficiency virus (HIV) infection, patients receiving antiretroviral treatment (ART) can die while in care or disengage from care, which are competing risks; the number of CD4 cells is a longitudinal marker typically collected over time to keep track of HIV progression. If a patient dies or disengages from care (two competing risks), data on CD4 counts are no longer available.

Joint modeling of marker and time-to-event data has been an active research area (e.g., Rizopoulos, 2012; Hickey *and others*, 2018). The aim of joint modeling is 2-fold: to estimate the risk for an event conditional on an endogenous time-updated covariate (Wulfsohn and Tsiatis, 1997) and to adjust for not-at-random missingness (MNAR), as most joint modeling approaches assume that missing marker data after the event are MNAR (Rizopoulos, 2012). However, the distinction between MNAR and missing at random (MAR) marker data is complex and requires further consideration (Thomadakis *and others*, 2019). In this article, we focus on the possibly most frequent case in which a linear mixed model (LMM) is assumed for the marker values, with the “true” marker value (i.e., predicted by both the fixed and the random effects) being included in the survival model. That is, the two processes are linked through common parameters, hence the term shared-parameter models (SPMs) that are frequently used in the literature.

Most of the research in joint modeling assumes that there is a single cause of failure. However, joint modeling of longitudinal data and competing-risk survival data has also gained attention (e.g., Hickey *and others*, 2018; Proust-Lima *and others*, 2016). In principle, competing-risk data can be analyzed through either cause-specific hazards or cumulative incidence functions (CIFs), with the latter being more direct for evaluating the prognosis of a disease. In most cases, though, the competing-risk submodels are specified in terms of the cause-specific hazards under the SPM framework (Elashoff *and others*, 2008; Dantan *and others*, 2011; Rizopoulos, 2012; Andrinopoulou *and others*, 2014), probably due to easier implementation, with the exception of the proportional subdistribution hazard joint model by Deslandes and Chevret (2010). In theory, a cause-specific CIF can be obtained from cause-specific hazards by integrating the product of the respective cause-specific hazard and the overall survival function. However, SPMs require additional integration over the random effects, and the overall survival function is also often approximated by numerical integration (Rizopoulos, 2012). Thus, an SPM in terms of the CIFs (or some function of them) would be more natural and could substantially reduce the computational burden of formally deriving CIF estimates based on cause-specific hazard estimates.

The literature on regression modeling of CIFs has expanded since the seminal paper by Fine and Gray (1999). An issue in such models is that the all-cause CIF should be bounded by 1, which is ignored by some approaches (Fine and Gray, 1999; Jeong and Fine, 2006; Mozumder *and others*, 2018). This can be dealt with by modeling the baseline asymptote for one cause-specific CIF (Shi *and others*, 2013), by adding a small positive number to force the survival function to be positive (Mao and Lin, 2017), or by incorporating a formal boundedness constraint in the maximization process (Bakoyannis *and others*, 2017). However, how to impose such a constraint in SPMs is not so clear as SPMs are defined conditionally on the random effects, and integration over the prior distribution of the random effects is required to obtain the observed data likelihood. Under the Bayesian paradigm, Gelfand *and others* (1992) suggested that when the constraints involve the data (as it is the case in CIF modeling), it is more natural to build the constraints into the likelihood function rather than into the prior distribution.

In HIV studies, especially in those from resource-constrained countries, under-reporting of deaths is often a major issue; patients who have actually died may have been incorrectly classified as disengaged from care, which is failure cause misclassification. To deal with this issue, a double sampling design can be used (Bakoyannis *and others*, 2019); that is, the true vital status of a small random sample drawn from patients reported to have disengaged from care is actively ascertained. This is performed for typically 10–20$\%$ of the patients, thus the true failure cause for the remaining patients is missing. Various methods to adjust for outcome misclassification using double sampling data have been proposed (e.g., Bakoyannis *and others*, 2019; Daniel Paulino *and others*, 2003).

In applied medical research, the progression of cohorts over time is often monitored by using mutually exclusive states defined jointly by competing-risk data and discretized continuous marker data. For example, the United Nations (UN) Joint Programme in HIV/AIDS (UNAIDS) produces various projections for the HIV epidemic using clinically relevant discrete CD4 states (i.e., [0,50), [50,100), [100,200), [200,250), [250,350), [350,500), and [500,] cells/$\mu$L) and time-to-event outcomes through the Spectrum software (Stover *and others*, 2019). This definition of states is clinically meaningful in HIV infection as ART was initiated in the past based on some of the previous CD4 cutoff points. In chronic kidney disease longitudinal studies, glomerular filtration rate (GFR) is a key surrogate of kidney function, which is typically collected over time. Survival states (death and end-stage kidney disease) together with discrete states based on the GFR levels have been used in this context (Hu *and others*, 2012). Hu *and others* (2012) proposed an interesting approach estimating multistate probabilities, but they relied on a two-stage approach without accounting for potential failure cause misclassification.

In this article, we propose a unified and flexible approach to jointly model a continuous marker over time and competing-risk data using CIFs for the failure submodels, accounting for failure cause misclassification using double sampling. Inference is obtained through a Bayesian procedure, and based on the postulated model, we also derive posterior samples for multistate probabilities defined jointly by marker and competing-risk data. In Section 2, we describe the structure of the proposed models, and we describe the extensions required to account for potential failure cause misclassification in Section 3. In Section 4, we derive a procedure to obtain posterior samples for multistate probabilities. In Section 5, a simulation study is performed to evaluate the performance of the proposed methodology, while, in Section 6, we fit the examined models to real data. Finally, in Section 7, we present concluding remarks and discuss limitations and possible extensions.

## 2. Proposed model

### 2.1. Marker model

For the longitudinal marker model, we use an LMM model of the form $ y_{i}(t)=\boldsymbol{x}_{i}^{\top}(t)\boldsymbol{\beta}+\boldsymbol{z}_{i}^{\top}(t)\boldsymbol{b}_{i}+\epsilon_{i}(t)$, where $\boldsymbol{x}_{i}^{\top}(t)$ and $\boldsymbol{z}_{i}^{\top}(t)$ denote the fixed- and random-effects design matrices at time $t$, respectively, and $\boldsymbol{\beta}$ and $\boldsymbol{b}_{i}\sim N(\boldsymbol{0},\boldsymbol{D})$ denote the fixed and random effects, respectively. Furthermore, $\epsilon_{i}(t)\sim N(0,\omega^{-1})$ denotes the within-subject residuals with $\omega$ being the within-subject precision. As usually assumed in the joint modeling literature (Rizopoulos, 2012), the “true” marker value at time $t$ is defined as $m_{i}(t)=\boldsymbol{x}_{i}^{\top}(t)\boldsymbol{\beta}+\boldsymbol{z}_{i}^{\top}(t)\boldsymbol{b}_{i}$ and the history of “true” values up to time $t$ are denoted by $M_{i}(t)=\{m_{i}(s): 0\leq s\leq t\}$. The vector of the observed marker values on the $i$th subject is denoted by $\boldsymbol{Y}_{i}^{\top}=\{y_{i}(t_{i1}),y_{i}(t_{i2}),\ldots,y_{i}(t_{in_{i}})\}$, where $t_{i1},\ldots,t_{in_{i}}$ are the observation times and $n_{i}$ is the number of observed marker values on subject $i$. The corresponding design matrices for the fixed and random effects at times $t_{i1},\ldots, t_{in_{i}}$ are denoted by $\boldsymbol{X}_{i}$ and $\boldsymbol{Z}_{i}$, respectively. The marker model parameters are $\boldsymbol{\theta}_{L}^{\top}=(\boldsymbol{\beta}^{\top},\operatorname{vech}(\boldsymbol{D})^{\top},\omega)$ and $\boldsymbol{b}^{\top}=(\boldsymbol{b}_{1},\ldots,\boldsymbol{b}_{N})$.

### 2.2. Competing-risk survival models

Let $T_{i}^{\star}$ be the time to the first occurring event for the $i$th individual and $K_{i}\in \{1,2,\ldots,K\}$ be the corresponding failure cause. We propose to model the CIFs for all causes simultaneously conditional on the “true” marker values, that is,

(2.1) \begin{equation*}F_{ik}\{t|M_{i}(t),\boldsymbol{w}_{ik};\boldsymbol{\theta}_{sk}\}=\Pr\{T_{i}^{\star}\leq t,K_{i}=k|M_{i}(t),\boldsymbol{w}_{ik};\boldsymbol{\theta}_{sk}\},
\end{equation*}

where $\boldsymbol{w}_{ik}$ is a vector of baseline covariates for cause $k$ and individual $i$ and $\boldsymbol{\theta}_{sk}$ is the parameter vector for cause $k$. Note that (2.1) depends on $\boldsymbol{\beta}$ and $\boldsymbol{b}_{i}$ through the history of the “true” marker values, $M_{i}(t)$. Since all CIFs are modeled simultaneously, the all-cause CIFs should be bounded by 1 at each failure time. To account for that, we assume that all CIFs increase over time up to a common point $\tau_{i}$ at which the all-cause CIF approaches 1 and they reach a plateau thereafter,

(2.2) \begin{equation*}
F_{ik}\{t|M_{i}(t),\boldsymbol{w}_{ik};\boldsymbol{\theta}_{sk}\}
=F_{ik}^{M}\{t|M_{i}(t),\boldsymbol{w}_{ik};\boldsymbol{\theta}_{sk}\}I(0\leq t< \tau_{i}) +F_{ik}^{M}\{\tau_{i}|M_{i}(\tau_{i}),\boldsymbol{w}_{ik};\boldsymbol{\theta}_{sk}\}I(t\geq\tau_{i}),
\end{equation*}

where $F_{ik}^{M}\{t|M_{i}(t),\boldsymbol{w}_{ik};\boldsymbol{\theta}_{sk}\}$ is a certain parametric model for the CIF of cause $k$ conditional on the “true” marker values, $M_{i}(t)$, and some baseline covariates, $\boldsymbol{w}_{ik}$. To be more formal, $\tau_{i}$ depends on the values of $(\boldsymbol{\beta}^{\top},\boldsymbol{\theta}_{s}^{\top},\boldsymbol{b}_{i}^{\top})$, that is, $\tau_{i}\equiv\tau_{i}(\boldsymbol{\beta},\boldsymbol{\theta}_{s},\boldsymbol{b}_{i})\equiv\sup_{t}\left[t:\sum_{k=1}^{K}F_{ik}^{M}\{t|M_{i}(t),\boldsymbol{w}_{ik};\boldsymbol{\theta}_{sk}\}< 1\right]$, where $\boldsymbol{\theta}_{s}=(\boldsymbol{\theta}_{s1}^{\top},\boldsymbol{\theta}_{s2}^{\top},\ldots,\boldsymbol{\theta}_{sK}^{\top})^{\top}$. In other words, the support of the distribution of the survival time $T_{i}^{\star}$ is equal to $(0,\tau_{i})$, with $\tau_{i}=\infty$ if $\sum_{k=1}^{K}F_{ik}^{M}\{t|M_{i}(t),\boldsymbol{w}_{ik};\boldsymbol{\theta}_{sk}\}< 1$ for any $t>0$. The motivation for assuming (2.2) becomes clearer when considering the survival likelihood conditionally on the random effects under noninformative right censoring (Jeong and Fine, 2006); we can only observe $T_{i}=\min(T_{i}^{\star},C_{i})$ and $\delta_{ik}=I(K_{i}=k)$, where $C_{i}$ is the hypothetical censoring time for the $i$th individual, $\delta_{ik}$ is the corresponding failure indicator for cause $k$, $k=1,2,\ldots,K$, and $\delta_{i}=\sum_{k=1}^{K}\delta_{ik}$ is the overall failure indicator. As a convention, we assume that $K_{i}=0$ denotes right censoring. In this case, the survival likelihood is equal to

(2.3) \begin{equation*}
f\{T_{i},K_{i}|M_{i}(T_{i}),\boldsymbol{w}_{i};\boldsymbol{\theta}_{s}\}=
\prod_{k=1}^{K}f_{ik}\{T_{i}|M_{i}(T_{i}),\boldsymbol{w}_{ik};\boldsymbol{\theta}_{sk}\}^{\delta_{ik}}
S_{i}\{T_{i}|M_{i}(T_{i}),\boldsymbol{w}_{i};\boldsymbol{\theta}_{s}\}^{1-\delta_{i}},
\end{equation*}

where $f_{ik}\{x|M_{i}(x),\boldsymbol{w}_{ik};\boldsymbol{\theta}_{sk}\} =I(0< x< \tau_{i})\partial F_{ik}^{M}\{x|M_{i}(x),\boldsymbol{w}_{ik};\boldsymbol{\theta}_{sk}\}/\partial x $ is the density function for cause $k$, $S_{i}\{T_{i}|M_{i}(T_{i}),\boldsymbol{w}_{i};\boldsymbol{\theta}_{s}\}=1-\sum_{k=1}^{K}F_{ik}\{T_{i}|M_{i}(T_{i}),\boldsymbol{w}_{ik};\boldsymbol{\theta}_{sk}\}$ is the overall survival function, and $\boldsymbol{w}_{i}=(\boldsymbol{w}_{i1}^{\top},\boldsymbol{w}_{i2}^{\top},\ldots,\boldsymbol{w}_{iK}^{\top})^{\top}$.

For some specific set of parameter values, $(\boldsymbol{\beta},\boldsymbol{\theta}_{s},\boldsymbol{b}_{i})$, suppose that the assumed model yields an all-cause CIF evaluated at the observed survival time, $T_{i}$, greater than or equal to 1, that is, $\sum_{k=1}^{K}F_{ik}^{M}\{T_{i}|M_{i}(T_{i}),\boldsymbol{w}_{ik};\boldsymbol{\theta}_{sk}\}\geq 1$. By the definition of $\tau_{i}$, it is implied that $T_{i}$ does not lie within the support of $T_{i}^{\star}$ given the parameter values, $(\boldsymbol{\beta},\boldsymbol{\theta}_{s},\boldsymbol{b}_{i})$, that is, $T_{i}\geq\tau_{i}(\boldsymbol{\beta},\boldsymbol{\theta}_{s},\boldsymbol{b}_{i})$. By (2.2), it is further implied that both $f_{ik}\{T_{i}|M_{i}(T_{i}),\boldsymbol{w}_{ik};\boldsymbol{\theta}_{sk}\}$ and $S_{i}\{T_{i}|M_{i}(T_{i}),\boldsymbol{w}_{ik};\boldsymbol{\theta}_{s}\}$ are equal to zero, ensuring that the likelihood function is equal to zero. Thus, (2.3) is equivalent to including the model-based CIF, $F_{ik}^{M}\{T_{i}|M_{i}(T_{i}),\boldsymbol{w}_{ik};\boldsymbol{\theta}_{sk}\}$, and its derivative along with the indicator function $I[\sum_{k=1}^{K}F_{ik}^{M}\{T_{i}|M_{i}(T_{i}),\boldsymbol{w}_{ik};\boldsymbol{\theta}_{sk}\}< 1]$ in (2.3).

We propose to model the CIFs using the class of generalized odds rate transformation models (e.g., Bakoyannis *and others*, 2017; Jeong and Fine, 2006)

\begin{eqnarray*}F_{ik}^{M}\{t|M_{i}(t),\boldsymbol{w}_{ik};\boldsymbol{\theta}_{sk}\}&=& 1 - \exp\left[-\int_{0}^{t} \exp\left\{\boldsymbol{B}_{k}^{\top}(s)\boldsymbol{\psi}_{k}+\boldsymbol{\gamma}_{k}^{\top}\boldsymbol{w}_{ik}+\alpha_{k}m_{i}(s)\right\}{\rm d}s\right],\quad\textbf{SPM-1} \\F_{ik}^{M}\{t|M_{i}(t),\boldsymbol{w}_{ik};\boldsymbol{\theta}_{sk}\}&=& 1 - \left[1+c_{k}\int_{0}^{t} \exp\left\{\boldsymbol{B}_{k}^{\top}(s)\boldsymbol{\psi}_{k}+\boldsymbol{\gamma}_{k}^{\top}\boldsymbol{w}_{ik}+\alpha_{k}m_{i}(s)\right\}{\rm d}s\right]^{-\frac{1}{c_{k}}},\quad\textbf{SPM-2}\end{eqnarray*}

where model SPM-1 is a proportional subdistribution hazard model (Deslandes and Chevret, 2010; Fine and Gray, 1999), since $\lambda_{ik}^{M}\{t|m_{i}(t),\boldsymbol{w}_{ik};\boldsymbol{\theta}_{sk}\}=\exp\left\{\boldsymbol{B}_{k}^{\top}(t)\boldsymbol{\psi}_{k}+\boldsymbol{\gamma}_{k}^{\top}\boldsymbol{w}_{ik}+\alpha_{k}m_{i}(t)\right\}$, where $\lambda_{ik}^{M}\{t|m_{i}(t),\boldsymbol{w}_{ik};\boldsymbol{\theta}_{sk}\}$ is the assumed subdistribution hazard function, whereas SPM-2 reduces to SPM-1 as $c_{k}\rightarrow 0$ (Jeong and Fine, 2006). $\boldsymbol{B}_{k}^{\top}(s)$ denotes a B-splines basis matrix at time $s$, with associated parameter $\boldsymbol{\psi}_{k}$, and $\boldsymbol{\gamma}_{k}$ measures the effect of the baseline covariates on the $k$th CIF. The parameters $\alpha_{k}$, $k=1,\ldots,K$, correspond to the effects of the “true” marker values on the CIFs and indicate the level of association between the marker and the survival submodels (referred to as the association parameters). Under SPM-1, $\exp(\alpha_{k})$ denotes the $k$th subdistribution hazard ratio at time $t$, resulting from one unit increase in $m_{i}(t)$ at the same time point. The interpretation of the parameters of SPM-2 does not seem so appealing in general, but assuming $c_{k}=1$ a proportional rate of odds increase model is implied, that is, $\partial(F_{ik}^{M}\{t|M_{i}(t),\boldsymbol{w}_{ik};\boldsymbol{\theta}_{sk}\} /[1-F_{ik}^{M}\{t|M_{i}(t),\boldsymbol{w}_{ik};\boldsymbol{\theta}_{sk}\} ])/\partial t=\exp\left\{\boldsymbol{B}_{k}^{\top}(t)\boldsymbol{\psi}_{k}+\boldsymbol{\gamma}_{k}^{\top}\boldsymbol{w}_{ik}+\alpha_{k}m_{i}(t)\right\}$. Thus, SPM-2 would be useful when the SPM-1 model does not provide good fit to the data. We assume that the parameters $c_{k}$ are known, as trying to estimate them can lead to nonidentifiability issues (Bakoyannis *and others*, 2017). When this is not true, a feasible, although not optimal approach to select the link function parameters is to perform a grid search over plausible values of $(c_{1},c_{2},\ldots,c_{K})$ and select the one that optimizes some model comparison criterion.

### 2.3. Bayesian inferential procedures

The observed data likelihood of the model requires multidimensional integration over the random effects. Since such integration is challenging, especially given the constrained space due to (2.2), we rely on a Bayesian inferential procedure based on a Markov chain Monte Carlo (MCMC) algorithm. Letting $\boldsymbol{\theta}=(\boldsymbol{\theta}_{L}^{\top},\boldsymbol{\theta}_{s}^{\top})^{\top}$ be the entire parameter vector of the models and $\mathcal{D}=\left\{ (\boldsymbol{Y}_{i},\boldsymbol{X}_{i},\boldsymbol{Z}_{i},T_{i},K_{i},\boldsymbol{w}_{i}), i=1,2,\ldots,N \right\}$ be the observed data, the posterior distribution of all unknown quantities is proportional to $f(\boldsymbol{\theta}) \prod_{i=1}^{N}\left[\,f(\boldsymbol{Y}_{i}|\boldsymbol{b}_{i};\boldsymbol{\theta}_{L})f(\boldsymbol{b}_{i};\boldsymbol{\theta}_{L})f\{T_{i},K_{i}|M_{i}(T_{i}),\boldsymbol{w}_{i};\boldsymbol{\theta}_{s}\}\right]$, where $f(\boldsymbol{\theta})$ is the prior distribution of the parameters. The integrals involved in the CIFs can be accurately approximated by Gauss–Legendre rules with 30 nodes. A Normal prior distribution, $N(\boldsymbol{\mu}_{0},\boldsymbol{C}_{0})$, is used for $\boldsymbol{\beta}$, a $\operatorname{Gamma}(\lambda_{1},\lambda_{2})$ for $\omega$ and a Normal, $N(\boldsymbol{\mu}_{0}^{s},\boldsymbol{C}_{0}^{s})$, distribution for $\boldsymbol{\theta}_{s}$. For the covariance matrix of the random effects, $\boldsymbol{D}$, we assumed the Inverse-Wishart $IW(\boldsymbol{A},df)$ distribution. To update the parameter values, we used Gibbs steps wherever possible and Metropolis–Hastings steps for the remaining parameters. Further details on the MCMC algorithm are presented in Section S1 of the Supplementary material available at *Biostatistics* online. It needs to be emphasized that any proposed value leading to an all-cause model-based CIF greater than or equal to 1, that is, $\sum_{k=1}^{K}F_{ik}^{M}\{t|M_{i}(t),\boldsymbol{w}_{ik};\boldsymbol{\theta}_{sk}\}\geq 1$, is rejected as the posterior ratio is equal to zero. Thus, calculation of $\tau_{i}(\boldsymbol{\beta},\boldsymbol{\theta}_{s},\boldsymbol{b}_{i})$ is not required within the MCMC algorithm.

## 3. Inference under potentially misclassified causes of failure

When the true failure cause, $K_{i}$, is not observed for all individuals, we assume that a cause of failure, $\tilde{K}_{i}$, is always reported although potentially misclassified (i.e., $K_{i}\neq\tilde{K}_{i}$). Let $\pi_{jk}(\mathcal{D}_{\mbox{misc},i})=\Pr(\tilde{K}_{i}=j|K_{i}=k,\mathcal{D}_{\mbox{misc},i};\boldsymbol{\theta}_{\rm misc})$ be the probability of observing failure cause $j$ given the $k$th true failure cause and the history of the observed information up to $T_{i}$ (including but not limited to the observed marker values), $\mathcal{D}_{\mbox{misc},i}$, with $\boldsymbol{\theta}_{\rm misc}$ being the associated parameter vector. Note also that $\pi_{kk}(\mathcal{D}_{\mbox{misc},i})$ is the probability of correctly classifying cause $k$, whereas $\sum_{j=1}^{K}\pi_{jk}(\mathcal{D}_{\mbox{misc},i})=1$, for any $k\in\{1,2,\ldots,K\}$. Moreover, we assume that (i) noninformative right censoring (e.g., administrative censoring) is correctly classified, that is, $K_{i}=0\Leftrightarrow\tilde{K}_{i}=0$ and (ii) the true failure cause is known in a small random sample of individuals, leading to a double sampling design (e.g., Bakoyannis *and others*, 2019). In this context, the observed data are

(3.4) \begin{equation*}
\mathcal{D}_{\mbox{obs}}=
\begin{cases}
(\boldsymbol{Y}_{i},\boldsymbol{X}_{i},\boldsymbol{Z}_{i},T_{i},K_{i},\tilde{K}_{i},\boldsymbol{w}_{i},\mathcal{D}_{\mbox{misc},i},R_{i}) \ \text{if} \ R_{i}=1, \ i=1,\ldots,N, \\
(\boldsymbol{Y}_{i},\boldsymbol{X}_{i},\boldsymbol{Z}_{i},T_{i},\tilde{K}_{i},\boldsymbol{w}_{i},\mathcal{D}_{\mbox{misc},i},R_{i}) \ \text{if} \ R_{i}=0, \ i=1,\ldots,N.
\end{cases},
\end{equation*}

where $R_{i}$ is an indicator function of the $i$th individual being doubly sampled. We make the MAR assumption for the missing failure causes, that is, the probability of being in the double sample depends on the observed data, but not on the missing true failure cause and the random effects. This implies that the true failure cause can be validly predicted based on the observed data. As shown in Section S2 of the Supplementary material available at *Biostatistics* online, the true failure cause probabilities conditionally on the observed data, $\Pr\{K_{i}=k|\tilde{K}_{i}=j,T_{i}^{\star}=t,M_{i}(t),\boldsymbol{w}_{i},\mathcal{D}_{\mbox{misc},i};\boldsymbol{\theta},\boldsymbol{\theta}_{\rm misc}\}$, is equal to

(3.5) \begin{equation*}
f_{ik}\{t|M_{i}(t),\boldsymbol{w}_{ik};\boldsymbol{\theta}_{sk}\}\pi_{jk}(\mathcal{D}_{\mbox{misc},i})\Big/\left[\sum_{k=1}^{K}f_{ik}\{t|M_{i}(t),\boldsymbol{w}_{ik};\boldsymbol{\theta}_{sk}\}\pi_{jk}(\mathcal{D}_{\mbox{misc},i})\right].
\end{equation*}

For individuals that are not doubly sampled, we observe only $\tilde{K}_{i}$, which may be different from $K_{i}$. To deal with this issue, one can use data augmentation (Tanner and Wong, 1987), augmenting the observed likelihood for individuals who have failed from any event but have not been included in the double sampling by the unobserved true failure causes, $K_{i}$. Letting $\mathcal{I}_{\rm mis}$ be the indices of individuals that have failed from any event but are not doubly sampled ($\mathcal{I}_{\rm mis}\equiv\{i: \tilde{K}_{i}\neq 0 \ \&~ R_{i}=0\}$), the augmented posterior distribution of all unknown quantities is equal to

\begin{eqnarray*}
f(\boldsymbol{\theta},\boldsymbol{b},\boldsymbol{\theta}_{\rm misc},\{K_{i}: i\in \mathcal{I}_{\rm mis}\}|\mathcal{D}_{\mbox{obs}})\propto
f(\boldsymbol{\theta})f(\boldsymbol{\theta}_{\rm misc})
\prod_{i=1}^{N}
\Bigg[
f(\boldsymbol{Y}_{i}|\boldsymbol{b}_{i};\boldsymbol{\theta}_{L})f(\boldsymbol{b}_{i};\boldsymbol{\theta}_{L}) \nonumber\\
\prod_{k=1}^{K}f_{ik}\{T_{i}|M_{i}(T_{i}),\boldsymbol{w}_{ik};\boldsymbol{\theta}_{sk}\}^{\delta_{ik}}
S_{i}\{T_{i}|M_{i}(T_{i}),\boldsymbol{w}_{i};\boldsymbol{\theta}_{s}\}^{1-\delta_{i}}\prod_{j=1}^{K}\prod_{k=1}^{K}\pi_{jk}(\mathcal{D}_{\mbox{misc},i})^{\tilde{\delta}_{ij}\delta_{ik}}
\Bigg], \nonumber
\end{eqnarray*}

where the full survival likelihood, $f\{T_{i},K_{i},\tilde{K}_{i}|M_{i}(T_{i}),\boldsymbol{w}_{i},\mathcal{D}_{\mbox{misc},i};\boldsymbol{\theta}_{s},\boldsymbol{\theta}_{\rm misc}\}$, has been factorized as the product of $f\{T_{i},K_{i}|M_{i}(T_{i}),\boldsymbol{w}_{i};\boldsymbol{\theta}_{s}\}$ and $\Pr(\tilde{K}_{i}|K_{i},\mathcal{D}_{\mbox{misc},i};\boldsymbol{\theta}_{\rm misc})$, with $\tilde{\delta}_{ij}=I(\tilde{K}_{i}=j)$. Note also that $\Pr(\tilde{K}_{i}=0|K_{i}=0,\mathcal{D}_{\mbox{misc},i};\boldsymbol{\theta}_{\rm misc})=1$, for the right censored individuals. The following algorithm outlines the modified MCMC procedure to account for misclassification: choosing appropriate initial values $\boldsymbol{\theta}^{(0)},\boldsymbol{b}^{(0)},\{K_{i}^{(0)}: i\in \mathcal{I}_{mis}\},\boldsymbol{\theta}_{\rm misc}^{(0)}$, meeting the likelihood constraints for all individuals, for $l=1,2,\ldots,L$:

- Update $(\boldsymbol{\theta}^{(l-1)},\boldsymbol{b}^{(l-1)})$ to $(\boldsymbol{\theta}^{(l)},\boldsymbol{b}^{(l)})$ according to the posterior distribution $f(\boldsymbol{\theta},\boldsymbol{b}|\{K_{i}^{(l-1)}: i\in \mathcal{I}_{\rm mis}\},\{K_{i}: i \notin \mathcal{I}_{\rm mis}\}, \{(\boldsymbol{Y}_{i},\boldsymbol{X}_{i},\boldsymbol{Z}_{i},T_{i},\boldsymbol{w}_{i}), i=1,\ldots,N\})$, that is, the posterior distribution of the parameters of main interest, with the missing failure causes being equal to their current values. The MCMC algorithm for fully observed failure causes can be used.
- Update $\boldsymbol{\theta}_{\rm misc}^{(l-1)}$ to $\boldsymbol{\theta}_{\rm misc}^{(l)}$ according to $f(\boldsymbol{\theta}_{\rm misc}|\{K_{i}^{(l-1)}: i\in \mathcal{I}_{\rm mis}\},\{K_{i}: i \notin \mathcal{I}_{\rm mis}\},\{\mathcal{D}_{\mbox{misc},i}\}_{i=1}^{N})$.
- Sample $\{K_{i}^{(l)}: i\in \mathcal{I}_{\rm mis}\}$ directly using Equation (3.5).

Thus, data augmentation results in a simple MCMC scheme as, conditionally on the true failure causes, $K_{i}$, the posterior distribution of $(\boldsymbol{\theta},\boldsymbol{b})$ is independent of $\boldsymbol{\theta}_{\rm misc}$ and it has the same form as with the case of fully observed failure causes. The simplicity, though, of data augmentation comes at a price as repeatedly sampling $K_{i}$ may slowly converge towards its limit distribution.

Although the misclassification probabilities do not directly depend on the random effects, they are conditional on the true failure cause, with the missing failure causes imputed based on the CIFs, which in turn depend on the random effects. The simplest approach is to not include any covariate information in $\pi_{jk}(\mathcal{D}_{\mbox{misc},i})$, that is, $\mathcal{D}_{\mbox{misc},i}$ assumed to be an empty set. Then, a natural choice for the prior distributions of the $\pi_{jk}$’s would be the Dirichlet (Beta for $K=2$) distributions, leading to conditional conjugacy. More generally, to model $\pi_{jk}(\mathcal{D}_{\mbox{misc},i})$ conditional on the observed information (i.e., $\mathcal{D}_{\mbox{misc},i}$), multinomial logistic regression could be used. More information is provided in Section S3 of the Supplementary material available at *Biostatistics* online.

To compare the fit of the models, we adopted the marginalized version (Quintero and Lesaffre, 2018) of the deviance information criterion (DIC), which requires integration of the random effects. Integration was performed through Monte Carlo integration or importance sampling when the former fails (Section S4 of the Supplementary material available at *Biostatistics* online).

## 4. Posterior inferences for population-averaged CIFs and marker states

To describe the cohort evolution over time, states defined by marker data and clinical outcomes are often used. A pragmatic approach to do so is to discretize the marker values into nonoverlapping intervals $\{[s_{0},s_{1}),\ldots, [s_{J-1},s_{J})\}$ and define mutually exclusive states based on clinical events and (discretized) marker data. If the focus of the analysis lies in describing the “true” biological process, as often is the case in the joint modeling literature, states may be defined in terms of the “true” marker values, that is, for any $t>0$, $\{m_{i}(t)\in S_{h},T_{i}^{\star}>t\}$, $h=1,\ldots,J$ and $\{T_{i}^{\star}\leq t,K_{i}=k\}$, $k=1,\ldots,K$, where $S_{h}=[s_{h-1},s_{h})$. Progression of the whole cohort can be easily monitored by a series of estimated multistate probabilities $\Pr\{m_{i}(t)\in S_{h},T_{i}^{\star}>t|\boldsymbol{w}_{i};\boldsymbol{\theta}\}$, $h=1,\ldots,J$ and $\Pr(T_{i}^{\star}\leq t,K_{i}=k|\boldsymbol{w}_{ik};\boldsymbol{\theta})$, $k=1,\ldots,K$, through a multistate probability plot. The first quantity, referred to as latent marker state probability, expresses the probability of being event free and having true marker values in $S_{h}$. The second expression, that is, $\Pr(T_{i}^{\star}\leq t,K_{i}=k|\boldsymbol{w}_{ik};\boldsymbol{\theta})$, is the population-averaged CIF for a particular cause. To get better insight into the dynamics of the processes, one may be also interested in transitions between states. In real-life applications (e.g., Stover *and others*, 2019), for simplicity, transitions are often defined from baseline states. Letting $p_{g}(0)=\Pr\{m_{i}(0)\in S_{g};\boldsymbol{\theta}_{L}\}$, it can be easily shown that

(4.6) \begin{align*}
&\Pr\{T_{i}^{\star}\leq t,K_{i}=k|m_{i}(0)\in S_{g},\boldsymbol{w}_{ik};\boldsymbol{\theta}\}=\int\limits_{{m_{i}(0)\in S_{g}}}F_{ik}\{t|M_{i}(t),\boldsymbol{w}_{ik};\boldsymbol{\theta}_{sk}\}
\frac{f(\boldsymbol{b}_{i};\boldsymbol{\theta}_{L})}{p_{g}(0)}{\rm d}\boldsymbol{b}_{i}
\end{align*}

(4.7) \begin{align*}
&\Pr\{m_{i}(t)\in S_{h},T_{i}^{\star}>t|m_{i}(0)\in S_{g},\boldsymbol{w}_{i};\boldsymbol{\theta}\}
=\int\limits_{{m_{i}(0)\in S_{g}, m_{i}(t)\in S_{h}}}S_{i}\{t|M_{i}(t),\boldsymbol{w}_{i};\boldsymbol{\theta}_{s}\}
\frac{f(\boldsymbol{b}_{i};\boldsymbol{\theta}_{L})}{p_{g}(0)}{\rm d}\boldsymbol{b}_{i}.
\end{align*}

Inference on (4.6) and (4.7) involves two distinct problems (i) approximation of the integral over the random effects and (ii) accounting for the variability in $\boldsymbol{\theta}$.

### 4.1. Estimation procedure

We initially describe the estimation of (4.6) and (4.7) for any given $\boldsymbol{\theta}$. Specifically, (4.6) can be approximated by drawing samples $\{\boldsymbol{b}_{ig}^{(j)}\}_{j=1}^{N_{\mbox{mc}}}$ for $\boldsymbol{b}_{i}$ from the $N(\boldsymbol{0},\boldsymbol{D})$ distribution under the linear constraint $m_{i}(0)\in S_{g}$, which can be carried out, among many other options (e.g., Gibbs sampling), through Hamiltonian Monte Carlo (Pakman, 2015). Specifically,

(4.8) \begin{eqnarray*}
\Pr\{T_{i}^{\star}\leq t,K_{i}=k|m_{i}(0)\in S_{g},\boldsymbol{w}_{ik};\boldsymbol{\theta}\}\approx
N_{\mbox{mc}}^{-1}\sum_{j=1}^{N_{\mbox{mc}}}F_{ik}\{t|M_{ig}^{(j)}(t),\boldsymbol{w}_{ik};\boldsymbol{\theta}_{sk}\},
\end{eqnarray*}

where $m_{ig}^{(j)}(t)=\boldsymbol{x}_{i}^{\top}(t)\boldsymbol{\beta}+\boldsymbol{z}_{i}^{\top}(t)\boldsymbol{b}_{ig}^{(j)}$ and $M_{ig}^{(j)}(t)=\{m_{ig}^{(j)}(s): 0\leq s\leq t\}$. Similarly, it follows that, after multiplying and dividing (4.7) by $\Pr\{ m_{i}(t)\in S_{h},m_{i}(0)\in S_{g};\boldsymbol{\theta}\}$, (4.7) can be approximated using samples $\{\boldsymbol{b}_{igh}^{(j)}\}_{j=1}^{N_{\mbox{mc}}}$ from the $N(\boldsymbol{0},\boldsymbol{D})$ distribution under the linear constraints $m_{i}(0)\in S_{g}$ and $m_{i}(t)\in S_{h}$, that is, $\Pr\{m_{i}(t)\in S_{h},T_{i}^{\star}>t|m_{i}(0)\in S_{g},\boldsymbol{w}_{i};\boldsymbol{\theta}\}$ can be approximated by

(4.9) \begin{eqnarray*}
\frac{\Pr\{ m_{i}(t)\in S_{h},m_{i}(0)\in S_{g};\boldsymbol{\theta}\}}{\Pr\{ m_{i}(0)\in S_{g};\boldsymbol{\theta}\}N_{\mbox{mc}}}
\sum_{j=1}^{N_{\mbox{mc}}}S_{i}\{t|M_{igh}^{(j)}(t),\boldsymbol{w}_{i};\boldsymbol{\theta}_{s}\},
\end{eqnarray*}

where $m_{igh}^{(j)}(t)=\boldsymbol{x}_{i}^{\top}(t)\boldsymbol{\beta}+\boldsymbol{z}_{i}^{\top}(t)\boldsymbol{b}_{igh}^{(j)}$ and $M_{igh}^{(j)}(t)=\{m_{igh}^{(j)}(s): 0\leq s\leq t\}$. Since $m_{i}(t)\sim N\{\boldsymbol{x}_{i}^{\top}(t)\boldsymbol{\beta},\boldsymbol{z}_{i}^{\top}(t)\boldsymbol{D}\boldsymbol{z}_{i}(t)\}$, $\Pr\{ m_{i}(t)\in S_{h},m_{i}(0)\in S_{g};\boldsymbol{\theta}\}$ and $\Pr\{ m_{i}(0)\in S_{g};\boldsymbol{\theta}\}$ can be easily computed using the cumulative distribution function of the (bivariate) Normal distribution. Due to (2.2), it should be noted that if $\sum_{k=1}^{K}F_{ik}^{M}\{t|M_{ig}^{(j)}(t),\boldsymbol{w}_{ik};\boldsymbol{\theta}_{sk}\}>1$, $F_{ik}\{t|M_{ig}^{(j)}(t),\boldsymbol{w}_{ik};\boldsymbol{\theta}_{sk}\}=F_{ik}\{t^{\prime}|M_{ig}^{(j)}(t^{\prime}),\boldsymbol{w}_{ik};\boldsymbol{\theta}_{sk}\}$, where $t^{\prime}=\tau_{i}(\boldsymbol{\beta},\boldsymbol{\theta}_{s},\boldsymbol{b}_{ig}^{(j)})$, thus calculation of the upper bound is required only for the random draws that do not fulfill the boundedness constraint.

A posterior sample for (4.6) and (4.7) can be obtained by (a) drawing $\boldsymbol{\theta}^{(l)}\sim f(\boldsymbol{\theta}|\mathcal{D}_{\mbox{obs}})$, $l=1,2,\ldots,L$ and (b) approximating $\Pr\{T_{i}^{\star}\leq t,K_{i}=k|m_{i}(0)\in S_{g},\boldsymbol{w}_{ik};\boldsymbol{\theta}^{(l)}\}$ and $\Pr\{m_{i}(t)\in S_{h},T_{i}^{\star}>t|m_{i}(0)\in S_{g},\boldsymbol{w}_{i};\boldsymbol{\theta}^{(l)}\}$, for each $l=1,2,\ldots,L$, using (4.8) and (4.9). Thus, posterior means and posterior credible intervals can easily be estimated. Also, once posterior samples for (4.6) and (4.7) are available, it is easy to get posterior samples for population-averaged CIFs and latent marker state probabilities through the following relationships $\Pr(T_{i}^{\star}\leq t,K_{i} = k|\boldsymbol{w}_{ik};\boldsymbol{\theta})=\sum_{g=1}^{J}\Pr\{T_{i}^{\star}\leq t,K_{i}=k|m_{i}(0)\in S_{g},\boldsymbol{w}_{ik};\boldsymbol{\theta}\}\Pr\{m_{i}(0)\in S_{g};\boldsymbol{\theta}\}$ and $\Pr\{m_{i}(t)\in S_{h},T_{i}^{\star}>t|\boldsymbol{w}_{i};\boldsymbol{\theta}\}=\sum_{g=1}^{J}\Pr\{m_{i}(t)\in S_{h},T_{i}^{\star}>t|m_{i}(0)\in S_{g},\boldsymbol{w}_{i};\boldsymbol{\theta}\}\Pr\{m_{i}(0)\in S_{g};\boldsymbol{\theta}\}$, respectively.

In theory, $\sum_{k=1}^{K}\Pr\{T_{i}^{\star}\leq t,K_{i}=k|m_{i}(0)\in S_{g},\boldsymbol{w}_{ik};\boldsymbol{\theta}\}$ is equal to $1-\sum_{h=1}^{J}\Pr\{m_{i}(t)\in S_{h},T_{i}^{\star}>t|m_{i}(0)\in S_{g},\boldsymbol{w}_{i};\boldsymbol{\theta}\}$. However, due to Monte Carlo approximation error, results using (4.6) and (4.7) might differ slightly. To get consistent results, we used $1-\sum_{k=2}^{K}\Pr\{T_{i}^{\star}\leq t,K_{i}=k|m_{i}(0)\in S_{g},\boldsymbol{w}_{ik};\boldsymbol{\theta}^{(l)}\}-\sum_{h=2}^{J}\Pr\{m_{i}(t)\in S_{h},T_{i}^{\star}>t|m_{i}(0)\in S_{g},\boldsymbol{w}_{i};\boldsymbol{\theta}^{(l)}\}$ as the posterior sample for $\Pr\{T_{i}^{\star}\leq t,K_{i}=1|m_{i}(0)\in S_{g},\boldsymbol{w}_{i1};\boldsymbol{\theta}\}$, $g=1,\ldots,J$.

### 4.2. CIF estimates conditional on observed marker states

In a clinical application, estimating the population-averaged CIF conditional on the observed marker state could be valuable for making projections about the future cohort evolution. Thus, CIFs given observed baseline state, $\Pr\{T_{i}^{\star}\leq t,K_{i}=k|y_{i}(0)\in S_{g},\boldsymbol{w}_{ik};\boldsymbol{\theta}\}$, $g=1,\ldots,J$, could be of interest. Similarly, one may be also interested in CIFs conditional on being in certain observed states at specific time points. In this case, it would be reasonable to also condition on survival up to the last time point and the baseline state, that is, $\Pr\{T_{i}^{\star}\leq t,K_{i}=k|T_{i}^{\star}>s,y_{i}(0)\in S_{g},y_{i}(s)\in S_{h},\boldsymbol{w}_{i};\boldsymbol{\theta}\}$, for $0\leq s < t$ and $g,h \in\{1,2,\ldots,J\}$. Such estimates could be useful for identifying certain subsets of the population who are event free and at high risk for developing any of the events. Estimation of these probabilities is outlined in Section S5 of the Supplementary material available at *Biostatistics* online.

## 5. Simulation studies

A simulation study was carried out to evaluate the performance of the proposed methodology under certain conditions. Marker data were generated using a piece-wise linear LMM, $ y_{i}(t)=(\beta_{0}+b_{i0}) + (\beta_{1}+b_{i1})\min(t,1) + (\beta_{2}+b_{i2})[\max\{\min(t,5),1\}-1] + \beta_{3}\{\max(t,5)-5\} +\epsilon_{i}(t)$, with $(b_{i0},b_{i1},b_{i2})\sim N(\boldsymbol{0},\boldsymbol{D})$ and $\epsilon_{i}(t)\sim N(0,\omega^{-1})$. Thus, the population slopes are $\beta_{1}$, $\beta_{2}$, and $\beta_{3}$ when $t\in[0,1)$, $t\in[1,5)$, and $t>5$, respectively. Measurements were assumed to be collected biannually and the maximum study duration was assumed to be 10 years. We assumed two competing risks with $K_{i}=1,2$ corresponding to death and disengagement from care, respectively. Two scenarios regarding the competing-risk data were considered: survival data were simulated based on both the SPM-1 model and the SPM-2 model with $c_{1}=c_{2}=1$, with respective equations $F_{ik}^{M}\{t|M_{i}(t),w_{i};\boldsymbol{\theta}_{sk}\} = 1 - \exp\left[-\int_{0}^{t} u_{k1}(s)\exp\left\{\gamma_{k}w_{i}+\alpha_{k}m_{i}(s)\right\}{\rm d}s\right]$ and $F_{ik}^{M}\{t|M_{i}(t),w_{i};\boldsymbol{\theta}_{sk}\} = 1 - \left[1+\int_{0}^{t} u_{k2}(s)\exp\left\{\gamma_{k}w_{i}+\alpha_{k}m_{i}(s)\right\}{\rm d}s\right]^{-1}$, where $k=1,2$ and $w_{i}$ is a binary baseline covariate following the Bernoulli distribution with probability 0.5. An independent right censoring mechanism was also applied using $C_{i}\sim \min(U_{i},10)$, where $U_{i}\sim \operatorname{Exp}(0.025)$ (i.e., the exponential distribution with rate = 0.025), leading to around 50$\%$ censoring rate. The reported failure cause was generated conditional on the first and the second true failure cause with probabilities $\pi_{11}=0.75$ and $\pi_{22}=0.90$, respectively, whereas 15$\%$ of the noncensored observations was included in the double sample. For each simulation scenario, we simulated 500 data sets including $N=2000$ individuals per data set.

Under each of the two scenarios for the survival submodels, we fitted the proposed model using both the SPM-1 and SPM-2 parameterizations, with the marker model being correctly specified in the fitted models. We also examined the performance of the proposed approach in deriving inferences on the quantities described in Section 4. These estimates were produced at times 0, 2, 4, 6, 8, and 10 years. For each fitted model, we calculated the marginal DIC criterion. Its performance in correctly identifying the true model was assessed by recording the proportion of time the true model was chosen under both scenarios. Further details on the simulation study are provided in Section S5 of the Supplementary material available at *Biostatistics* online.

To assess model performance, we present the bias, the Monte Carlo standard deviation, the average model-based standard error, and the empirical coverage probability of credible intervals. Since parameters $\gamma_{k}$ and $\alpha_{k}$ do not have the same interpretation under SPM-1 and SPM-2, we did not provide bias and coverage probability results for $\gamma_{k}$ and $\alpha_{k}$ when the fitted model was misspecified. The results under the SPM-1 and SPM-2 scenarios are presented in Tables 1 and 2, respectively. The fixed-effect estimates were approximately unbiased for both models under the two scenarios (bias from $-$0.010 to 0.007), while the coverage probabilities were close to the nominal level (from 93.2$\%$ to 95.8$\%$). The estimates of $\gamma_{k}$ and $\alpha_{k}$ were nearly unbiased along with approximately 95$\%$ coverage probabilities when the fitted model coincided with the true data generating mechanism. For the misclassification parameters (i.e., $\pi_{11}$ and $\pi_{22}$), the bias was small, with decent coverage rates. The DIC criterion had a moderate ability to identify the correct model as it selected the true model 75.0$\%$ and 58.2$\%$ of the time under the SPM-1 and SPM-2 scenarios, respectively. Its ability to correctly identify the true model substantially increased to 88.0$\%$ and 82.0$\%$, respectively, when a simulation study including 8000 individuals with 50 replications was performed (further results not shown). Focusing on the population CIF estimates, both models yielded estimates with negligible bias along with adequate empirical coverage probabilities, while the Monte Carlo standard deviation was close to the average model-based standard error. Results for the remaining quantities presented in Section 4 were roughly similar and are presented in detail in Section S7 of the Supplementary material available at *Biostatistics* online.

**Table 1:** Simulation study results from fitted SPM-1 and SPM-2 models when the data are generated by the SPM-1 model under failure cause misclassification

Parameter	True	Median	Bias	ASD	MCSD	Coverage	Median	Bias	ASD	MCSD	Coverage
Longitudinal	Results from SPM-1						Results from SPM-2				
Intercept	12.850	12.857	0.007	0.126	0.122	94.6	12.856	0.006	0.126	0.122	94.4
Slope1 ($\beta_1$)	6.030	6.027	$-$ 0.003	0.109	0.104	95.8	6.020	$-$ 0.010	0.109	0.105	95.8
Slope2 ($\beta_2$)	0.770	0.769	$-$ 0.001	0.031	0.030	95.4	0.767	$-$ 0.003	0.031	0.030	95.2
Slope3 ($\beta_3$)	0.000	$-$ 0.001	$-$ 0.001	0.017	0.017	94.2	$-$ 0.001	$-$ 0.001	0.017	0.017	93.8
Cause1 (e.g. death)											
“True” marker value ($\alpha_{1}$)	$-$ 0.160	$-$ 0.162	$-$ 0.002	0.017	0.018	94.2	$-$ 0.183		0.020	0.021	
Binary covariate ($\gamma_{1}$)	0.150	0.148	$-$ 0.002	0.151	0.152	94.2	0.163		0.172	0.172	
CIF1 $t$ = 2, $w=1$	8.175	8.136	$-$ 0.040	1.049	1.047	94.6	8.111	$-$ 0.065	1.058	1.060	94.8
CIF1 $t$ = 4, $w=1$	11.533	11.350	$-$ 0.183	1.380	1.384	94.2	11.322	$-$ 0.211	1.384	1.391	94.8
CIF1 $t$ = 6, $w=1$	13.495	13.248	$-$ 0.248	1.588	1.602	94.2	13.207	$-$ 0.288	1.586	1.593	95.0
CIF1 $t$ = 8, $w=1$	14.774	14.474	$-$ 0.301	1.729	1.751	94.4	14.426	$-$ 0.348	1.721	1.732	95.6
CIF1 $t$ = 10, $w=1$	15.604	15.219	$-$ 0.384	1.824	1.847	93.8	15.161	$-$ 0.442	1.811	1.822	95.0
CIF1 $t$ = 2, $w=0$	7.106	7.093	$-$ 0.013	0.989	1.009	93.6	7.041	$-$ 0.066	0.989	1.005	93.8
CIF1 $t$ = 4, $w=0$	10.062	9.934	$-$ 0.127	1.319	1.354	94.8	9.895	$-$ 0.166	1.311	1.344	94.4
CIF1 $t$ = 6, $w=0$	11.799	11.617	$-$ 0.181	1.527	1.569	93.8	11.581	$-$ 0.217	1.511	1.550	93.6
CIF1 $t$ = 8, $w=0$	12.935	12.710	$-$ 0.226	1.667	1.722	93.4	12.679	$-$ 0.256	1.647	1.689	93.2
CIF1 $t$ = 10, $w=0$	13.673	13.375	$-$ 0.299	1.761	1.819	93.0	13.345	$-$ 0.328	1.738	1.781	93.0
Cause2 (e.g. disengagement)											
“True” marker value ($\alpha_{2}$)	$-$ 0.020	$-$ 0.021	$-$ 0.001	0.010	0.010	93.6	$-$ 0.026		0.012	0.012	
Binary covariate ($\gamma_{2}$)	$-$ 0.150	$-$ 0.152	$-$ 0.002	0.089	0.088	95.0	$-$ 0.185		0.110	0.108	
CIF2 $t$ = 2, $w=1$	11.525	11.487	$-$ 0.037	1.058	1.093	94.4	11.449	$-$ 0.075	1.090	1.120	95.0
CIF2 $t$ = 4, $w=1$	20.352	20.322	$-$ 0.030	1.519	1.581	93.4	20.326	$-$ 0.026	1.560	1.606	94.2
CIF2 $t$ = 6, $w=1$	27.328	27.297	$-$ 0.031	1.823	1.884	93.2	27.380	0.052	1.849	1.889	94.0
CIF2 $t$ = 8, $w=1$	32.937	32.963	0.027	2.042	2.092	93.0	33.152	0.216	2.039	2.074	94.0
CIF2 $t$ = 10, $w=1$	37.431	37.538	0.107	2.204	2.255	92.6	37.818	0.387	2.173	2.199	93.2
CIF2 $t$ = 2, $w=0$	13.260	13.239	$-$ 0.020	1.142	1.189	94.4	13.458	0.198	1.173	1.210	93.6
CIF2 $t$ = 4, $w=0$	23.228	23.239	0.011	1.591	1.667	94.6	23.467	0.239	1.618	1.674	94.0
CIF2 $t$ = 6, $w=0$	30.982	31.001	0.018	1.873	1.980	93.2	31.193	0.211	1.873	1.962	93.6
CIF2 $t$ = 8, $w=0$	37.130	37.225	0.094	2.066	2.226	94.0	37.357	0.227	2.033	2.176	92.8
CIF2 $t$ = 10, $w=0$	41.997	42.180	0.183	2.206	2.341	93.4	42.229	0.232	2.141	2.251	92.8
$\pi_{11}$	75.000	73.707	$-$ 1.293	5.390	5.656	94.0	73.820	$-$ 1.180	5.369	5.661	94.400
$\pi_{22}$	90.000	88.953	$-$ 1.047	2.125	2.250	92.4	88.832	$-$ 1.168	2.126	2.209	90.800
DIC criterion											
$\%$ of time the correct model is selected		75.0									
Mean difference (SPM-1 to SPM-2)		$-$ 1.5									

Results from 500 replications with each data set including 2000 individuals. The “true” marker evolution was based on linear splines with knots at 1 and 5 years since baseline and it was correctly specified in the fitted SPM-1 and SPM-2 models. “True” denotes the true parameter values; “Median” the mean of posterior medians over the 500 replications; “Bias” the mean bias for posterior median estimates; “ASD” the average posterior standard deviation, “MCSD” the empirical Monte Carlo deviation of estimates and “Coverage” the empirical coverage probability ($\%$) of posterior credible intervals.

**Table 2:** Simulation study results from fitted SPM-1 and SPM-2 models when the data are generated by the SPM-2 model under failure cause misclassification

Parameter	True	Median	Bias	ASD	MCSD	Coverage	Median	Bias	ASD	MCSD	Coverage
Longitudinal	Results from SPM-1						Results from SPM-2				
Intercept	12.850	12.845	$-$ 0.005	0.126	0.126	95.4	12.845	$-$ 0.005	0.126	0.126	95.8
Slope1 ($\beta_1$)	6.030	6.034	0.004	0.110	0.108	95.4	6.027	$-$ 0.003	0.110	0.108	95.8
Slope2 ($\beta_2$)	0.770	0.772	0.002	0.031	0.032	93.4	0.770	$-$ 0.000	0.031	0.032	93.2
Slope3 ($\beta_3$)	0.000	0.001	0.001	0.017	0.017	95.4	0.001	0.001	0.017	0.017	95.4
Cause1 (e.g. death)											
“True” marker value ($\alpha_{1}$)	$-$ 0.160	$-$ 0.144		0.017	0.017		$-$ 0.164	$-$ 0.004	0.020	0.020	95.4
Binary covariate ($\gamma_{1}$)	0.150	0.139		0.156	0.168		0.158	0.008	0.175	0.184	92.2
CIF1 $t$ = 2, $w=1$	8.319	8.239	$-$ 0.080	1.094	1.164	93.0	8.264	$-$ 0.055	1.110	1.173	93.6
CIF1 $t$ = 4, $w=1$	11.572	11.332	$-$ 0.241	1.430	1.501	93.4	11.373	$-$ 0.199	1.442	1.501	92.2
CIF1 $t$ = 6, $w=1$	13.469	13.208	$-$ 0.260	1.641	1.737	92.2	13.241	$-$ 0.228	1.647	1.718	92.4
CIF1 $t$ = 8, $w=1$	14.709	14.410	$-$ 0.299	1.783	1.921	92.0	14.447	$-$ 0.262	1.783	1.891	92.2
CIF1 $t$ = 10, $w=1$	15.521	15.135	$-$ 0.386	1.880	2.049	91.4	15.174	$-$ 0.346	1.876	2.014	91.2
CIF1 $t$ = 2, $w=0$	7.288	7.242	$-$ 0.046	1.034	1.074	92.8	7.202	$-$ 0.087	1.037	1.068	93.2
CIF1 $t$ = 4, $w=0$	10.196	9.991	$-$ 0.205	1.367	1.424	93.4	9.975	$-$ 0.221	1.368	1.408	93.4
CIF1 $t$ = 6, $w=0$	11.904	11.660	$-$ 0.244	1.575	1.639	93.2	11.656	$-$ 0.248	1.571	1.610	93.4
CIF1 $t$ = 8, $w=0$	13.027	12.734	$-$ 0.294	1.714	1.781	92.8	12.738	$-$ 0.290	1.705	1.745	93.0
CIF1 $t$ = 10, $w=0$	13.765	13.382	$-$ 0.382	1.809	1.879	92.2	13.390	$-$ 0.374	1.799	1.839	93.4
Cause2 (e.g., disengagement)											
“True” marker value ($\alpha_{2}$)	$-$ 0.020	$-$ 0.016		0.010	0.010		$-$ 0.019	0.001	0.012	0.012	95.8
Binary covariate ($\gamma_{2}$)	$-$ 0.150	$-$ 0.120		0.090	0.092		$-$ 0.150	$-$ 0.000	0.111	0.111	94.2
CIF2 $t$ = 2, $w=1$	12.861	12.944	0.083	1.142	1.197	94.4	12.872	0.011	1.177	1.217	94.2
CIF2 $t$ = 4, $w=1$	21.826	21.991	0.165	1.602	1.670	94.0	21.930	0.105	1.634	1.681	93.4
CIF2 $t$ = 6, $w=1$	28.518	28.644	0.126	1.886	1.948	94.2	28.631	0.114	1.901	1.944	94.4
CIF2 $t$ = 8, $w=1$	33.726	33.891	0.165	2.088	2.218	93.4	33.945	0.218	2.079	2.176	93.0
CIF2 $t$ = 10, $w=1$	37.837	38.056	0.219	2.239	2.405	92.6	38.167	0.330	2.205	2.330	92.4
CIF2 $t$ = 2, $w=0$	14.636	14.457	$-$ 0.179	1.203	1.254	94.6	14.639	0.003	1.245	1.292	94.4
CIF2 $t$ = 4, $w=0$	24.488	24.398	$-$ 0.090	1.647	1.697	92.8	24.593	0.105	1.680	1.720	93.0
CIF2 $t$ = 6, $w=0$	31.666	31.623	$-$ 0.043	1.912	1.994	92.2	31.780	0.114	1.920	1.977	93.0
CIF2 $t$ = 8, $w=0$	37.150	37.258	0.108	2.095	2.184	92.2	37.366	0.216	2.074	2.137	92.6
CIF2 $t$ = 10, $w=0$	41.417	41.694	0.277	2.230	2.337	93.4	41.750	0.333	2.182	2.256	93.2
$\pi_{11}$	75.000	73.411	$-$ 1.589	5.489	5.777	94.2	73.368	$-$ 1.632	5.480	5.746	93.6
$\pi_{22}$	90.000	88.744	$-$ 1.256	2.199	2.240	90.4	88.665	$-$ 1.335	2.194	2.219	90.2
DIC criterion											
$\%$ of time the correct model is selected		58.2									
Mean difference (SPM-1 to SPM-2)		0.36									

Results from 500 replications with each data set including 2000 individuals. The “true” marker evolution was based on linear splines with knots at 1 and 5 years since baseline and it was correctly specified in the fitted SPM-1 and SPM-2 models. True, denotes the true parameter values; Median, the mean of posterior medians over the 500 replications; Bias, the mean bias for posterior median estimates; ASD, the average posterior standard deviation; MCSD, the empirical Monte Carlo deviation of estimates; Coverage, the empirical coverage probability ($\%$) of posterior credible intervals.

To investigate the added value of double sampling, we performed an additional simulation study in which only the reported failure causes are used in the model. The main conclusion was that the estimated association parameters can be seriously biased (Tables S9 and S10 of the Supplementary material available at *Biostatistics* online). As expected, when the true failure cause was not misclassified (Tables S11 and S12 of the Supplementary material available at *Biostatistics* online), the model performance was satisfactory.

## 6. Application

The proposed methodology was applied to data from the East Africa International Epidemiologic Databases to Evaluate AIDS (IeDEA) Regional Consortium. We aimed to jointly estimate the CD4 evolution after ART initiation and the CIFs for death and disengagement from care. To adjust for potential death under-reporting, we incorporated information from double sampling, that is, a random sample from disengaged patients whose true vital status was ascertained by tracing these patients in the community. In this application, misclassification can be safely assumed to be unidirectional, that is, a true death can be incorrectly classified as a disengagement from care but a true disengagement from care cannot be misclassified as an observed death. Thus, assuming nondifferential misclassification, there is actually only one misclassification parameter $\pi_{11}$, with $K_{i}=1$ and $K_{i}=2$ corresponding to death and disengagement from care, respectively.

To illustrate the proposed methodology, we used a 60$\%$ random sample of women aged from 35 to 45 years (the most frequent covariate pattern in the data), leading to 8005 patients included. 3275 (40.9$\%$) and 273 (3.4$\%$) disengagements from care and deaths were reported, respectively, whereas 4457 (55.7$\%$) were event free at the end of the follow-up (noninformative right censoring). In 443 (13.5$\%$) patients out of those who were reported as disengaged from care, the true vital status was ascertained through double sampling, and among them, there were 80 (18.1$\%$) hidden deaths. As mortality among doubly sampled individuals was very high, disengagement from care should not be treated as a noninformative censoring event. The median (interquartile range) number of CD4 observations per individual, CD4 count at ART initiation, and follow-up time were 2.0 (1.0, 5.0), 163 (80, 264) cells/$\mu$L, and 1.3 (0.4, 2.7) years, respectively.

To model the CD4 evolution, we used an LMM on the square root scale using natural cubic splines of time with knots at 0.55, 1.25, and 2.35 years since ART initiation for both the fixed and random effects. The psition of knots was based on the quartiles of the measurement times. Baseline CIF levels for death and care disengagement were modeled through cubic B-splines with 2 and 3 knots, respectively. The model for each cause was selected among SPM-1 and SPM-2 with $c_k \in \{ 0.25,0.5,0.75,\ldots,2\}$ by optimizing the DIC, assuming nondifferential misclassification, $\pi_{11}$. The DIC criterion was optimized at $c_{1}=0.5$ for death and the subdistribution hazard model (SPM-1) for disengagement from care. We also examined whether $\pi_{11}$ depends on the time since ART initiation and the last observed $\sqrt{\text{CD4}}$, but the effects were nonsignificant, thus, the final model assumed nondifferential misclassification. For comparison, we also fitted the corresponding model without misclassification. Details on the prior distributions used and the specification of the MCMC algorithm are presented in Section S8 of the Supplementary material available at *Biostatistics* online.

**Figure 1:**
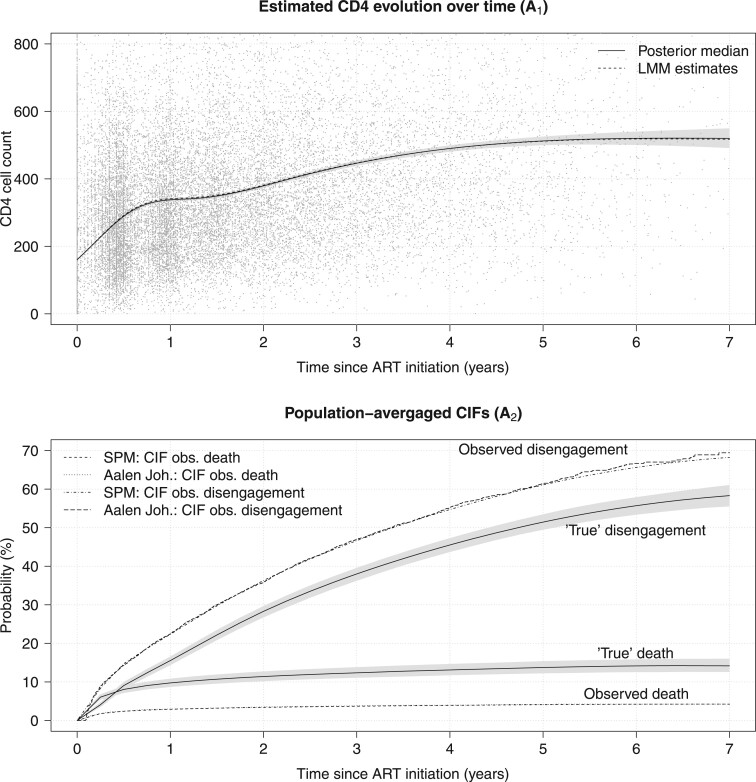
Estimated CD4 evolution and population-averaged CIFs based on the proposed model assuming $c_{1}=0.5$ for death and the proportional subdistribution hazard model (SPM-1) for disengagement from care, taking into account the double sampling data, applied to East Africa IeDEA data. Shades in gray show pointwise credible intervals. $\boldsymbol{A}_{1}$: estimated CD4 evolution over time since ART initiation (CD4 counts up to 800 cells/$\mu$L are shown). $\boldsymbol{A}_{2}$: population-averaged CIFs for death and disengagement from care along with the corresponding CIFs for an observed death and disengagement from care.

The main results are presented in Table 3. There is substantial underestimation of mortality, as only 29.79$\%$ of the estimated “true” deaths were reported. The fixed-effect estimates and the effects of the “true” marker value on mortality were roughly similar between the two models. Of note, the corresponding effects on the CIF of disengagement from care were discordant, that is, an increase of $m_{i}(t)$ was associated with a significantly greater subdistribution hazard for disengagement from care when death misclassification was accounted for through double sampling, whereas the model using the observed failure causes implied no effect. This finding may be attributed to the considerable proportion of hidden deaths among the patients flagged as lost to clinic, as deceased patients had lower “true” marker values on average. From now on, we focus only on the model accounting for misclassification. The estimated CD4 evolution is presented in Figure 1$A_{1}$. After an initial very rapid increase from ART initiation to around 6 months, CD4 cell counts continued increasing but at a lower rate, until reaching a plateau at about 6 years since ART initiation. The estimates from the proposed model were similar to those obtained by a corresponding LMM (Figure 1$\mathbf{A}_{1}$).

**Table 3:** Results from the proposed model assuming $c_{1}=0.5$ for death and the proportional subdistribution hazard model (SPM-1) for disengagement from care applied to East Africa IeDEA data

	Misclassification				No Misclassification			
Parameter	Median	SD	LB	UB	Median	SD	LB	UB
Longitudinal								
Intercept	12.68	0.06	12.55	12.80	12.68	0.07	12.55	12.80
$\beta_1$	5.46	0.09	5.28	5.65	5.45	0.09	5.28	5.63
$\beta_2$	7.96	0.17	7.62	8.29	7.87	0.17	7.54	8.20
$\beta_3$	15.24	0.21	14.84	15.67	15.16	0.21	14.75	15.57
$\beta_4$	7.32	0.33	6.67	7.96	7.15	0.32	6.52	7.81
Cause1 (Death)								
“True” marker value, $\alpha_{1}$	$-$ 0.17	0.01	$-$ 0.20	$-$ 0.15	$-$ 0.18	0.01	$-$ 0.21	$-$ 0.15
Cause2 (Disengagement)								
“True” marker value sHR, $\exp(\alpha_{2})$	1.04	0.01	1.03	1.05	1.00	0.00	0.99	1.01
$\pi_{11}$	29.79	2.04	25.98	34.02				

The mean evolution was based on natural cubic splines of time with knots at 0.55, 1.25, and 2.35 years since ART initiation while the random-effects specification was based on a random intercept and slope structure. “Median,” “SD,” “LB,” and “UB” denote the posterior median, standard deviation, 2.5$\%$ and 97.5$\%$ quantiles, respectively. sHR, denotes the subdistribution hazard ratio; $\pi_{11}$, probability of correctly classifying a death.

**Figure 2:**
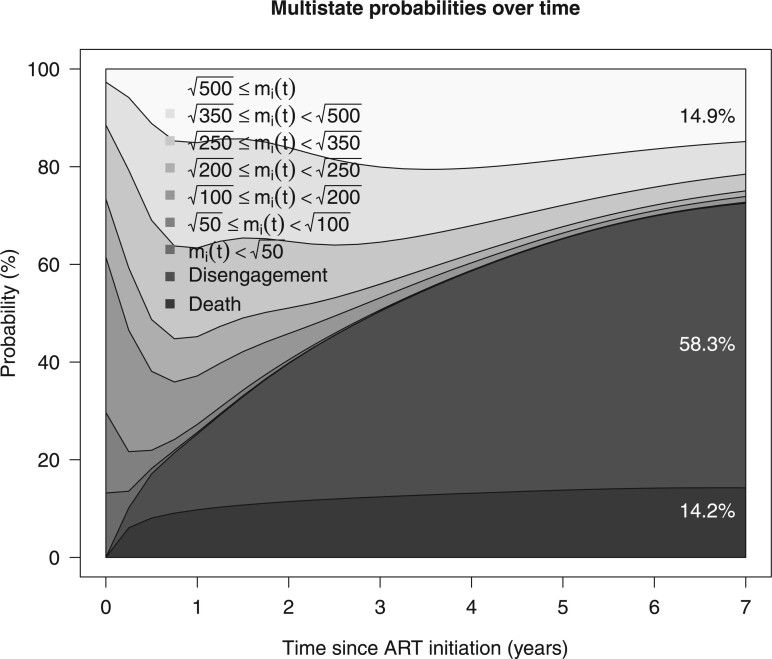
Stacked multistate probability plot of latent marker states and competing risks of death and disengagement from care over time since ART initiation based on the proposed model assuming $c_{1}=0.5$ for death and the proportional subdistribution hazard model (SPM-1) for disengagement from care, taking into account the double sampling data, applied to East Africa IeDEA data. The corresponding state occupancy probabilities are visualized through the difference between two adjacent curves with different shades of gray.

The results for the estimated population-averaged CIFs are presented in Figure 1$A_{2}$. Also shown is the corresponding CIFs ignoring potential misclassification, that is, $\Pr(T_{i}^{\star}\leq t,\tilde{K}_{i}=1;\boldsymbol{\theta},\boldsymbol{\theta}_{\rm misc})=\Pr(T_{i}^{\star}\leq t,K_{i}=1;\boldsymbol{\theta})\pi_{11}$ and $\Pr(T_{i}^{\star}\leq t,\tilde{K}_{i}=2;\boldsymbol{\theta},\boldsymbol{\theta}_{\rm misc})=\Pr(T_{i}^{\star}\leq t,K_{i}=1;\boldsymbol{\theta})(1-\pi_{11})+\Pr(T_{i}^{\star}\leq t,K_{i}=2;\boldsymbol{\theta})$. These estimates are in close agreement with the corresponding Aalen–Johansen estimates, implying that the model is flexible enough to model the observed patterns of events. As expected, ignoring misclassification led to underestimation of mortality and overestimation of the risk for disengagement from care. In Figure 2, we present multistate probabilities for all states simultaneously (CD4 states, death, and disengagement from care). By 7 years since ART initiation, we estimated that 14.2$\%$ (95$\%$ CI 12.6–16.0) had died and 58.3$\%$ (95$\%$ CI 55.5–61.1) had disengaged from care. The corresponding results by baseline CD4 state are presented in Figure S5 of the Supplementary material available at *Biostatistics* online. The CIF of death at 7 years for those starting at $m_{i}(0)<\sqrt{50}$ was 30.9$\%$ (95$\%$ CI 27.6–34.3), remarkably higher than that of the remaining baseline CD4 states. Given $m_{i}(0) <\sqrt{50}$, the transition probability to $m_{i}(5)\geq\sqrt{500}$ at 5 years while being event free was low 6.7$\%$ (95$\%$ CI 5.6–7.9), whereas the corresponding probability for those with $\sqrt{50}\leq m_{i}(0)<\sqrt{100}$ was 12.5$\%$ (95$\%$ CI 11.3–13.6). Among those with 100–200 observed baseline CD4 cells/$\mu$L who were alive and progressed to 200–250 or $>$ 500 CD4 cells/$\mu$L at 1 year since ART initiation, the conditional probabilities of dying within the next year were 2.8$\%$ (95$\%$ CI 2.1–3.5) and 1.1$\%$ (95$\%$ CI 0.8–1.4), respectively.

## 7. Discussion

In this article, we proposed a flexible and unified class of models to jointly model a normally distributed marker over time and competing risks using CIFs for the survival submodels, with inference on model parameters obtained through a hybrid MCMC algorithm. The proposed models assume that the CIFs depend on the “true” marker value over time, $m_{i}(t)$, thus the association between the marker and survival processes is induced via the random effects. Hence, the proposed models lie within the family of SPMs. Most competing-risk SPMs rely on cause-specific hazards; but CIF estimates may be of particular interest when the focus lies on prognosis. Though it is feasible to derive CIF estimates based on estimated cause-specific hazards, it requires complex integration, being particularly challenging in joint models. In contrast, under our proposed approach, the effects on the CIFs are described in a direct and straightforward way. To model the link functions, we used the generalized odds rate transformation, with the proportional subdistribution hazards model (Deslandes and Chevret, 2010; Fine and Gray, 1999) being a special case. Due to potential failure cause misclassification in our motivating example, we extended our methodology by incorporating information from doubly sampled patients, that is, a random sample from patients to whom a gold standard diagnostic procedure was performed. Accounting for misclassification, based solely on the joint model, we also estimated multistate probabilities jointly defined by marker data and competing risks. A simulation study was carried out to examine the performance of the methodology, indicating that the model performance is satisfactory when the marker trajectory and the association structure between the marker and the CIFs are correctly specified but the link function is misspecified (using two scenarios for $c_{k}$). The proposed models were also fitted to data from the IeDEA study using CD4 count data from ART initiation until the occurrence of death or disengagement from care. To reduce computation time, a 60$\%$ random sample was used. Ignoring double sampling led to seriously underestimated mortality, whereas it implied no effect of the “true” CD4 count on the risk for disengagement from care, but after adjusting for misclassification, moderate, but statistically significant, positive correlation was found. We suppose that the latter discrepancy could be at least partly explained by the considerable proportion of deaths among those observed to disengage from care.

One important issue when specifying models for CIFs is that the all-cause CIF should be bounded by 1 at each failure time. When there are no random effects, this can be dealt with in the maximization process, but how to address this issue in the presence of random effects has not been resolved in the literature. Our model assumed $\tau_{i}(\boldsymbol{\beta},\boldsymbol{\theta}_{s},\boldsymbol{b}_{i})$ as the upper bound of the survival time, which mathematically led to zero likelihood when the constraint is violated. This was equivalent to introducing an indicator function in the likelihood in the parameter estimation process. However, to further derive population-averaged CIFs and marker state probabilities, integration over the random effects is required, and thus, CIFs should be evaluable at any random-effect value drawn from its prior $N(\boldsymbol{0},\boldsymbol{D})$. Thus, having an explicitly defined model for the CIFs accounting for the constraints, population-averaged quantities can be estimated directly.

Our approach of multistate modeling differs from standard approaches (e.g., Putter *and others*, 2007) in which states are assumed to be directly observed and usually rely on the Markov assumption. In contrast, under our approach, marker states were not assumed to be directly observed, with the computations being solely based on the assumed joint model by formally deriving posterior samples for multistate probabilities. Our approach has also substantial differences from the work by Hu *and others* (2012); they proposed a two-stage approach where marker trends are first estimated using subject-specific regression models and then marker states are evaluated by averaging over individuals. Thus, the effects of the marker on the competing risks are not modeled explicitly, whereas individuals with highly irregular visit times or just one marker measurement may cause additional difficulties.

In this work, we have adjusted for failure cause misclassification through double sampling. The issue of missing failure cause in a joint modeling setting has been addressed before (Sheikh *and others*, 2021). However, there are major differences with our approach as Sheikh *and others* (2021) did not consider reported failure causes and used cause-specific hazards.

The proposed methodology relies on parametric assumptions, thus, as always with parametric approaches, certain assumptions may not hold. Among all model assumptions, though, the ones that are most difficult to verify are perhaps those related to missing data mechanisms, for example, the proposed models, lying within the general class of SPMs, assume that missing marker data after the first occurring event are MNAR. However, the question of missing data being MAR or MNAR is complex and probably depends on the richness of the design. Death is often considered to cause MNAR marker data as it corresponds to underlying disease progression that is unlikely to be reflected in the observed marker data, measured at prespecified time points; the nature of the dropout mechanism due to disengagement is less clear though. It has been shown that if missing marker data are MAR, fixed-effect estimates from specific SPMs are susceptible to bias (Thomadakis *and others*, 2019). In our application, we feel that this is unlikely as the fixed-effects estimates from the proposed model were in line with the corresponding ones from the LMM.

There are some extensions that could be incorporated into the proposed methodology: for example, it would be interesting to provide dynamic survival predictions (Rizopoulos, 2012) or use more flexible forms for the association between the marker and the CIFs. Moreover, some aspects of the proposed methodology may require further consideration. For example, it would be interesting to evaluate the model performance under misspecification of the misclassification model, different true parameter values, and different percentages of doubly sampled individuals.

To sum up, we have proposed a flexible class of SPMs to jointly model a normally distributed marker and competing risks using CIFs in the survival submodels, extended to account for potential failure cause misclassification. As most approaches in the literature rely on cause-specific hazards, our proposed approach can be a useful alternative when the focus is on identifying risk factors for the risk of an event.

## Supplementary Material

kxac043_Supplementary_Data

## Supplementary material

Supplementary material is available online at *Biostatistics* online and software in the form of R code is available at https://github.com/cthomadak/JointModelCumInc.
