# Supplementary material for: Joint modeling of longitudinal and competing-risk data using cumulative incidence functions for the failure submodels accounting for potential failure cause misclassification through double sampling
Source: Biostatistics. 2022 Nov 4;25(1):80–97. doi: 10.1093/biostatistics/kxac043 (PMC10724131; doi:10.1093/biostatistics/kxac043)
Supplement: kxac043_Supplementary_Data [file kxac043_supplementary_data.pdf]

Biostatistics (2022), **0**, 0, *pp.* **??–??**
doi:10.1093/biostatistics/JointCumInc˙Thomadakis˙supp˙bio

Web-based supporting materials for (Joint modeling of longitudinal and competing-risks data using cumulative incidence functions for the failure submodels accounting for potential failure cause misclassification through double sampling) by Christos Thomadakis, Loukia Meligkotsidou, Constantin T. Yiannoutsos, and Giota Touloumi

Christos Thomadakis*

Department of Hygiene, Epidemiology and Medical Statistics, National and Kapodistrian University of Athens, Athens, Greece

cthomadak@med.uoa.gr

Loukia Meligkotsidou

Department of Mathematics, National and Kapodistrian University of Athens, Athens, Greece

Constantin T. Yiannoutsos

Department of Biostatistics, Indiana University Fairbanks School of Public Health Indianapolis, U.S.A.

Giota Touloumi

Department of Hygiene, Epidemiology and Medical Statistics, National and Kapodistrian University of Athens, Athens, Greece

Summary

*To whom correspondence should be addressed.

© The Author 2022. Published by Oxford University Press. All rights reserved. For permissions, please e-mail: journals.permissions@oup.com

This document is the on-line supplementary material for the paper entitled "Joint modeling of longitudinal and competing-risks data using cumulative incidence functions for the failure submodels". Section 1 presents more details regarding the application of the MCMC algorithm used. In Section 2, we derive the probability of the "true" failure cause given all the observed data based on the assumed shared parameter model. The algorithm to update the values of the misclassification parameters is provided in Section 3, and the marginalized DIC criterion is outlined in Section 4. In Section 5, we derive population-averaged CIF estimates conditional on observed marker states. In Section 6, details about the design of the simulation study are shown and in Section 7, we present additional results for the simulation studies. Finally, in Section 8, additional results from the model applied to data from the East African IeDEA study are presented.

Key words: Bayesian statistics; Cumulative incidence function; Joint modelling; Misclassification; Multi-state model; Shared parameter models

1. Detailed description of the MCMC algorithm

1.1 *Outline of the applied MCMC algorithm*

In this section, we describe the MCMC algorithm in more detail. Given the likelihood and prior specification, it follows that the conditional posterior distribution of ω is Gamma$\{\frac{n}{2}+\lambda_1,\lambda_2+\frac{1}{2}\sum_{i=1}^{N}(\boldsymbol{Y}_i-\boldsymbol{X}_i\boldsymbol{\beta}-\boldsymbol{Z}_i\boldsymbol{b}_i)^\top(\boldsymbol{Y}_i-\boldsymbol{X}_i\boldsymbol{\beta}-\boldsymbol{Z}_i\boldsymbol{b}_i)\}$, where $n=\sum_{i=1}^{N}n_i$ is the total sample size. The corresponding conditional posterior for $\boldsymbol{D}$ is $IW(\boldsymbol{A}+\sum_{i=1}^{N}\boldsymbol{b}_i\boldsymbol{b}_i^\top, df+N)$. For the remaining parameters, we use Metropolis-Hasting schemes. For $\boldsymbol{\beta}$, we use the conditional posterior distribution given the marker as the proposal distribution, i.e. $q(\boldsymbol{\beta}^{can}|\mathcal{D},\boldsymbol{b};\omega)\sim N(\boldsymbol{\mu}_1,\boldsymbol{C}_1)$, where $\boldsymbol{C}_1=(\boldsymbol{C}_0^{-1}+\omega\sum_{i=1}^{N}\boldsymbol{X}_i^\top\boldsymbol{X}_i)^{-1}$ and $\boldsymbol{\mu}_1=\boldsymbol{C}_1\{\boldsymbol{C}_0^{-1}\boldsymbol{\mu}_0+\omega\sum_{i=1}^{N}\boldsymbol{X}_i^\top(\boldsymbol{Y}_i-\boldsymbol{Z}_i\boldsymbol{b}_i)\}$. It can be

easily shown that the acceptance probability is equal to

$$p = \min\left\{1, \prod_{i=1}^{N} f\{T_i, K_i | M_i^{can}(T_i), \boldsymbol{w}_i; \boldsymbol{\theta}_s\} / \prod_{i=1}^{N} f\{T_i, K_i | M_i(T_i), \boldsymbol{w}_i; \boldsymbol{\theta}_s\}\right\},$$

where $M_i^{can}(T_i)$ and $M_i(T_i)$ denote the "true" marker values up to T_i evaluated at the candidate, $\boldsymbol{\beta}^{can}$, and current MCMC value, $\boldsymbol{\beta}$, respectively.

The conditional posterior distribution of the random effects is proportional to

$$\begin{aligned} f(\boldsymbol{b}_i | \mathcal{D}; \boldsymbol{\theta}) \propto & \exp\left\{-\frac{1}{2}\boldsymbol{b}_i^\top(\boldsymbol{D}^{-1} + \omega \boldsymbol{Z}_i^\top \boldsymbol{Z}_i)\boldsymbol{b}_i + \omega \boldsymbol{b}_i^\top \boldsymbol{Z}_i^\top(\boldsymbol{Y}_i - \boldsymbol{X}_i\boldsymbol{\beta})\right\} \\ & \times \prod_{k=1}^{K} f_{ik}^{M}\{T_i | M_i(T_i), \boldsymbol{w}_{ik}; \boldsymbol{\theta}_{sk}\}^{\delta_{ik}} \left[1 - \sum_{k=1}^{K} F_{ik}^{M}\{T_i | M_i(T_i), \boldsymbol{w}_{ik}; \boldsymbol{\theta}_{sk}\}\right]^{1-\delta_i} \\ & \times I\left[\sum_{k=1}^{K} F_{ik}^{M}\{T_i | M_i(T_i), \boldsymbol{w}_{ik}; \boldsymbol{\theta}_{sk}\} < 1\right], \end{aligned} \tag{1.1}$$

where $f_{ik}^M\{x | M_i(x), \boldsymbol{w}_{ik}; \boldsymbol{\theta}_{sk}\} = \partial F_{ik}^M\{x | M_i(x), \boldsymbol{w}_{ik}; \boldsymbol{\theta}_{sk}\} \partial x$. To update the value of $\boldsymbol{b}_i$ in each cycle of the MCMC algorithm, starting from the posterior mode using only the marker model, i.e. $\boldsymbol{\mu}_{\boldsymbol{b}_i} = (\boldsymbol{D}^{-1} + \omega \boldsymbol{Z}_i^\top \boldsymbol{Z}_i)^{-1} \omega \boldsymbol{Z}_i^\top(\boldsymbol{Y}_i - \boldsymbol{X}_i\boldsymbol{\beta})$, we carry out a single Newton Raphson step to maximize (1.1), i.e. $\boldsymbol{b}_i^\star = \boldsymbol{\mu}_{\boldsymbol{b}_i} + \mathcal{I}(\boldsymbol{\mu}_{\boldsymbol{b}_i})^{-1}\mathcal{U}(\boldsymbol{\mu}_{\boldsymbol{b}_i})$, where $\mathcal{U}(\boldsymbol{b}_i) = \frac{\partial \log f(\boldsymbol{b}_i | \mathcal{D}; \boldsymbol{\theta})}{\partial \boldsymbol{b}_i}$ and $\mathcal{I}(\boldsymbol{b}_i) = -\frac{\partial^2 \log f(\boldsymbol{b}_i | \mathcal{D}; \boldsymbol{\theta})}{\partial \boldsymbol{b}_i \partial \boldsymbol{b}_i^\top}$. However, in very rare cases, $\boldsymbol{\mu}_{\boldsymbol{b}_i}$ might not fulfill the boundness constraint, i.e. it might be the case that $f(\boldsymbol{\mu}_{\boldsymbol{b}_i} | \mathcal{D}; \boldsymbol{\theta}) = 0$. To avoid problems with the logarithm of a function that is equal to zero, we calculated $\mathcal{U}(\boldsymbol{b}_i)$ and $\mathcal{I}(\boldsymbol{b}_i)$ ignoring the boundness constraint and evaluated $\frac{\partial \log\left[1 - \sum_{k=1}^{K} F_{ik}^M\{T_i | M_i(T_i), \boldsymbol{w}_{ik}; \boldsymbol{\theta}_{sk}\}\right]}{\partial \boldsymbol{b}_i}$ by $-\frac{\sum_{k=1}^{K} \partial F_{ik}^M\{T_i | M_i(T_i), \boldsymbol{w}_{ik}; \boldsymbol{\theta}_{sk}\} \partial \boldsymbol{b}_i}{1 - \sum_{k=1}^{K} F_{ik}^M\{T_i | M_i(T_i), \boldsymbol{w}_{ik}; \boldsymbol{\theta}_{sk}\}}$, which is always finite. Let $\boldsymbol{b}_i^{can}$ be a value for $\boldsymbol{b}_i$ proposed using the density $q(\boldsymbol{b}_i^{can} | \mathcal{D}; \boldsymbol{\theta}) \sim N\left\{\boldsymbol{b}_i^\star, (\boldsymbol{D}^{-1} + \omega \boldsymbol{Z}_i^\top \boldsymbol{Z}_i)^{-1}\right\}$; note that the proposal distribution does not depend on the current value of $\boldsymbol{b}_i$, though it does depend on the current values of the remaining parameters, $\boldsymbol{\theta}_s$ and $\boldsymbol{\theta}_L$. The Metropolis-Hastings acceptance probability is therefore equal to

$$p = \min\left\{1, \frac{f(\boldsymbol{b}_i^{can} | \mathcal{D}; \boldsymbol{\theta})}{f(\boldsymbol{b}_i | \mathcal{D}; \boldsymbol{\theta})} \times \frac{q(\boldsymbol{b}_i | \mathcal{D}; \boldsymbol{\theta})}{q(\boldsymbol{b}_i^{can} | \mathcal{D}; \boldsymbol{\theta})}\right\}.$$

Thus, if the all-cause CIF is not bounded by 1 at $\boldsymbol{b}_i^{can}$, the acceptance probability is equal to zero as $f(\boldsymbol{b}_i^{can} | \mathcal{D}; \boldsymbol{\theta})$ equals zero in this case.

The conditional posterior distribution of the survival model parameters, $\boldsymbol{\theta}_s$, is proportional to

$$f(\boldsymbol{\theta}_s|\mathcal{D},\boldsymbol{b};\boldsymbol{\beta}) \propto \prod_{i=1}^{N}\left(\prod_{k=1}^{K} f_{ik}^{M}\{T_i|M_i(T_i),\boldsymbol{w}_{ik};\boldsymbol{\theta}_{sk}\}^{\delta_{ik}}\left[1-\sum_{k=1}^{K}F_{ik}^{M}\{T_i|M_i(T_i),\boldsymbol{w}_{ik};\boldsymbol{\theta}_{sk}\}\right]^{1-\delta_i} \times I\left[\sum_{k=1}^{K}F_{ik}^{M}\{T_i|M_i(T_i),\boldsymbol{w}_{ik};\boldsymbol{\theta}_{sk}\}<1\right]\right)f(\boldsymbol{\theta}_s), \tag{1.2}$$

where $f(\boldsymbol{\theta}_s)$ is the prior distribution of $\boldsymbol{\theta}_s$. We update the survival parameters $\boldsymbol{\theta}_{sk}$, $k=1,\ldots,K$, separately, performing BFGS updates (Thisted, 1988) maximising $f(\boldsymbol{\theta}_{sk}|\mathcal{D},\boldsymbol{b};\boldsymbol{\beta},\boldsymbol{\theta}_{sj},j\neq k)$, starting from the current value of the chain for $\boldsymbol{\theta}_{sk}$; note also that $f(\boldsymbol{\theta}_{sk}|\mathcal{D},\boldsymbol{b};\boldsymbol{\beta},\boldsymbol{\theta}_{sj},j\neq k)$ is proportional to (1.2). Thus, this method is based on the approach proposed by Gamerman (1997). The BFGS algorithm can be briefly described as follows: Given an approximate information matrix $\boldsymbol{A}_k^{(j)}$ $(j=0,1,\ldots)$, we compute a new point

$$\boldsymbol{\theta}_{sk}^{(j+1)} = \boldsymbol{\theta}_{sk}^{(j)} + \{\boldsymbol{A}_k^{(j)}\}^{-1}\mathcal{U}\{\boldsymbol{\theta}_{sk}^{(j)}\},$$

where $\mathcal{U}(\boldsymbol{\theta}_{sk}) = \frac{\partial \log f(\boldsymbol{\theta}_{sk}|\mathcal{D},\boldsymbol{b};\boldsymbol{\beta},\boldsymbol{\theta}_{sj},j\neq k)}{\partial \boldsymbol{\theta}_{sk}}$ and $\boldsymbol{\theta}_{sk}^{(0)}$ is equal to the current value of the chain for $\boldsymbol{\theta}_{sk}$. The approximate information matrix $\boldsymbol{A}_k^{(j)}$ is also updated using the formula

$$\boldsymbol{A}_k^{(j+1)} = \boldsymbol{A}_k^{(j)} - \frac{\boldsymbol{u}\boldsymbol{u}^\top}{\boldsymbol{u}^\top\boldsymbol{s}} - \frac{\boldsymbol{A}_k^{(j)}\boldsymbol{s}\boldsymbol{s}^\top\boldsymbol{A}_k^{(j)}}{\boldsymbol{s}^\top\boldsymbol{A}_k^{(j)}\boldsymbol{s}},$$

where $\boldsymbol{u} = \mathcal{U}\{\boldsymbol{\theta}_{sk}^{(j+1)}\} - \mathcal{U}\{\boldsymbol{\theta}_{sk}^{(j)}\}$ and $\boldsymbol{s} = \boldsymbol{\theta}_{sk}^{(j+1)} - \boldsymbol{\theta}_{sk}^{(j)}$. To find reasonably good approximations of the information matrices, before starting the MCMC algorithm, we maximize (1.2) using the estimates of the fixed and random effects from the LMM, resulting in an estimate $\boldsymbol{\theta}_s^{LMM}$, and calculate the corresponding information matrix $\boldsymbol{A}$ at $\boldsymbol{\theta}_s^{LMM}$. Then we use the submatrix $\boldsymbol{A}_k$ associated with the parameter $\boldsymbol{\theta}_{sk}$ as the initial approximate information matrix $\boldsymbol{A}_k^{(0)}$. The main advantage of this procedure is that the computation of the Hessian matrix in (1.2) is avoided, which reduces the computational burden. Let $\boldsymbol{\theta}_{sk}^{\star}$ be the value obtained after performing some BFGS steps. Moreover, let $\boldsymbol{\theta}_{sk}^{can}$ be a value for $\boldsymbol{\theta}_{sk}$ proposed using the density

$q(\boldsymbol{\theta}_{sk}^{can}|\mathcal{D}, \boldsymbol{b}; \boldsymbol{\beta}, \boldsymbol{\theta}_{sk}, \boldsymbol{\theta}_{sj}, j \neq k) \sim t_{10}\left\{\boldsymbol{\theta}_{sk}^{\star}, (\boldsymbol{A}_k^{(0)})^{-1}\right\}$, where t_{10} denotes the multivariate Student-t distribution with location parameter and scale matrix equal to $\boldsymbol{\theta}_{sk}^{\star}$ and $(\boldsymbol{A}_k^{(0)})^{-1}$, respectively, with 10 degrees of freedom. The acceptance probability is equal to

$$p = \min\left\{1, \frac{f(\boldsymbol{\theta}_{sk}^{can}|\mathcal{D}, \boldsymbol{b}; \boldsymbol{\beta}, \boldsymbol{\theta}_{sj}, j \neq k)}{f(\boldsymbol{\theta}_{sk}|\mathcal{D}, \boldsymbol{b}; \boldsymbol{\beta}, \boldsymbol{\theta}_{sj}, j \neq k)} \times \frac{q(\boldsymbol{\theta}_{sk}|\mathcal{D}, \boldsymbol{b}; \boldsymbol{\beta}, \boldsymbol{\theta}_{sk}^{can}, \boldsymbol{\theta}_{sj}, j \neq k)}{q(\boldsymbol{\theta}_{sk}^{can}|\mathcal{D}, \boldsymbol{b}; \boldsymbol{\beta}, \boldsymbol{\theta}_{sk}, \boldsymbol{\theta}_{sj}, j \neq k)}\right\}.$$

Recall that, if the candidate value, $\boldsymbol{\theta}_{sk}^{can}$, yields an all-cause CIF greater than 1, the acceptance probability is equal to 0. We also need to point out that, to calculate the proposal ratio, we need to perform the inverse BFGS step, starting from the proposed value $\boldsymbol{\theta}_{sk}^{can}$, and recalculate the value of $\boldsymbol{\theta}_{sk}^{\star}$ in the numerator of the posterior ratio.

When there is failure cause misclassification, though, the initial information matrix $\boldsymbol{A}_k^{(0)}$ obtained by maximising the posterior distribution $f(\boldsymbol{\theta}_s|\mathcal{D}, \boldsymbol{b}^{LMM}; \boldsymbol{\beta}^{LMM})$ using the observed failure causes may not be a good approximation. To address this issue, we perform a quick iterative procedure before starting the MCMC algorithm: (a) we maximize $f(\boldsymbol{\theta}_s|\mathcal{D}, \boldsymbol{b}^{LMM}; \boldsymbol{\beta}^{LMM})$ using simulated missing failure causes $\{K_i : i \in \mathcal{I}_{mis}\}$ and (b) based on the current mode of $f(\boldsymbol{\theta}_s|\mathcal{D}, \boldsymbol{b}^{LMM}; \boldsymbol{\beta}^{LMM})$ we carry out an approximate Gibbs sampling procedure of 100 iterations for the conditional posterior distribution of $(\boldsymbol{\theta}_{misc}, \{K_i : i \in \mathcal{I}_{mis}\})$ by repeatedly simulating the missing failure causes and locating the mode of the conditional posterior distribution of $\boldsymbol{\theta}_{misc}$ given the simulated failure causes $\{K_i : i \in \mathcal{I}_{mis}\}$. This approach is repeated 5 times and $\boldsymbol{A}_k^{(0)}$ corresponds to the information matrix for $\boldsymbol{\theta}_{sk}$ at the last iteration. In our data examples, repeating this procedure 5 times led to adequate initial information matrices $\boldsymbol{A}_k^{(0)}$, $k = 1, 2, \ldots, K$.

1.2 *Initial values in the MCMC algorithm*

We used fixed initial values $\boldsymbol{\beta}^{(0)}$, $\boldsymbol{D}^{(0)}$, and $\omega^{(0)}$ for $\boldsymbol{\beta}$, $\boldsymbol{D}$, and ω, respectively. The initial values for the random effects were simulated from $N\{\boldsymbol{b}_i^{LMM}, (\{\boldsymbol{D}^{(0)}\}^{-1} + \omega^{(0)}\boldsymbol{Z}_i^{\top}\boldsymbol{Z}_i)^{-1}\}$, where $\boldsymbol{b}_i^{LMM}$ stands for the predictions of the random effects based on the LMM. Then we obtained initial

values for $\boldsymbol{\theta}_s$ by maximising (1.2) given the initial values of $\{\boldsymbol{b}_i\}_{i=1}^N$ and $\boldsymbol{\beta}$. Under failure cause misclassification, after taking the "true" failure causes from doubly sampled patients into account, we obtained initial values for $\boldsymbol{\theta}_{misc}$ based on the full conditional posterior distribution of $\boldsymbol{\theta}_{misc}$.

1.3 *Full conditional posterior distributions for the SPM-1 model*

The posterior distribution of the random effects under the SPM-1 model is proportional to

$$f(\boldsymbol{b}_i|\mathcal{D};\boldsymbol{\theta}) \propto \exp\Bigg[-\frac{1}{2}\boldsymbol{b}_i^\top(\boldsymbol{D}^{-1}+\omega\boldsymbol{Z}_i^\top\boldsymbol{Z}_i)\boldsymbol{b}_i + \omega\boldsymbol{b}_i^\top\boldsymbol{Z}_i^\top(\boldsymbol{Y}_i-\boldsymbol{X}_i\boldsymbol{\beta}) + \sum_{k=1}^K \delta_{ik}\left\{\alpha_k\boldsymbol{b}_i^\top\boldsymbol{z}_i(T_i) - S_{ik}(\boldsymbol{b}_i)\right\} + (1-\delta_i)\log\left\{1-K+\sum_{k=1}^K e^{-S_{ik}(\boldsymbol{b}_i)}\right\}\Bigg] I\left[\sum_{k=1}^K F_{ik}^M\{T_i|M_i(T_i),\boldsymbol{w}_{ik};\boldsymbol{\theta}_{sk}\}<1\right], \qquad (1.3)$$

where $S_{ik}(\boldsymbol{b}_i)=\int_0^{T_i} e^{\boldsymbol{B}_k^\top(s)\boldsymbol{\psi}_k+\boldsymbol{\gamma}_k^\top\boldsymbol{w}_{ik}+\alpha_k m_i(s)}ds$. Note that (1.3) is equal to zero when the model-based CIF is not bounded by one due to the indicator function $I\left[\sum_{k=1}^K F_{ik}^M\{T_i|M_i(T_i),\boldsymbol{w}_{ik};\boldsymbol{\theta}_{sk}\}<1\right]$. It can be easily shown that

$$\mathcal{U}(\boldsymbol{b}_i) = -(\boldsymbol{D}^{-1}+\omega\boldsymbol{Z}_i^\top\boldsymbol{Z}_i)\boldsymbol{b}_i + \omega\boldsymbol{Z}_i^\top(\boldsymbol{Y}_i-\boldsymbol{X}_i\boldsymbol{\beta}) + \sum_{k=1}^K\delta_{ik}\left\{\alpha_k\boldsymbol{z}_i(T_i)-\frac{\partial S_{ik}(\boldsymbol{b}_i)}{\partial\boldsymbol{b}_i}\right\} - (1-\delta_i)\frac{\sum_{k=1}^K e^{-S_{ik}(\boldsymbol{b}_i)}\frac{\partial S_{ik}(\boldsymbol{b}_i)}{\partial\boldsymbol{b}_i}}{1-K+\sum_{k=1}^K e^{-S_{ik}(\boldsymbol{b}_i)}},$$

where $\frac{\partial S_{ik}(\boldsymbol{b}_i)}{\partial\boldsymbol{b}_i}=\int_0^{T_i}\alpha_k\boldsymbol{z}_i(s)e^{\boldsymbol{B}_k^\top(s)\boldsymbol{\psi}_k+\boldsymbol{\gamma}_k^\top\boldsymbol{w}_{ik}+\alpha_k m_i(s)}ds$. The information matrix is equal to

$$\mathcal{I}(\boldsymbol{b}_i) = (\boldsymbol{D}^{-1}+\omega\boldsymbol{Z}_i^\top\boldsymbol{Z}_i) + \sum_{k=1}^K\delta_{ik}\frac{\partial^2 S_{ik}(\boldsymbol{b}_i)}{\partial\boldsymbol{b}_i\boldsymbol{b}_i^\top} + (1-\delta_i)\frac{\partial}{\partial\boldsymbol{b}_i^\top}\left\{\frac{\sum_{k=1}^K e^{-S_{ik}(\boldsymbol{b}_i)}\frac{\partial S_{ik}(\boldsymbol{b}_i)}{\partial\boldsymbol{b}_i}}{1-K+\sum_{k=1}^K e^{-S_{ik}(\boldsymbol{b}_i)}}\right\},$$

where

$$\frac{\partial}{\partial\boldsymbol{b}_i^\top}\left\{\frac{\sum_{k=1}^K e^{-S_{ik}(\boldsymbol{b}_i)}\frac{\partial S_{ik}(\boldsymbol{b}_i)}{\partial\boldsymbol{b}_i}}{1-K+\sum_{k=1}^K e^{-S_{ik}(\boldsymbol{b}_i)}}\right\} = \boldsymbol{v}\boldsymbol{v}^\top + \left\{\sum_{k=1}^K e^{-S_{ik}(\boldsymbol{b}_i)}\frac{\partial^2 S_{ik}(\boldsymbol{b}_i)}{\partial\boldsymbol{b}_i\boldsymbol{b}_i^\top} - \sum_{k=1}^K e^{-S_{ik}(\boldsymbol{b}_i)}\frac{\partial S_{ik}(\boldsymbol{b}_i)}{\partial\boldsymbol{b}_i}\frac{\partial S_{ik}(\boldsymbol{b}_i)}{\partial\boldsymbol{b}_i^\top}\right\}\Bigg/\left\{1-K+\sum_{k=1}^K e^{-S_{ik}(\boldsymbol{b}_i)}\right\},$$

where $\boldsymbol{v} = \sum_{k=1}^{K} e^{-S_{ik}(\boldsymbol{b}_i)} \frac{\partial S_{ik}(\boldsymbol{b}_i)}{\partial \boldsymbol{b}_i} \Big/ \left\{1 - K + \sum_{k=1}^{K} e^{-S_{ik}(\boldsymbol{b}_i)}\right\}$ and

$$\frac{\partial^2 S_{ik}(\boldsymbol{b}_i)}{\partial \boldsymbol{b}_i \boldsymbol{b}_i^\top} = \int_0^{T_i} \alpha_k^2 \boldsymbol{z}_i(s) \boldsymbol{z}_i^\top(s) e^{\boldsymbol{B}_k^\top(s)\boldsymbol{\psi}_k + \boldsymbol{\gamma}_k^\top \boldsymbol{w}_{ik} + \alpha_k m_i(s)} ds.$$

The posterior distribution of the survival model parameters is proportional to

$$f(\boldsymbol{\theta}_s | \mathcal{D}, \boldsymbol{b}; \boldsymbol{\beta}) \propto \prod_{i=1}^{N} I\left[\sum_{k=1}^{K} F_{ik}^{M}\{T_i | M_i(T_i), \boldsymbol{w}_{ik}; \boldsymbol{\theta}_{sk}\} < 1\right]$$
$$\exp\left[\sum_{k=1}^{K} \delta_{ik} \left\{\boldsymbol{B}_k^\top(T_i)\boldsymbol{\psi}_k + \boldsymbol{\gamma}_k^\top \boldsymbol{w}_{ik} + \alpha_k m_i(T_i) - S_{ik}(\boldsymbol{\theta}_{sk})\right\}\right.$$
$$\left. + (1 - \delta_i) \log\left\{1 - K + \sum_{k=1}^{K} e^{-S_{ik}(\boldsymbol{\theta}_{sk})}\right\}\right] f(\boldsymbol{\theta}_s),$$

where $S_{ik}(\boldsymbol{\theta}_{sk}) = \int_0^{T_i} e^{\boldsymbol{B}_k^\top(s)\boldsymbol{\psi}_k + \boldsymbol{\gamma}_k^\top \boldsymbol{w}_{ik} + \alpha_k m_i(s)} ds$. The first-order partial derivatives are equal to

$$\frac{\partial \log f(\boldsymbol{\theta}_s | \mathcal{D}, \boldsymbol{b}; \boldsymbol{\beta})}{\partial \boldsymbol{\theta}_{sk}} = \frac{\partial \log f(\boldsymbol{\theta}_s)}{\partial \boldsymbol{\theta}_{sk}} + \sum_{i=1}^{N} \left[\delta_{ik} \left\{\begin{pmatrix} \boldsymbol{B}_k(T_i) \\ \boldsymbol{w}_{ik} \\ m_i(T_i) \end{pmatrix} - \partial S_{ik}(\boldsymbol{\theta}_{sk}) / \partial \boldsymbol{\theta}_{sk}\right\}\right.$$
$$\left. - (1 - \delta_i) \frac{e^{-S_{ik}(\boldsymbol{\theta}_{sk})} \partial S_{ik}(\boldsymbol{\theta}_{sk}) / \partial \boldsymbol{\theta}_{sk}}{1 - K + \sum_{k=1}^{K} e^{-S_{ik}(\boldsymbol{\theta}_{sk})}}\right],$$

where $\partial S_{ik}(\boldsymbol{\theta}_{sk}) / \partial \boldsymbol{\theta}_{sk} = \int_0^{T_i} \begin{pmatrix} \boldsymbol{B}_k(s) \\ \boldsymbol{w}_{ik} \\ m_i(s) \end{pmatrix} e^{\boldsymbol{B}_k^\top(s)\boldsymbol{\psi}_k + \boldsymbol{\gamma}_k^\top \boldsymbol{w}_{ik} + \alpha_k m_i(s)} ds.$

1.4 *Full conditional posterior distributions for the SPM-2 model*

The posterior distribution of the random effects under the SPM-2 model is proportional to

$$f(\boldsymbol{b}_i | \mathcal{D}; \boldsymbol{\theta}) \propto \exp\Bigg(-\frac{1}{2} \boldsymbol{b}_i^\top (\boldsymbol{D}^{-1} + \omega \boldsymbol{Z}_i^\top \boldsymbol{Z}_i) \boldsymbol{b}_i + \omega \boldsymbol{b}_i^\top \boldsymbol{Z}_i^\top (\boldsymbol{Y}_i - \boldsymbol{X}_i \boldsymbol{\beta})$$
$$- \sum_{k=1}^{K} \delta_{ik} \frac{1 + c_k}{c_k} \log\{1 + c_k S_{ik}(\boldsymbol{b}_i)\} + \sum_{k=1}^{K} \delta_{ik} \alpha_k \boldsymbol{b}_i^\top \boldsymbol{z}_i(T_i)$$
$$+ (1 - \delta_i) \log\left[1 - K + \sum_{k=1}^{K} \{1 + c_k S_{ik}(\boldsymbol{b}_i)\}^{-1/c_k}\right]\Bigg) \times I\left[\sum_{k=1}^{K} F_{ik}^{M}\{T_i | M_i(T_i), \boldsymbol{w}_{ik}; \boldsymbol{\theta}_{sk}\} < 1\right] \quad (1.4)$$

The corresponding score function of (1.4) is equal to

$$\mathcal{U}(\boldsymbol{b}_i) = -(\boldsymbol{D}^{-1} + \omega \boldsymbol{Z}_i^\top \boldsymbol{Z}_i)\boldsymbol{b}_i + \omega \boldsymbol{Z}_i^\top (\boldsymbol{Y}_i - \boldsymbol{X}_i\boldsymbol{\beta}) + \sum_{k=1}^{K} \left\{ -\delta_{ik} \frac{(1+c_k)\frac{\partial S_{ik}(\boldsymbol{b}_i)}{\partial \boldsymbol{b}_i}}{1 + c_k S_{ik}(\boldsymbol{b}_i)} + \delta_{ik}\alpha_k \boldsymbol{z}_i(T_i) \right\}$$
$$- (1-\delta_i) \frac{\sum_{k=1}^{K} \{1 + c_k S_{ik}(\boldsymbol{b}_i)\}^{-(1+c_k)/c_k} \frac{\partial S_{ik}(\boldsymbol{b}_i)}{\partial \boldsymbol{b}_i}}{1 - K + \sum_{k=1}^{K} \{1 + c_k S_{ik}(\boldsymbol{b}_i)\}^{-1/c_k}}.$$

The information matrix is equal to

$$\mathcal{I}(\boldsymbol{b}_i) = \boldsymbol{D}^{-1} + \omega \boldsymbol{Z}_i^\top \boldsymbol{Z}_i + \sum_{k=1}^{K} \delta_{ik}(1+c_k) \frac{\frac{\partial^2 S_{ik}(\boldsymbol{b}_i)}{\partial \boldsymbol{b}_i \partial \boldsymbol{b}_i^\top} \{1 + c_k S_{ik}(\boldsymbol{b}_i)\} - c_k \frac{\partial S_{ik}(\boldsymbol{b}_i)}{\partial \boldsymbol{b}_i} \frac{\partial S_{ik}(\boldsymbol{b}_i)}{\partial \boldsymbol{b}_i^\top}}{\{1 + c_k S_{ik}(\boldsymbol{b}_i)\}^2}$$
$$(1-\delta_i) \frac{\partial}{\partial \boldsymbol{b}_i^\top} \left[\frac{\sum_{k=1}^{K} \{1 + c_k S_{ik}(\boldsymbol{b}_i)\}^{-(1+c_k)/c_k} \frac{\partial S_{ik}(\boldsymbol{b}_i)}{\partial \boldsymbol{b}_i}}{1 - K + \sum_{k=1}^{K} \{1 + c_k S_{ik}(\boldsymbol{b}_i)\}^{-1/c_k}} \right],$$

where

$$\frac{\partial}{\partial \boldsymbol{b}_i^\top} \left[\frac{\sum_{k=1}^{K} \{1 + c_k S_{ik}(\boldsymbol{b}_i)\}^{-(1+c_k)/c_k} \frac{\partial S_{ik}(\boldsymbol{b}_i)}{\partial \boldsymbol{b}_i}}{1 - K + \sum_{k=1}^{K} \{1 + c_k S_{ik}(\boldsymbol{b}_i)\}^{-1/c_k}} \right]$$
$$= \frac{\partial}{\partial \boldsymbol{b}_i^\top} \left[\sum_{k=1}^{K} \{1 + c_k S_{ik}(\boldsymbol{b}_i)\}^{-(1+c_k)/c_k} \frac{\partial S_{ik}(\boldsymbol{b}_i)}{\partial \boldsymbol{b}_i} \right] \Bigg/ \left[1 - K + \sum_{k=1}^{K} \{1 + c_k S_{ik}(\boldsymbol{b}_i)\}^{-1/c_k} \right] + \boldsymbol{v}\boldsymbol{v}^\top$$

where $\boldsymbol{v} = \sum_{k=1}^{K} \{1 + c_k S_{ik}(\boldsymbol{b}_i)\}^{-(1+c_k)/c_k} \frac{\partial S_{ik}(\boldsymbol{b}_i)}{\partial \boldsymbol{b}_i} \Big/ \left[1 - K + \sum_{k=1}^{K} \{1 + c_k S_{ik}(\boldsymbol{b}_i)\}^{-1/c_k} \right]$. In addition,

$$\frac{\partial}{\partial \boldsymbol{b}_i^\top} \sum_{k=1}^{K} \{1 + c_k S_{ik}(\boldsymbol{b}_i)\}^{-(1+c_k)/c_k} \frac{\partial S_{ik}(\boldsymbol{b}_i)}{\partial \boldsymbol{b}_i} =$$
$$- \sum_{k=1}^{K} (1+c_k) \{1 + c_k S_{ik}(\boldsymbol{b}_i)\}^{-\frac{(1+c_k)}{c_k} - 1} \frac{\partial S_{ik}(\boldsymbol{b}_i)}{\partial \boldsymbol{b}_i} \frac{\partial S_{ik}(\boldsymbol{b}_i)}{\partial \boldsymbol{b}_i^\top}$$
$$+ \sum_{k=1}^{K} \{1 + c_k S_{ik}(\boldsymbol{b}_i)\}^{-(1+c_k)/c_k} \frac{\partial^2 S_{ik}(\boldsymbol{b}_i)}{\partial \boldsymbol{b}_i \partial \boldsymbol{b}_i^\top}.$$

The conditional posterior distribution of the survival model parameters is proportional to

$$f(\boldsymbol{\theta}_s | \mathcal{D}, \boldsymbol{b}; \boldsymbol{\beta}) \propto f(\boldsymbol{\theta}_s) \prod_{i=1}^{N} I \left[\sum_{k=1}^{K} F_{ik}^{M} \{T_i | M_i(T_i), \boldsymbol{w}_{ik}; \boldsymbol{\theta}_{sk}\} < 1 \right] \exp\left(\sum_{k=1}^{K} \left[-\delta_{ik} \frac{1+c_k}{c_k} \log\{1 + c_k S_{ik}(\boldsymbol{\theta}_{sk})\} \right.\right.$$
$$\left.\left. + \delta_{ik} \left\{ \boldsymbol{B}_k^\top(T_i)\boldsymbol{\psi}_k + \boldsymbol{\gamma}_k^\top \boldsymbol{w}_{ik} + \alpha_k m_i(T_i) \right\} \right] + (1-\delta_i) \log\left[1 - K + \sum_{k=1}^{K} \{1 + c_k S_{ik}(\boldsymbol{\theta}_{sk})\}^{-1/c_k} \right] \right),$$

with the score function being

$$\frac{\partial \log f(\boldsymbol{\theta}_s|\mathcal{D},\boldsymbol{b};\boldsymbol{\beta})}{\partial \boldsymbol{\theta}_{sk}} = \frac{\partial \log f(\boldsymbol{\theta}_s)}{\partial \boldsymbol{\theta}_{sk}} - \sum_{i=1}^{N} \delta_{ik} \frac{(1+c_k)\frac{\partial S_{ik}(\boldsymbol{\theta}_{sk})}{\partial \boldsymbol{\theta}_{sk}}}{1+c_k S_{ik}(\boldsymbol{\theta}_{sk})} + \sum_{i=1}^{N} \delta_{ik} \begin{pmatrix} \boldsymbol{B}_k(T_i) \\ \boldsymbol{w}_{ik} \\ m_i(T_i) \end{pmatrix}$$
$$- \sum_{i=1}^{N} (1-\delta_i) \frac{\{1+c_k S_{ik}(\boldsymbol{\theta}_{sk})\}^{-(1+c_k)/c_k} \frac{\partial S_{ik}(\boldsymbol{\theta}_{sk})}{\partial \boldsymbol{\theta}_{sk}}}{1-K+\sum_{k=1}^{K}\{1+c_k S_{ik}(\boldsymbol{\theta}_{sk})\}^{-1/c_k}}.$$

2. Predicting the "true" failure causes based on all available information

In this section, we prove equation (3.7) of the main manuscript.

$$\Pr\{K_i = k|\tilde{K}_i = j, T_i^\star = t, M_i(t), \boldsymbol{w}_i, \mathcal{D}_{misc,i}; \boldsymbol{\theta}, \boldsymbol{\theta}_{misc}\} = \frac{\Pr\{K_i = k, \tilde{K}_i = j|T_i^\star = t, M_i(t), \boldsymbol{w}_i, \mathcal{D}_{misc,i}; \boldsymbol{\theta}, \boldsymbol{\theta}_{misc}\}}{\Pr\{\tilde{K}_i = j|T_i^\star = t, M_i(t), \boldsymbol{w}_i, \mathcal{D}_{misc,i}; \boldsymbol{\theta}, \boldsymbol{\theta}_{misc}\}}$$
$$= \frac{\Pr\{K_i = k|T_i^\star = t, M_i(t), \boldsymbol{w}_i, \mathcal{D}_{misc,i}; \boldsymbol{\theta}, \boldsymbol{\theta}_{misc}\} \Pr\{\tilde{K}_i = j|K_i = k, T_i^\star = t, M_i(t), \boldsymbol{w}_i, \mathcal{D}_{misc,i}; \boldsymbol{\theta}, \boldsymbol{\theta}_{misc}\}}{\sum_{k=1}^{K} \Pr\{K_i = k|T_i^\star = t, M_i(t), \boldsymbol{w}_i, \mathcal{D}_{misc,i}; \boldsymbol{\theta}, \boldsymbol{\theta}_{misc}\} \Pr\{\tilde{K}_i = j|K_i = k, T_i^\star = t, M_i(t), \boldsymbol{w}_i, \mathcal{D}_{misc,i}; \boldsymbol{\theta}, \boldsymbol{\theta}_{misc}\}}$$
$$= \frac{\Pr\{K_i = k|T_i^\star = t, M_i(t), \boldsymbol{w}_i; \boldsymbol{\theta}_s\}\pi_{jk}(\mathcal{D}_{misc,i})}{\sum_{k=1}^{K} \Pr\{K_i = k|T_i^\star = t, M_i(t), \boldsymbol{w}_i; \boldsymbol{\theta}_s\}\pi_{jk}(\mathcal{D}_{misc,i})},$$

with the last results following from the model assumptions stating that (a) the failure cause probabilities do not depend on $\mathcal{D}_{misc,i}$ and $\boldsymbol{\theta}_{misc}$ and (b) the misclassification probabilities $\pi_{jk}(\mathcal{D}_{misc,i})$ are independent of the random effects and the parameters of interest, $\boldsymbol{\theta}$, thus independent of $M_i(t)$ and $\boldsymbol{w}_i$. It is well known (Beyersmann *and others*, 2011) that the failure cause probabilities conditionally on the survival time $T_i^\star = t$ are equal to

$$\Pr\{K_i = k|T_i^\star = t, M_i(t), \boldsymbol{w}_i; \boldsymbol{\theta}_s\} = \frac{\alpha_{ik}\{t|M_i(t), \boldsymbol{w}_i; \boldsymbol{\theta}_s\}}{\sum_{k=1}^{K} \alpha_{ik}\{t|M_i(t), \boldsymbol{w}_i; \boldsymbol{\theta}_s\}},$$

where $\alpha_{ik}\{t|M_i(t), \boldsymbol{w}_i; \boldsymbol{\theta}_s\}$ denotes the kth cause-specific hazard function for individual i. Based on the CIF definition, we know that $F_{ik}\{t|M_i(t), \boldsymbol{w}_{ik}; \boldsymbol{\theta}_{sk}\} = \int_0^t \alpha_{ik}\{u|M_i(u), \boldsymbol{w}_i; \boldsymbol{\theta}_s\} S_i\{u|M_i(u), \boldsymbol{w}_i; \boldsymbol{\theta}_s\} du$, which further implies that $f_{ik}\{t|M_i(t), \boldsymbol{w}_{ik}; \boldsymbol{\theta}_{sk}\} = \alpha_{ik}\{t|M_i(t), \boldsymbol{w}_i; \boldsymbol{\theta}_s\} S_i\{t|M_i(t), \boldsymbol{w}_i; \boldsymbol{\theta}_s\}$. Therefore,

$$\Pr\{K_i = k|T_i^\star = t, M_i(t), \boldsymbol{w}_i; \boldsymbol{\theta}_s\} = \frac{f_{ik}\{t|M_i(t), \boldsymbol{w}_{ik}; \boldsymbol{\theta}_{sk}\}}{\sum_{k=1}^{K} f_{ik}\{t|M_i(t), \boldsymbol{w}_{ik}; \boldsymbol{\theta}_{sk}\}}.$$

Then, it immediately follows that

$$\Pr\{K_i = k|\tilde{K}_i = j, T_i^\star = t, M_i(t), \boldsymbol{w}_i, \mathcal{D}_{misc,i}; \boldsymbol{\theta}, \boldsymbol{\theta}_{misc}\} = \frac{f_{ik}\{t|M_i(t), \boldsymbol{w}_{ik}; \boldsymbol{\theta}_{sk}\}\pi_{jk}(\mathcal{D}_{misc,i})}{\sum_{k=1}^{K} f_{ik}\{t|M_i(t), \boldsymbol{w}_{ik}; \boldsymbol{\theta}_{sk}\}\pi_{jk}(\mathcal{D}_{misc,i})}.$$

3. DETAILS ON THE MCMC ALGORITHM ABOUT THE MISCLASSIFICATION MODEL

We first outline the case where one does not intend to include covariate information in $\pi_{jk}(\mathcal{D}_{misc,i})$, i.e. $\mathcal{D}_{misc,i}$ is an empty set. Then, a natural choice for the prior distributions of the π_{jk}'s would be the independent Dirichlet$(a_{1k},\ldots,a_{Kk})$ distributions, $f(\pi_{1k},\ldots,\pi_{Kk}) \propto \prod_{j=1}^{K} \pi_{jk}^{a_{jk}-1}$, for $k = 1,\ldots,K$. It can be shown that, in this case, the conditional posterior distributions of $\pi_{1k},\ldots,\pi_{Kk}$ are independent Dirichlet$(a_{1k}+\sum_{i=1}^{N}\tilde{\delta}_{i1}\delta_{ik}, a_{2k}+\sum_{i=1}^{N}\tilde{\delta}_{i2}\delta_{ik},\ldots,a_{Kk}+\sum_{i=1}^{N}\tilde{\delta}_{iK}\delta_{ik})$, $k = 1,2,\ldots,K$. For $K=2$ competing risks, there are two distinct misclassification parameters. Assuming Beta(a_1,b_1) and Beta(a_2,b_2) prior distributions for π_{11} and π_{22}, respectively, results in the corresponding conditional posterior distributions being Beta$(a_1+\sum_{i=1}^{N}\tilde{\delta}_{i1}\delta_{i1}, b_1+\sum_{i=1}^{N}\tilde{\delta}_{i2}\delta_{i1})$ and Beta$(a_2+\sum_{i=1}^{N}\tilde{\delta}_{i2}\delta_{i2}, b_2+\sum_{i=1}^{N}\tilde{\delta}_{i1}\delta_{i2})$.

The conditional posterior distribution of the misclassification parameters is proportional to

$$f(\boldsymbol{\theta}_{misc}|\{(K_i,\mathcal{D}_{misc,i})\}_{i=1}^{N}) \propto f(\boldsymbol{\theta}_{misc}) \prod_{i=1}^{N}\prod_{j=1}^{K}\prod_{k=1}^{K} \pi_{jk}(\mathcal{D}_{misc,i})^{\tilde{\delta}_{ij}\delta_{ik}}.$$

Under two competing risks ($K=2$), we model the misclassification probabilities through logistic regression, i.e.

$$\Pr(\tilde{K}_i = 1|K_i,\mathcal{D}_{misc,i};\boldsymbol{\theta}_{misc}) = \frac{e^{\boldsymbol{X}_{misc,i}^{\top}\boldsymbol{\theta}_{misc}}}{1+e^{\boldsymbol{X}_{misc,i}^{\top}\boldsymbol{\theta}_{misc}}} = p_i, \tag{3.5}$$

where $\boldsymbol{X}_{misc,i}$ denotes the relevant design matrix associating K_i and $\mathcal{D}_{misc,i}$ with the probability of observing $\tilde{K}_i = 1$. Thus, $\Pr(\tilde{K}_i = 2|K_i,\mathcal{D}_{misc,i};\boldsymbol{\theta}_{misc}) = 1-p_i = \{1+e^{\boldsymbol{X}_{misc,i}^{\top}\boldsymbol{\theta}_{misc}}\}^{-1}$. Ignoring the prior distribution, the conditional posterior distribution of $\boldsymbol{\theta}_{misc}$ is proportional to $\prod_{i=1}^{N} p_i^{\tilde{\delta}_{i1}}(1-p_i)^{\tilde{\delta}_{i2}}$. The posterior distribution on the log scale is thus equal to

$$\log f(\boldsymbol{\theta}_{misc}|\{(K_i,\mathcal{D}_{misc,i})\}_{i=1}^{N}) = \sum_{i=1}^{N}\left\{\tilde{\delta}_{i1}\boldsymbol{X}_{misc,i}^{\top}\boldsymbol{\theta}_{misc} - \delta_i \log(1+e^{\boldsymbol{X}_{misc,i}^{\top}\boldsymbol{\theta}_{misc}})\right\}.$$

The score vector and the information matrix of $\boldsymbol{\theta}_{misc}$ are equal to $\mathcal{U}(\boldsymbol{\theta}_{misc}) = \sum_{i=1}^{N}\left\{\tilde{\delta}_{i1}\boldsymbol{X}_{misc,i} - \delta_i p_i \boldsymbol{X}_{misc,i}\right\}$ and $\mathcal{I}(\boldsymbol{\theta}_{misc}) = \sum_{i=1}^{N}\delta_i \boldsymbol{X}_{misc,i} p_i(1-p_i)\boldsymbol{X}_{misc,i}^{\top}$, respectively. To update the values of $\boldsymbol{\theta}_{misc}$, we used the method proposed by Gamerman (1997) by carrying out one Newton-Raphson step

starting at the current value and proposing a value from a multivariate Normal distribution with mean obtained from the Newton-Raphson step and covariance matrix equal to the inverse of the information matrix at the current value.

4. Comparison of model fit using the DIC criterion

To compare the fit of models SPM-1 and SPM-2, we use the marginalised version (Quintero and Lesaffre, 2018) of the deviance information criterion (DIC) proposed by Spiegelhalter *and others* (2002). To compute the marginal DIC criterion, we need to calculate the observed data likelihood, which requires integration over the random effects. Assuming that there is no misclassification, the observed data likelihood is equal to

$$f(\mathcal{D};\boldsymbol{\theta}) = \prod_{i=1}^{N} f(\boldsymbol{Y}_i, T_i, K_i|\boldsymbol{w}_i;\boldsymbol{\theta}) = \prod_{i=1}^{N} f(\boldsymbol{Y}_i;\boldsymbol{\theta}) \int f\{T_i, K_i|M_i(T_i), \boldsymbol{w}_i;\boldsymbol{\theta}_s\} f(\boldsymbol{b}_i|\boldsymbol{Y}_i;\boldsymbol{\theta}) d\boldsymbol{b}_i, \quad (4.6)$$

where we have actually factorized $f(\boldsymbol{Y}_i, T_i, K_i|\boldsymbol{w}_i;\boldsymbol{\theta})$ as $f(\boldsymbol{Y}_i;\boldsymbol{\theta})f(T_i, K_i|\boldsymbol{Y}_i, \boldsymbol{w}_i;\boldsymbol{\theta})$, with $f(T_i, K_i|\boldsymbol{Y}_i, \boldsymbol{w}_i;\boldsymbol{\theta})$ being equal to $\int f\{T_i, K_i|M_i(T_i), \boldsymbol{w}_i;\boldsymbol{\theta}_s\} f(\boldsymbol{b}_i|\boldsymbol{Y}_i;\boldsymbol{\theta}) d\boldsymbol{b}_i$, due to the conditional independence assumption for $\boldsymbol{Y}_i|\boldsymbol{b}_i$ and $T_i^\star|\boldsymbol{b}_i$. Also, it is straightforward to show that $\boldsymbol{Y}_i;\boldsymbol{\theta} \sim N(\boldsymbol{X}_i\boldsymbol{\beta}, \omega^{-1}\boldsymbol{I}_{n_i} + \boldsymbol{Z}_i\boldsymbol{D}\boldsymbol{Z}_i^\top)$ and $\boldsymbol{b}_i|\boldsymbol{Y}_i;\boldsymbol{\theta} \sim N(\boldsymbol{\mu}_{\boldsymbol{b}_i}, \boldsymbol{\Sigma}_{\boldsymbol{b}_i})$, where $\boldsymbol{\Sigma}_{\boldsymbol{b}_i} = (\boldsymbol{D}^{-1} + \omega\boldsymbol{Z}_i^\top\boldsymbol{Z}_i)^{-1}$ and $\boldsymbol{\mu}_{\boldsymbol{b}_i} = \boldsymbol{\Sigma}_{\boldsymbol{b}_i}\omega\boldsymbol{Z}_i^\top(\boldsymbol{Y}_i - \boldsymbol{X}_i\boldsymbol{\beta})$. Letting $Dev(\boldsymbol{\theta}) = -2\log f(\mathcal{D};\boldsymbol{\theta})$ be the deviance of the model, the DIC criterion is defined as DIC $= Dev\{E(\boldsymbol{\theta}|\mathcal{D})\} + 2p_{Eff}$, where $Dev\{E(\boldsymbol{\theta}|\mathcal{D})\}$ is the deviance evaluated at the posterior mean of the parameters and p_{Eff} is the effective number of parameters. Based on Spiegelhalter *and others* (2002), p_{Eff} should be equal to $p_{Eff} = E\{Dev(\boldsymbol{\theta})|\mathcal{D}\} - Dev\{E(\boldsymbol{\theta}|\mathcal{D})\}$, the posterior mean of the deviance minus the deviance at the posterior mean of the parameters. $E\{Dev(\boldsymbol{\theta})|\mathcal{D}\}$ can be approximated by the sample mean of the deviances evaluated at each value of the MCMC algorithm, while $Dev\{E(\boldsymbol{\theta}|\mathcal{D})\}$ can be approximated by the deviance evaluated at the mean of the MCMC sample.

In theory, the DIC would be straightforward to approximate based on the MCMC sample if the deviance was available in closed form, but the integral in (4.6) cannot be calcu-

lated analytically. Similarly to the pseudo-adaptive quadrature method proposed by Rizopoulos (2012), to consistently estimate $f(T_i, K_i|\boldsymbol{Y}_i, \boldsymbol{w}_i; \boldsymbol{\theta})$, we use Monte Carlo integration, i.e. $f(T_i, K_i|\boldsymbol{Y}_i, \boldsymbol{w}_i; \boldsymbol{\theta})$ is approximated by $N_{mc}^{-1} f\{T_i, K_i|M_i^{(j)}(t), \boldsymbol{w}_{ik}; \boldsymbol{\theta}_s\}$, where $m_i^{(j)}(t) = \boldsymbol{x}_i^\top(t)\boldsymbol{\beta} + \boldsymbol{z}_i^\top(t)\boldsymbol{b}_i^{(j)}$, $\boldsymbol{b}_i^{(j)} \sim N(\boldsymbol{\mu}_{\boldsymbol{b}_i}, \boldsymbol{\Sigma}_{\boldsymbol{b}_i})$, $j = 1, \ldots, N_{mc}$. Based on our experience, this Monte Carlo estimator had great performance as $\boldsymbol{\mu}_{\boldsymbol{b}_i}$ was very close to the mode of the integrand in (4.6) in almost all cases, a remark consistent with the literature (Rizopoulos, 2012). However, in very few cases, the model-based all-cause CIF may be greater than 1 when evaluated at $\boldsymbol{\mu}_{\boldsymbol{b}_i}$, leading to $f\{T_i, K_i|M_i(T_i), \boldsymbol{w}_i; \boldsymbol{\theta}_s\}$ being equal to zero. In these cases, the variance of the Monte Carlo estimator could be too large. To overcome this issue, we use importance sampling by exploiting the posterior sample of $\boldsymbol{b}_i$ available from MCMC. Specifically, we (a) simulate $\boldsymbol{b}_i^{(j)} \sim N(\bar{\boldsymbol{\mu}}_{\boldsymbol{b}_i}, \boldsymbol{\Sigma}_{\boldsymbol{b}_i})$, $j = 1, \ldots, N_{mc}$, where $\bar{\boldsymbol{\mu}}_{\boldsymbol{b}_i}$ is the marginal posterior mean of the random effect, $\boldsymbol{b}_i$, and (b) approximate $f(T_i, K_i|\boldsymbol{Y}_i, \boldsymbol{w}_i; \boldsymbol{\theta})$ by $N_{mc}^{-1} f\{T_i, K_i|M_i^{(j)}(t), \boldsymbol{w}_{ik}; \boldsymbol{\theta}_s\} f(\boldsymbol{b}_i^{(j)}|\boldsymbol{Y}_i; \boldsymbol{\theta})/g(\boldsymbol{b}_i^{(j)})$, where $g(\boldsymbol{b}_i^{(j)})$ is the density of the $N(\bar{\boldsymbol{\mu}}_{\boldsymbol{b}_i}, \boldsymbol{\Sigma}_{\boldsymbol{b}_i})$ distribution at $\boldsymbol{b}_i^{(j)}$ and $M_i^{(j)}(t) = \{m_i^{(j)}(s) : 0 \leqslant s \leqslant t\}$, with $m_i^{(j)}(t) = \boldsymbol{x}_i^\top(t)\boldsymbol{\beta} + \boldsymbol{z}_i^\top(t)\boldsymbol{b}_i^{(j)}$, $j = 1, \ldots, N_{mc}$. Since the deviance is calculated at each MCMC iteration, we cannot know beforehand if the importance sampling estimator would perform better than the simple Monte Carlo estimator. Thus, we employ the importance sampling estimator only if $\bar{\boldsymbol{\mu}}_{\boldsymbol{b}_i}$ leads to higher posterior density, $f(\boldsymbol{b}_i|\boldsymbol{Y}_i, T_i, K_i; \boldsymbol{\theta})$, than does $\boldsymbol{\mu}_{\boldsymbol{b}_i}$. Note that $f(\boldsymbol{b}_i|\boldsymbol{Y}_i, T_i, K_i; \boldsymbol{\theta})$ is proportional to the integrand in (4.6).

To evaluate the DIC criterion under failure cause misclassification, some minor adjustments are required. We use the same procedure but $f\{T_i, K_i|M_i(T_i), \boldsymbol{w}_i; \boldsymbol{\theta}_s\}$ in (4.6) is replaced by $f\{T_i, K_i|M_i(T_i), \boldsymbol{w}_i; \boldsymbol{\theta}_s\} \times \prod_{j=1}^K \prod_{k=1}^K \pi_{jk}(\mathcal{D}_{misc,i})^{\tilde{\delta}_{ij}\delta_{ik}}$ and $\sum_{j=1}^K \tilde{\delta}_{ij} \sum_{k=1}^K f_{ik}\{T_i|M_i(T_i), \boldsymbol{w}_{ik}; \boldsymbol{\theta}_{sk}\}\pi_{jk}(\mathcal{D}_{misc,i})$, for doubly and non-doubly sampled patients, respectively. For the right-censored individuals, nothing changed as $\Pr(\tilde{K}_i = 0|K_i = 0, \mathcal{D}_{misc}, \boldsymbol{\theta}_{misc}) = 1$.

5. CIF estimates conditional on observed marker states

As mentioned in the main manuscript, population-averaged CIFs conditional on the observed baseline marker states, i.e. $\Pr\{T_i^\star \leqslant t, K_i = k | y_i(0) \in S_g, \boldsymbol{w}_{ik}; \boldsymbol{\theta}\}$, can be of interest as well. In this section, we show that they can be estimated similarly to the procedure described in the main part of the manuscript. By standard calculations, it can be shown that $\Pr\{T_i^\star \leqslant t, K_i = k | y_i(0) \in S_g, \boldsymbol{w}_{ik}; \boldsymbol{\theta}\}$ is equal to

$$
\begin{aligned}
\Pr\{T_i^\star \leqslant t, K_i = k | y_i(0) \in S_g, \boldsymbol{w}_{ik}; \boldsymbol{\theta}\} &= \frac{\Pr\{T_i^\star \leqslant t, K_i = k, y_i(0) \in S_g | \boldsymbol{w}_{ik}; \boldsymbol{\theta}\}}{\Pr\{y_i(0) \in S_g; \boldsymbol{\theta}\}} \\
= \int\limits_{y_i(0) \in S_g} \int_0^t \frac{f\{u, k, y_i(0) | \boldsymbol{w}_{ik}; \boldsymbol{\theta}\}}{\Pr\{y_i(0) \in S_g; \boldsymbol{\theta}\}} \, du \, dy_i(0) & \\
= \int\limits_{y_i(0) \in S_g} \int_0^t \int \frac{f\{u, k, y_i(0) | \boldsymbol{b}_i, \boldsymbol{w}_{ik}; \boldsymbol{\theta}\} f(\boldsymbol{b}_i; \boldsymbol{\theta})}{\Pr\{y_i(0) \in S_g; \boldsymbol{\theta}\}} \, d\boldsymbol{b}_i \, du \, dy_i(0). & \qquad (5.7)
\end{aligned}
$$

Due to the model assumption of conditional independence between the marker and survival process given the random effects, (5.7) is equal to

$$
\begin{aligned}
& \int\limits_{y_i(0) \in S_g} \int \left[\int_0^t f(u, k | \boldsymbol{b}_i, \boldsymbol{w}_{ik}; \boldsymbol{\theta}) \, du \right] \frac{f\{y_i(0) | \boldsymbol{b}_i; \boldsymbol{\theta}\} f(\boldsymbol{b}_i; \boldsymbol{\theta})}{\Pr\{y_i(0) \in S_g; \boldsymbol{\theta}\}} \, d\boldsymbol{b}_i \, dy_i(0) \\
= & \int\limits_{y_i(0) \in S_g} \int F_{ik}\{t | M_i(t), \boldsymbol{w}_{ik}; \boldsymbol{\theta}_{sk}\} \frac{f\{y_i(0), \boldsymbol{b}_i; \boldsymbol{\theta}\}}{\Pr\{y_i(0) \in S_g; \boldsymbol{\theta}\}} \, d\boldsymbol{b}_i \, dy_i(0) \qquad (5.8)
\end{aligned}
$$

Thus, (5.8) can be estimated by drawing samples $\{y_{ig}^{(j)}(0), \boldsymbol{b}_{ig}^{(j)}\}_{j=1}^{N\text{mc}}$ for $\{y_i(0), \boldsymbol{b}_i\}$ from the

$$
N \left\{ \begin{pmatrix} \boldsymbol{x}_i^\top(0)\boldsymbol{\beta} \\ \boldsymbol{0} \end{pmatrix}, \begin{pmatrix} \sigma^2 + \boldsymbol{z}_i^\top(0)\boldsymbol{D}\boldsymbol{z}_i(0) & \boldsymbol{z}_i^\top(0)\boldsymbol{D} \\ \boldsymbol{D}\boldsymbol{z}_i(0) & \boldsymbol{D} \end{pmatrix} \right\},
$$

distribution, constrained such that $y_i(0) \in S_g$, i.e. $\Pr\{T_i^\star \leqslant t, K_i = k | y_i(0) \in S_g, \boldsymbol{w}_{ik}; \boldsymbol{\theta}\}$ can be approximated by $N_{\text{mc}}^{-1} \sum_{j=1}^{N\text{mc}} F_{ik}\{t | M_{ig}^{(j)}(t), \boldsymbol{w}_{ik}; \boldsymbol{\theta}_{sk}\}$, where $m_{ig}^{(j)}(t) = \boldsymbol{x}_i^\top(t)\boldsymbol{\beta} + \boldsymbol{z}_i^\top(t)\boldsymbol{b}_{ig}^{(j)}$ and $M_{ig}^{(j)}(t) = \{m_{ig}^{(j)}(s) : 0 \leqslant s \leqslant t\}$.

Population-averaged CIFs, conditional on being in certain observed marker states up to s and given survival up to s, e.g. $\Pr\{T_i^\star \leqslant t, K_i = k | T_i^\star > s, y_i(0) \in S_g, y_i(s) \in S_h, \boldsymbol{w}_i; \boldsymbol{\theta}\}$, can be estimated in a similar way. Specifically,

$$
\begin{aligned}
&\frac{\Pr\{s < T_i^\star \leqslant t, K_i = k, y_i(0) \in S_g, y_i(s) \in S_h | \boldsymbol{w}_i; \boldsymbol{\theta}\}}{\Pr\{s < T_i^\star, y_i(0) \in S_g, y_i(s) \in S_h | \boldsymbol{w}_i; \boldsymbol{\theta}\}} \\
&= \frac{\int\limits_{y_i(0)\in S_g} \int\limits_{y_i(s)\in S_h} \int_s^t f\{u, k, y_i(0), y_i(s) | \boldsymbol{w}_i; \boldsymbol{\theta}\}\, du\, dy_i(s)\, dy_i(0)}{\int\limits_{y_i(0)\in S_g} \int\limits_{y_i(s)\in S_h} \int_s^\infty f\{u, y_i(0), y_i(s) | \boldsymbol{w}_i; \boldsymbol{\theta}\}\, du\, dy_i(s)\, dy_i(0)} \\
&= \frac{\int\limits_{y_i(0)\in S_g} \int\limits_{y_i(s)\in S_h} \int_s^t \int f\{u, k, y_i(0), y_i(s) | \boldsymbol{b}_i, \boldsymbol{w}_i; \boldsymbol{\theta}\} f(\boldsymbol{b}_i; \boldsymbol{\theta})\, d\boldsymbol{b}_i\, du\, dy_i(s)\, dy_i(0)}{\int\limits_{y_i(0)\in S_g} \int\limits_{y_i(s)\in S_h} \int_s^\infty \int f\{u, y_i(0), y_i(s) | \boldsymbol{b}_i, \boldsymbol{w}_i; \boldsymbol{\theta}\} f(\boldsymbol{b}_i; \boldsymbol{\theta})\, d\boldsymbol{b}_i\, du\, dy_i(s)\, dy_i(0)}
\end{aligned}
\tag{5.9}
$$

By the conditional independence assumption of the proposed model, i.e. the marker and survival processes are independent given the random effects, it follows that (5.9) is equal to

$$
\begin{aligned}
&\frac{\int\limits_{y_i(0)\in S_g} \int\limits_{y_i(s)\in S_h} \int_s^t \int f(u, k | \boldsymbol{b}_i, \boldsymbol{w}_i; \boldsymbol{\theta}) f\{y_i(0), y_i(s) | \boldsymbol{b}_i; \boldsymbol{\theta}\} f(\boldsymbol{b}_i; \boldsymbol{\theta})\, d\boldsymbol{b}_i\, du\, dy_i(s)\, dy_i(0)}{\int\limits_{y_i(0)\in S_g} \int\limits_{y_i(s)\in S_h} \int_s^\infty \int f(u | \boldsymbol{b}_i, \boldsymbol{w}_i; \boldsymbol{\theta}) f\{y_i(0), y_i(s) | \boldsymbol{b}_i; \boldsymbol{\theta}\} f(\boldsymbol{b}_i; \boldsymbol{\theta})\, d\boldsymbol{b}_i\, du\, dy_i(s)\, dy_i(0)} \\
&= \frac{\int\limits_{y_i(0)\in S_g} \int\limits_{y_i(s)\in S_h} \int \left[\int_s^t f(u, k | \boldsymbol{b}_i, \boldsymbol{w}_i; \boldsymbol{\theta})\, du\right] f\{y_i(0), y_i(s) | \boldsymbol{b}_i; \boldsymbol{\theta}\} f(\boldsymbol{b}_i; \boldsymbol{\theta})\, d\boldsymbol{b}_i\, dy_i(s)\, dy_i(0)}{\int\limits_{y_i(0)\in S_g} \int\limits_{y_i(s)\in S_h} \int \left[\int_s^\infty f(u | \boldsymbol{b}_i, \boldsymbol{w}_i; \boldsymbol{\theta})\, du\right] f\{y_i(0), y_i(s) | \boldsymbol{b}_i; \boldsymbol{\theta}\} f(\boldsymbol{b}_i; \boldsymbol{\theta})\, d\boldsymbol{b}_i\, dy_i(s)\, dy_i(0)} \\
&= \frac{\int\limits_{y_i(0)\in S_g} \int\limits_{y_i(s)\in S_h} \int [F_{ik}\{t | M_i(t), \boldsymbol{w}_i; \boldsymbol{\theta}_{sk}\} - F_{ik}\{s | M_i(s), \boldsymbol{w}_i; \boldsymbol{\theta}_{sk}\}] \frac{f\{y_i(0), y_i(s), \boldsymbol{b}_i; \boldsymbol{\theta}\}}{\Pr\{y_i(0)\in S_g, y_i(s)\in S_h; \boldsymbol{\theta}\}}\, d\boldsymbol{b}_i\, dy_i(s)\, dy_i(0)}{\int\limits_{y_i(0)\in S_g} \int\limits_{y_i(s)\in S_h} \int S_i\{s | M_i(s), \boldsymbol{w}_i; \boldsymbol{\theta}_s\} \frac{f\{y_i(0), y_i(s), \boldsymbol{b}_i; \boldsymbol{\theta}\}}{\Pr\{y_i(0)\in S_g, y_i(s)\in S_h; \boldsymbol{\theta}\}}\, d\boldsymbol{b}_i\, dy_i(s)\, dy_i(0)}
\end{aligned}
$$

Thus, in a very similar way, $\Pr\{T_i^\star \leqslant t, K_i = k | T_i^\star > s, y_i(0) \in S_g, y_i(s) \in S_h, \boldsymbol{w}_i; \boldsymbol{\theta}\}$ can be estimated by drawing samples $\{y_{igh}^{(j)}(0), y_{igh}^{(j)}(s), \boldsymbol{b}_{igh}^{(j)}\}_{j=1}^{N\text{mc}}$ for $\{y_i(0), y_i(s), \boldsymbol{b}_i\}$ from the

$$
N\left\{\begin{pmatrix} \boldsymbol{x}_i^\top(0)\boldsymbol{\beta} \\ \boldsymbol{x}_i^\top(s)\boldsymbol{\beta} \\ \boldsymbol{0} \end{pmatrix}, \begin{pmatrix} \sigma^2 + \boldsymbol{z}_i^\top(0)\boldsymbol{D}\boldsymbol{z}_i(0) & \boldsymbol{z}_i^\top(0)\boldsymbol{D}\boldsymbol{z}_i(s) & \boldsymbol{z}_i(0)^\top\boldsymbol{D} \\ \boldsymbol{z}_i^\top(0)\boldsymbol{D}\boldsymbol{z}_i(s) & \sigma^2 + \boldsymbol{z}_i^\top(s)\boldsymbol{D}\boldsymbol{z}_i(s) & \boldsymbol{z}_i(s)^\top\boldsymbol{D} \\ \boldsymbol{D}\boldsymbol{z}_i(0) & \boldsymbol{D}\boldsymbol{z}_i(s) & \boldsymbol{D} \end{pmatrix}\right\},
$$

distribution, constrained such that $y_i(0) \in S_g$ and $y_i(s) \in S_h$, i.e. $\Pr\{T_i^\star \leqslant t, K_i = k | T_i^\star > s, y_i(0) \in S_g, y_i(s) \in S_h, \boldsymbol{w}_i; \boldsymbol{\theta}\}$ can be approximated by

$$
\frac{\sum_{j=1}^{N\text{mc}} \left[F_{ik}\{t | M_{igh}^{(j)}(t), \boldsymbol{w}_{ik}; \boldsymbol{\theta}_{sk}\} - F_{ik}\{s | M_{igh}^{(j)}(s), \boldsymbol{w}_{ik}; \boldsymbol{\theta}_{sk}\}\right]}{\sum_{j=1}^{N\text{mc}} S_i\{s | M_{igh}^{(j)}(s), \boldsymbol{w}_i; \boldsymbol{\theta}_s\}},
$$

where $m_{igh}^{(j)}(t) = \boldsymbol{x}_i^\top(t)\boldsymbol{\beta} + \boldsymbol{z}_i^\top(t)\boldsymbol{b}_{igh}^{(j)}$ and $M_{igh}^{(j)}(t) = \{m_{igh}^{(j)}(s) : 0 \leqslant s \leqslant t\}$.

6. Simulation study details

The true parameter values used in the simulation study described in Section 5 of the main part of the manuscript were $\boldsymbol{\beta}^\top = (12.85, 6.03, 0.77, 0)$, $\text{vech}(\boldsymbol{D})^\top = (25.09, -5.08, -2.65, 10.18, 1.09, 0.85)$, and $\omega = 1/8.14$, where vech stands for the "vector-half" operator stacking the columns of the lower triangular part of a symmetric matrix. We assumed that $\alpha_1 = -0.16$, $\alpha_2 = -0.02$, $\gamma_1 = 0.15$, and $\gamma_2 = -0.15$. For the baseline CIF levels, we assumed that

$$
\begin{aligned}
u_{11}(t) &= 0.62 \times 0.25 \frac{\exp(-0.25t + 7 \times 0.16)}{1 - 0.62\{1 - \exp(-0.25t)\}} \\
u_{21}(t) &= 0.70 \times 0.13 \frac{\exp(-0.13t + 7 \times 0.02)}{1 - 0.70\{1 - \exp(-0.13t)\}} \\
u_{12}(t) &= 0.67 \times 0.25 \frac{\exp(-0.25t + 7 \times 0.16)}{1 - 0.67\{1 - \exp(-0.25t)\}} \\
u_{22}(t) &= 0.83 \times 0.13 \frac{\exp(-0.13t + 7 \times 0.02)}{1 - 0.83\{1 - \exp(-0.13t)\}}.
\end{aligned}
$$

The marker model was correctly specified in the fitted models. The B-splines matrices $\boldsymbol{B}_k(t)$, approximating the baseline CIF levels, had 2 and 3 knots for the first and the second failure type, respectively, placed at the observed quantiles of the respective event times. To derive inference on the model parameters, we applied the MCMC algorithm for fully observed failure causes using 200 draws as an burn-in period and recorded additional 10500 iterations. To account for the autocorrelation in the MCMC sample we thinned the chain by keeping every third draw, thus producing posterior inferences based on 3500 draws. Trace plots of posterior samples from the SPM-1 model applied to one simulated dataset are provided in Figure S1 and Figure S2. It can be seen that our algorithm mixes well and converges soon, although there is some small degree of auto-correlation in the posterior samples. Moreover, the Gelman-Rubin convergence diagnostic did not suggest convergence problems.

For the population parameters and the survival parameters, $\boldsymbol{\beta}$ and $\boldsymbol{\theta}_s$, respectively, we assumed independent normal distributions with zero means and variances of 100, for the covariance

matrix of the random effects, $\boldsymbol{D}$, the Inverse-Wishart $IW(\boldsymbol{A}, df)$ distribution with $df = 3$ and $\boldsymbol{A} = 3\times\text{diag}(25, 5, 5)$, and for the within-subject precision, ω, the Gamma$(0.01, 0.01)$ distribution.

To estimate the population CIFs and latent marker state probabilities, the numerical integration required was performed through $N_{mc} = 1000$ Monte Carlo draws using a posterior sample for $\boldsymbol{\theta}$ of $L = 350$ draws obtained by keeping every tenth MCMC draw. Parameter estimates were based on the posterior medians, with the corresponding credible intervals estimated by the observed 2.5% and 97.5% quantile of the MCMC posterior sample. For each fitted model, we also calculated the marginal DIC criterion using $N_{mc} = 200$ based on 500 draws from the posterior distribution of $\boldsymbol{\theta}$ obtained by keeping every seventh draw.

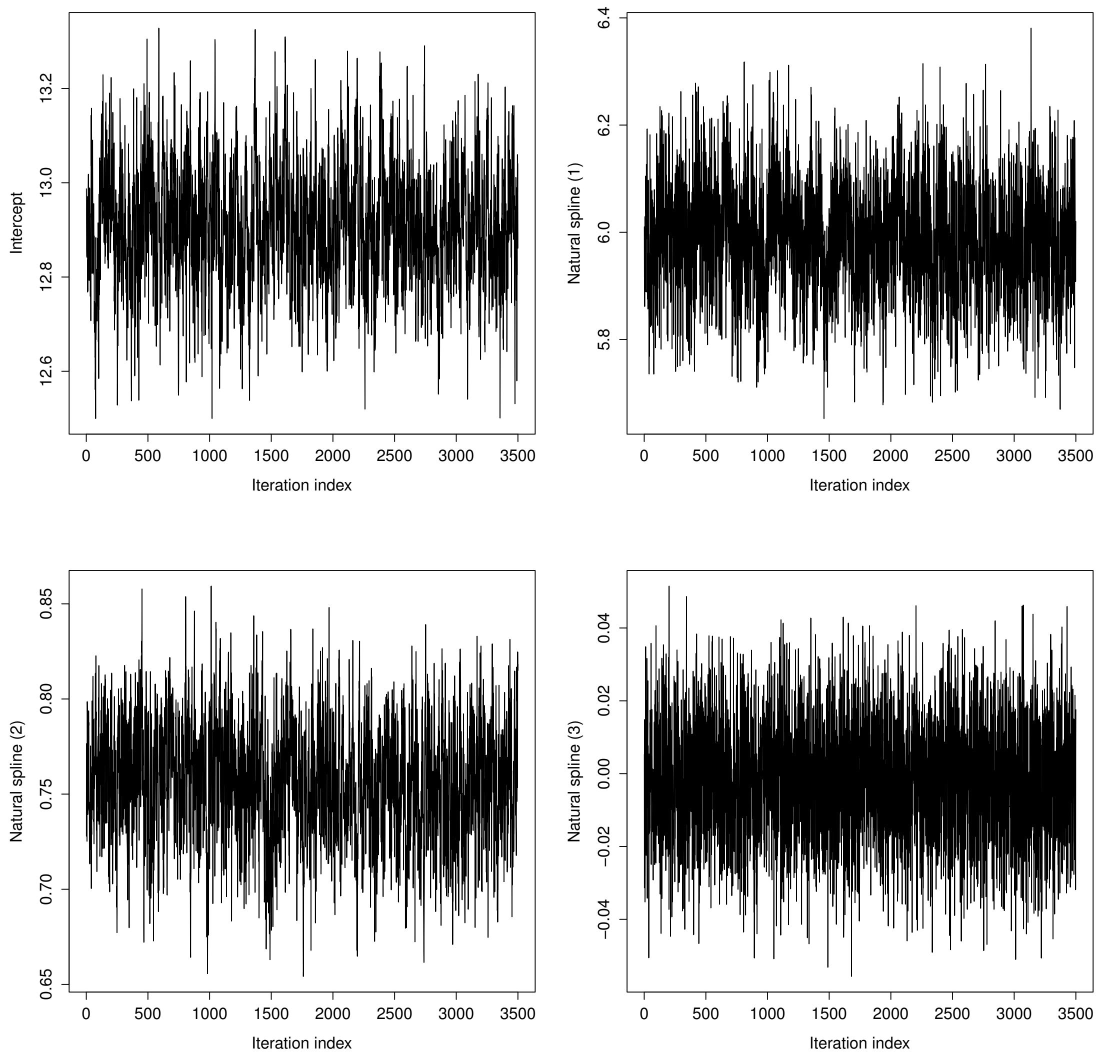

Figure S1. Trace plots of posterior samples for the longitudinal model parameters (i.e. intercept and 3 parameters associated with the natural cubic splines) from the SPM-1 model.

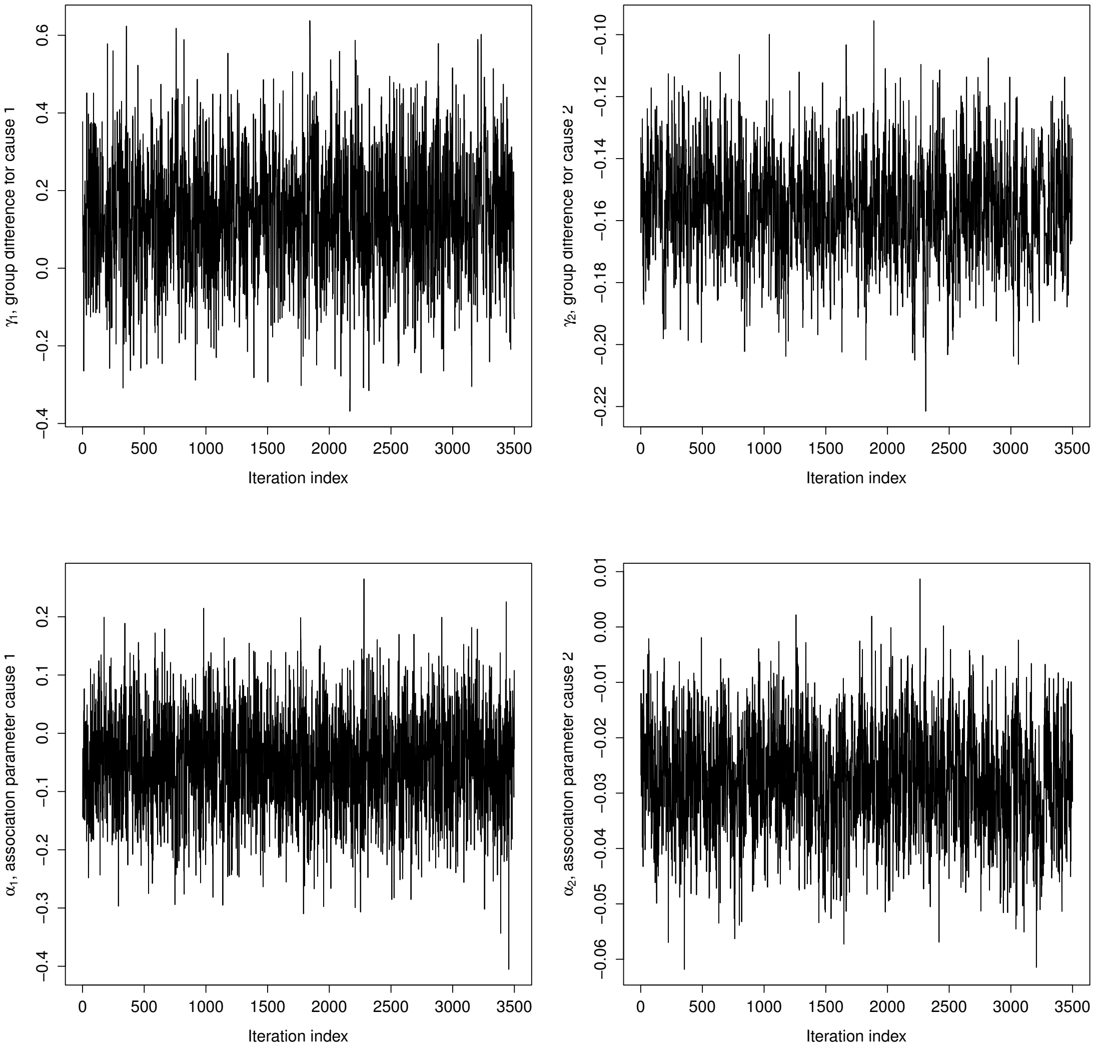

Figure S2. Trace plots of posterior samples for the survival model parameters (differences between the two groups for cause 1 and 2, and association parameters for cause 1 and 2) from the SPM-1 model.

7. Additional results for the Simulation studies of Section 5 of the main manuscript

In this section, we present additional results for the simulation studies described in Section 5 of the main part of the manuscript. In the main simulation study (subsections 7.1-7.3), the true failure cause is assumed to be known in a small subset of the population (double sampling) and the double sample information is taken into account in the SPM-1 and SPM-2 models.

7.1 *Simulation study results for latent marker state probabilities*

In this subsection, we present results for latent marker state probabilities (Tables S1-S4). Both the SPM-1 and SPM-2 model, even when misspecified, yielded estimates with small biases and adequate coverage rates (90.2-96.4%).

Table S1. Simulation study results for latent marker state probabilities (%) for group 1 ($w = 1$) when the data are simulated by SPM-1 and there is misclassification‡.

State	Est	$t=0$	$t=2$	$t=4$	$t=6$	$t=8$	$t=10$	$t=0$	$t=2$	$t=4$	$t=6$	$t=8$	$t=10$
		Results from SPM-1						Results from SPM-2					
$\{m_i(t) < \sqrt{50}\} \cap T_i^\star > t$	True	12.431	0.183	0.037	0.020	0.005	0.001	12.431	0.183	0.037	0.020	0.005	0.001
$\{m_i(t) < \sqrt{50}\} \cap T_i^\star > t$	Median	12.425	0.183	0.040	0.023	0.007	0.002	12.421	0.185	0.043	0.025	0.008	0.002
$\{m_i(t) < \sqrt{50}\} \cap T_i^\star > t$	Bias	-0.006	-0.000	0.003	0.003	0.002	0.001	-0.010	0.001	0.006	0.005	0.003	0.001
$\{m_i(t) < \sqrt{50}\} \cap T_i^\star > t$	Coverage	95.400	94.000	93.200	93.800	93.000	92.600	95.000	95.200	91.400	91.800	90.200	93.200
$\{\sqrt{50} \leqslant m_i(t) < \sqrt{100}\} \cap T_i^\star > t$	True	16.038	1.007	0.307	0.190	0.103	0.050	16.038	1.007	0.307	0.190	0.103	0.050
$\{\sqrt{50} \leqslant m_i(t) < \sqrt{100}\} \cap T_i^\star > t$	Median	16.005	1.007	0.318	0.203	0.113	0.060	16.008	0.994	0.321	0.206	0.118	0.063
$\{\sqrt{50} \leqslant m_i(t) < \sqrt{100}\} \cap T_i^\star > t$	Bias	-0.033	-0.000	0.011	0.013	0.010	0.009	-0.030	-0.013	0.013	0.016	0.015	0.013
$\{\sqrt{50} \leqslant m_i(t) < \sqrt{100}\} \cap T_i^\star > t$	Coverage	94.000	94.200	91.800	93.600	92.600	91.600	93.800	94.600	92.600	93.000	91.600	91.800
$\{\sqrt{100} \leqslant m_i(t) < \sqrt{200}\} \cap T_i^\star > t$	True	31.710	7.103	3.098	2.040	1.511	1.127	31.710	7.103	3.098	2.040	1.511	1.127
$\{\sqrt{100} \leqslant m_i(t) < \sqrt{200}\} \cap T_i^\star > t$	Median	31.671	7.097	3.142	2.100	1.564	1.182	31.680	7.039	3.120	2.084	1.554	1.178
$\{\sqrt{100} \leqslant m_i(t) < \sqrt{200}\} \cap T_i^\star > t$	Bias	-0.039	-0.006	0.044	0.060	0.053	0.055	-0.030	-0.064	0.022	0.044	0.043	0.051
$\{\sqrt{100} \leqslant m_i(t) < \sqrt{200}\} \cap T_i^\star > t$	Coverage	94.000	94.400	92.000	93.200	92.200	93.000	93.400	95.000	93.800	94.400	93.200	92.800
$\{\sqrt{200} \leqslant m_i(t) < \sqrt{250}\} \cap T_i^\star > t$	True	12.103	6.468	3.468	2.364	1.909	1.569	12.103	6.468	3.468	2.364	1.909	1.569
$\{\sqrt{200} \leqslant m_i(t) < \sqrt{250}\} \cap T_i^\star > t$	Median	12.096	6.461	3.488	2.397	1.942	1.604	12.099	6.438	3.464	2.376	1.920	1.585
$\{\sqrt{200} \leqslant m_i(t) < \sqrt{250}\} \cap T_i^\star > t$	Bias	-0.007	-0.007	0.020	0.033	0.033	0.036	-0.004	-0.030	-0.003	0.011	0.011	0.016
$\{\sqrt{200} \leqslant m_i(t) < \sqrt{250}\} \cap T_i^\star > t$	Coverage	95.600	94.600	94.200	93.400	93.200	93.600	95.800	94.800	94.400	94.800	93.600	93.800
$\{\sqrt{250} \leqslant m_i(t) < \sqrt{350}\} \cap T_i^\star > t$	True	15.610	16.626	10.745	7.677	6.502	5.607	15.610	16.626	10.745	7.677	6.502	5.607
$\{\sqrt{250} \leqslant m_i(t) < \sqrt{350}\} \cap T_i^\star > t$	Median	15.613	16.608	10.756	7.711	6.542	5.656	15.612	16.604	10.716	7.665	6.485	5.595
$\{\sqrt{250} \leqslant m_i(t) < \sqrt{350}\} \cap T_i^\star > t$	Bias	0.003	-0.018	0.011	0.034	0.040	0.049	0.002	-0.022	-0.029	-0.013	-0.017	-0.012
$\{\sqrt{250} \leqslant m_i(t) < \sqrt{350}\} \cap T_i^\star > t$	Coverage	94.000	94.000	94.800	94.200	94.200	94.200	93.800	94.200	94.200	94.600	95.200	94.600
$\{\sqrt{350} \leqslant m_i(t) < \sqrt{500}\} \cap T_i^\star > t$	True	9.229	24.230	20.064	15.723	13.847	12.389	9.229	24.230	20.064	15.723	13.847	12.389
$\{\sqrt{350} \leqslant m_i(t) < \sqrt{500}\} \cap T_i^\star > t$	Median	9.251	24.211	20.021	15.686	13.823	12.374	9.246	24.268	20.019	15.652	13.762	12.294
$\{\sqrt{350} \leqslant m_i(t) < \sqrt{500}\} \cap T_i^\star > t$	Bias	0.022	-0.019	-0.043	-0.037	-0.025	-0.014	0.017	0.038	-0.045	-0.070	-0.085	-0.095
$\{\sqrt{350} \leqslant m_i(t) < \sqrt{500}\} \cap T_i^\star > t$	Coverage	93.800	94.200	93.800	94.200	95.000	95.000	93.600	94.600	93.600	93.800	94.800	93.600
$\{\sqrt{500} \leqslant m_i(t)\} \cap T_i^\star > t$	True	2.880	24.682	30.397	31.163	28.411	26.223	2.880	24.682	30.397	31.163	28.411	26.223
$\{\sqrt{500} \leqslant m_i(t)\} \cap T_i^\star > t$	Median	2.909	24.737	30.488	31.265	28.496	26.286	2.904	24.832	30.595	31.334	28.510	26.233
$\{\sqrt{500} \leqslant m_i(t)\} \cap T_i^\star > t$	Bias	0.029	0.055	0.091	0.101	0.084	0.064	0.024	0.150	0.198	0.171	0.098	0.010
$\{\sqrt{500} \leqslant m_i(t)\} \cap T_i^\star > t$	Coverage	92.800	93.400	92.000	92.400	93.400	93.800	92.600	92.600	92.400	92.600	92.600	93.200

† Results from 500 replications with each dataset including 2000 individuals. The "true" marker evolution was based on linear splines with knots at 1 and 5 years since baseline and it was correctly specified in the fitted SPM-1 and SPM-2 models. "True" denotes the true parameter values; "Median" the mean of posterior medians over the 500 replications; "Bias" the mean bias for posterior median estimates; and "Coverage" the empirical coverage probability (%) of posterior credible intervals.

Table S2. Simulation study results for latent marker state probabilities (%) for group 0 ($w = 0$) when the data are simulated by SPM-1 and there is misclassification†.

State	Est	$t=0$	$t=2$	$t=4$	$t=6$	$t=8$	$t=10$	$t=0$	$t=2$	$t=4$	$t=6$	$t=8$	$t=10$
		Results from SPM-1						Results from SPM-2					
$\{m_i(t) < \sqrt{50}\} \cap T_i^\star > t$	True	12.431	0.193	0.039	0.021	0.005	0.001	12.431	0.193	0.039	0.021	0.005	0.001
$\{m_i(t) < \sqrt{50}\} \cap T_i^\star > t$	Median	12.425	0.192	0.042	0.024	0.007	0.002	12.421	0.190	0.043	0.024	0.007	0.002
$\{m_i(t) < \sqrt{50}\} \cap T_i^\star > t$	Bias	-0.006	-0.001	0.002	0.003	0.002	0.001	-0.010	-0.003	0.004	0.003	0.002	0.001
$\{m_i(t) < \sqrt{50}\} \cap T_i^\star > t$	Coverage	95.400	94.800	94.200	94.200	92.000	92.400	95.000	95.000	93.000	92.600	92.600	93.400
$\{\sqrt{50} \leqslant m_i(t) < \sqrt{100}\} \cap T_i^\star > t$	True	16.038	1.031	0.315	0.193	0.103	0.049	16.038	1.031	0.315	0.193	0.103	0.049
$\{\sqrt{50} \leqslant m_i(t) < \sqrt{100}\} \cap T_i^\star > t$	Median	16.005	1.028	0.324	0.203	0.111	0.057	16.008	1.007	0.320	0.202	0.113	0.059
$\{\sqrt{50} \leqslant m_i(t) < \sqrt{100}\} \cap T_i^\star > t$	Bias	-0.033	-0.003	0.008	0.011	0.008	0.008	-0.030	-0.023	0.005	0.009	0.010	0.010
$\{\sqrt{50} \leqslant m_i(t) < \sqrt{100}\} \cap T_i^\star > t$	Coverage	94.000	94.600	94.200	94.600	93.600	92.400	93.800	94.800	93.800	94.000	92.200	92.200
$\{\sqrt{100} \leqslant m_i(t) < \sqrt{200}\} \cap T_i^\star > t$	True	31.710	7.136	3.098	2.016	1.474	1.081	31.710	7.136	3.098	2.016	1.474	1.081
$\{\sqrt{100} \leqslant m_i(t) < \sqrt{200}\} \cap T_i^\star > t$	Median	31.671	7.119	3.130	2.062	1.512	1.120	31.680	7.038	3.085	2.033	1.498	1.120
$\{\sqrt{100} \leqslant m_i(t) < \sqrt{200}\} \cap T_i^\star > t$	Bias	-0.039	-0.017	0.032	0.047	0.039	0.039	-0.030	-0.098	-0.013	0.018	0.024	0.039
$\{\sqrt{100} \leqslant m_i(t) < \sqrt{200}\} \cap T_i^\star > t$	Coverage	94.000	95.000	94.400	94.800	95.200	93.000	93.400	94.200	95.200	96.400	94.800	94.200
$\{\sqrt{200} \leqslant m_i(t) < \sqrt{250}\} \cap T_i^\star > t$	True	12.103	6.450	3.433	2.312	1.844	1.493	12.103	6.450	3.433	2.312	1.844	1.493
$\{\sqrt{200} \leqslant m_i(t) < \sqrt{250}\} \cap T_i^\star > t$	Median	12.096	6.435	3.443	2.335	1.864	1.515	12.099	6.396	3.406	2.308	1.845	1.507
$\{\sqrt{200} \leqslant m_i(t) < \sqrt{250}\} \cap T_i^\star > t$	Bias	-0.007	-0.014	0.011	0.022	0.020	0.022	-0.004	-0.054	-0.027	-0.005	0.002	0.014
$\{\sqrt{200} \leqslant m_i(t) < \sqrt{250}\} \cap T_i^\star > t$	Coverage	95.600	95.800	96.200	95.000	95.000	93.000	95.800	94.600	95.400	96.000	96.000	94.200
$\{\sqrt{250} \leqslant m_i(t) < \sqrt{350}\} \cap T_i^\star > t$	True	15.610	16.505	10.570	7.462	6.238	5.310	15.610	16.505	10.570	7.462	6.238	5.310
$\{\sqrt{250} \leqslant m_i(t) < \sqrt{350}\} \cap T_i^\star > t$	Median	15.613	16.476	10.559	7.473	6.251	5.328	15.612	16.428	10.487	7.416	6.210	5.308
$\{\sqrt{250} \leqslant m_i(t) < \sqrt{350}\} \cap T_i^\star > t$	Bias	0.003	-0.030	-0.011	0.011	0.013	0.018	0.002	-0.078	-0.083	-0.046	-0.029	-0.002
$\{\sqrt{250} \leqslant m_i(t) < \sqrt{350}\} \cap T_i^\star > t$	Coverage	94.000	92.400	94.200	94.200	93.600	93.000	93.800	91.800	94.200	94.000	94.000	92.800
$\{\sqrt{350} \leqslant m_i(t) < \sqrt{500}\} \cap T_i^\star > t$	True	9.229	23.965	19.630	15.200	13.219	11.687	9.229	23.965	19.630	15.200	13.219	11.687
$\{\sqrt{350} \leqslant m_i(t) < \sqrt{500}\} \cap T_i^\star > t$	Median	9.251	23.936	19.562	15.134	13.160	11.634	9.246	23.928	19.503	15.079	13.122	11.621
$\{\sqrt{350} \leqslant m_i(t) < \sqrt{500}\} \cap T_i^\star > t$	Bias	0.022	-0.029	-0.068	-0.066	-0.059	-0.053	0.017	-0.038	-0.127	-0.121	-0.097	-0.066
$\{\sqrt{350} \leqslant m_i(t) < \sqrt{500}\} \cap T_i^\star > t$	Coverage	93.800	92.200	92.600	92.000	90.800	92.200	93.600	91.800	91.600	92.000	91.400	91.400
$\{\sqrt{500} \leqslant m_i(t)\} \cap T_i^\star > t$	True	2.880	24.354	29.626	30.016	27.051	24.708	2.880	24.354	29.626	30.016	27.051	24.708
$\{\sqrt{500} \leqslant m_i(t)\} \cap T_i^\star > t$	Median	2.909	24.405	29.696	30.082	27.091	24.719	2.904	24.433	29.706	30.085	27.100	24.732
$\{\sqrt{500} \leqslant m_i(t)\} \cap T_i^\star > t$	Bias	0.029	0.051	0.071	0.066	0.040	0.011	0.024	0.079	0.081	0.069	0.050	0.024
$\{\sqrt{500} \leqslant m_i(t)\} \cap T_i^\star > t$	Coverage	92.800	94.400	96.200	95.400	95.200	96.200	92.600	94.600	95.000	95.000	95.000	95.200

† Results from 500 replications with each dataset including 2000 individuals. The “true” marker evolution was based on linear splines with knots at 1 and 5 years since baseline and it was correctly specified in the fitted SPM-1 and SPM-2 models. “True” denotes the true parameter values; “Median” the mean of posterior medians over the 500 replications; “Bias” the mean bias for posterior median estimates; and “Coverage” the empirical coverage probability (%) of posterior credible intervals.

Table S3. Simulation study results for latent marker state probabilities (%) for group 1 ($w = 1$) when the data are simulated by SPM-2 and there is misclassification†.

State	Est	$t=0$	$t=2$	$t=4$	$t=6$	$t=8$	$t=10$	$t=0$	$t=2$	$t=4$	$t=6$	$t=8$	$t=10$
		Results from SPM-1						Results from SPM-2					
$\{m_i(t) < \sqrt{50}\} \cap T_i^\star > t$	True	12.431	0.198	0.048	0.031	0.014	0.005	12.431	0.198	0.048	0.031	0.014	0.005
$\{m_i(t) < \sqrt{50}\} \cap T_i^\star > t$	Median	12.476	0.203	0.050	0.033	0.014	0.006	12.469	0.202	0.051	0.034	0.016	0.007
$\{m_i(t) < \sqrt{50}\} \cap T_i^\star > t$	Bias	0.045	0.005	0.002	0.002	0.001	0.001	0.038	0.003	0.003	0.003	0.002	0.002
$\{m_i(t) < \sqrt{50}\} \cap T_i^\star > t$	Coverage	94.800	94.000	95.400	94.800	93.400	92.200	95.400	94.200	95.600	95.800	93.800	93.400
$\{\sqrt{50} \leqslant m_i(t) < \sqrt{100}\} \cap T_i^\star > t$	True	16.038	1.015	0.333	0.223	0.145	0.092	16.038	1.015	0.333	0.223	0.145	0.092
$\{\sqrt{50} \leqslant m_i(t) < \sqrt{100}\} \cap T_i^\star > t$	Median	16.037	1.041	0.348	0.236	0.152	0.098	16.038	1.025	0.347	0.236	0.154	0.101
$\{\sqrt{50} \leqslant m_i(t) < \sqrt{100}\} \cap T_i^\star > t$	Bias	-0.001	0.026	0.015	0.014	0.007	0.005	0.000	0.010	0.014	0.014	0.009	0.008
$\{\sqrt{50} \leqslant m_i(t) < \sqrt{100}\} \cap T_i^\star > t$	Coverage	95.000	93.600	94.000	93.400	92.800	93.400	95.600	94.800	94.800	94.000	94.000	93.200
$\{\sqrt{100} \leqslant m_i(t) < \sqrt{200}\} \cap T_i^\star > t$	True	31.710	6.990	3.111	2.110	1.643	1.305	31.710	6.990	3.111	2.110	1.643	1.305
$\{\sqrt{100} \leqslant m_i(t) < \sqrt{200}\} \cap T_i^\star > t$	Median	31.681	7.081	3.202	2.194	1.708	1.361	31.690	7.021	3.172	2.172	1.690	1.349
$\{\sqrt{100} \leqslant m_i(t) < \sqrt{200}\} \cap T_i^\star > t$	Bias	-0.029	0.091	0.091	0.085	0.065	0.057	-0.019	0.031	0.061	0.063	0.048	0.044
$\{\sqrt{100} \leqslant m_i(t) < \sqrt{200}\} \cap T_i^\star > t$	Coverage	94.800	93.400	93.400	91.200	91.400	91.200	94.600	94.400	94.400	94.000	93.800	92.800
$\{\sqrt{200} \leqslant m_i(t) < \sqrt{250}\} \cap T_i^\star > t$	True	12.103	6.336	3.415	2.364	1.953	1.650	12.103	6.336	3.415	2.364	1.953	1.650
$\{\sqrt{200} \leqslant m_i(t) < \sqrt{250}\} \cap T_i^\star > t$	Median	12.084	6.366	3.468	2.418	2.002	1.697	12.087	6.343	3.444	2.397	1.980	1.677
$\{\sqrt{200} \leqslant m_i(t) < \sqrt{250}\} \cap T_i^\star > t$	Bias	-0.019	0.031	0.053	0.054	0.049	0.047	-0.016	0.008	0.029	0.033	0.027	0.026
$\{\sqrt{200} \leqslant m_i(t) < \sqrt{250}\} \cap T_i^\star > t$	Coverage	93.600	92.400	91.600	91.000	92.200	90.600	94.200	93.000	93.000	92.600	93.400	93.200
$\{\sqrt{250} \leqslant m_i(t) < \sqrt{350}\} \cap T_i^\star > t$	True	15.610	16.278	10.503	7.565	6.483	5.674	15.610	16.278	10.503	7.565	6.483	5.674
$\{\sqrt{250} \leqslant m_i(t) < \sqrt{350}\} \cap T_i^\star > t$	Median	15.582	16.269	10.553	7.633	6.554	5.749	15.583	16.262	10.515	7.590	6.503	5.695
$\{\sqrt{250} \leqslant m_i(t) < \sqrt{350}\} \cap T_i^\star > t$	Bias	-0.027	-0.009	0.050	0.068	0.071	0.076	-0.027	-0.016	0.012	0.025	0.020	0.022
$\{\sqrt{250} \leqslant m_i(t) < \sqrt{350}\} \cap T_i^\star > t$	Coverage	94.800	93.400	94.000	92.800	91.000	93.200	93.600	93.000	94.800	93.200	93.400	93.800
$\{\sqrt{350} \leqslant m_i(t) < \sqrt{500}\} \cap T_i^\star > t$	True	9.229	23.750	19.550	15.361	13.602	12.257	9.229	23.750	19.550	15.361	13.602	12.257
$\{\sqrt{350} \leqslant m_i(t) < \sqrt{500}\} \cap T_i^\star > t$	Median	9.218	23.630	19.457	15.306	13.563	12.238	9.215	23.681	19.459	15.281	13.521	12.178
$\{\sqrt{350} \leqslant m_i(t) < \sqrt{500}\} \cap T_i^\star > t$	Bias	-0.011	-0.120	-0.093	-0.055	-0.039	-0.020	-0.014	-0.069	-0.091	-0.080	-0.081	-0.079
$\{\sqrt{350} \leqslant m_i(t) < \sqrt{500}\} \cap T_i^\star > t$	Coverage	95.200	94.000	94.200	94.000	93.400	93.600	95.400	94.000	94.200	93.200	93.000	93.400
$\{\sqrt{500} \leqslant m_i(t)\} \cap T_i^\star > t$	True	2.880	24.253	29.643	30.360	27.724	25.659	2.880	24.253	29.643	30.360	27.724	25.659
$\{\sqrt{500} \leqslant m_i(t)\} \cap T_i^\star > t$	Median	2.891	24.147	29.517	30.254	27.634	25.584	2.888	24.245	29.622	30.330	27.666	25.573
$\{\sqrt{500} \leqslant m_i(t)\} \cap T_i^\star > t$	Bias	0.011	-0.107	-0.126	-0.106	-0.090	-0.075	0.008	-0.008	-0.020	-0.030	-0.058	-0.087
$\{\sqrt{500} \leqslant m_i(t)\} \cap T_i^\star > t$	Coverage	94.600	94.200	95.600	95.200	94.200	95.600	95.000	95.200	95.800	94.400	94.200	94.400

† Results from 500 replications with each dataset including 2000 individuals. The “true” marker evolution was based on linear splines with knots at 1 and 5 years since baseline and it was correctly specified in the fitted SPM-1 and SPM-2 models. “True” denotes the true parameter values; “Median” the mean of posterior medians over the 500 replications; “Bias” the mean bias for posterior median estimates; and “Coverage” the empirical coverage probability (%) of posterior credible intervals.

Table S4. Simulation study results for latent marker state probabilities (%) for group 0 ($w = 0$) when the data are simulated by SPM-2 and there is misclassification†.

State	Est	$t=0$	$t=2$	$t=4$	$t=6$	$t=8$	$t=10$	$t=0$	$t=2$	$t=4$	$t=6$	$t=8$	$t=10$
		Results from SPM-1						Results from SPM-2					
$\{m_i(t) < \sqrt{50}\} \cap T_i^\star > t$	True	12.431	0.203	0.049	0.031	0.014	0.005	12.431	0.203	0.049	0.031	0.014	0.005
$\{m_i(t) < \sqrt{50}\} \cap T_i^\star > t$	Median	12.476	0.211	0.052	0.034	0.015	0.006	12.469	0.208	0.053	0.035	0.016	0.007
$\{m_i(t) < \sqrt{50}\} \cap T_i^\star > t$	Bias	0.045	0.008	0.003	0.003	0.002	0.002	0.038	0.004	0.004	0.004	0.002	0.002
$\{m_i(t) < \sqrt{50}\} \cap T_i^\star > t$	Coverage	94.800	94.000	95.200	95.000	93.600	92.400	95.400	94.400	95.800	95.200	93.800	94.400
$\{\sqrt{50} \leqslant m_i(t) < \sqrt{100}\} \cap T_i^\star > t$	True	16.038	1.028	0.335	0.223	0.145	0.092	16.038	1.028	0.335	0.223	0.145	0.092
$\{\sqrt{50} \leqslant m_i(t) < \sqrt{100}\} \cap T_i^\star > t$	Median	16.037	1.061	0.356	0.241	0.155	0.100	16.038	1.041	0.350	0.237	0.155	0.101
$\{\sqrt{50} \leqslant m_i(t) < \sqrt{100}\} \cap T_i^\star > t$	Bias	-0.001	0.033	0.020	0.018	0.011	0.008	0.000	0.013	0.015	0.014	0.010	0.009
$\{\sqrt{50} \leqslant m_i(t) < \sqrt{100}\} \cap T_i^\star > t$	Coverage	95.000	94.400	95.000	93.400	93.600	93.600	95.600	95.600	95.400	94.600	93.400	93.600
$\{\sqrt{100} \leqslant m_i(t) < \sqrt{200}\} \cap T_i^\star > t$	True	31.710	7.000	3.099	2.087	1.617	1.279	31.710	7.000	3.099	2.087	1.617	1.279
$\{\sqrt{100} \leqslant m_i(t) < \sqrt{200}\} \cap T_i^\star > t$	Median	31.681	7.116	3.207	2.184	1.690	1.339	31.690	7.041	3.164	2.153	1.670	1.329
$\{\sqrt{100} \leqslant m_i(t) < \sqrt{200}\} \cap T_i^\star > t$	Bias	-0.029	0.116	0.109	0.097	0.073	0.060	-0.019	0.042	0.065	0.066	0.053	0.050
$\{\sqrt{100} \leqslant m_i(t) < \sqrt{200}\} \cap T_i^\star > t$	Coverage	94.800	94.600	93.600	92.600	92.200	92.800	94.600	95.800	95.200	93.800	93.400	93.000
$\{\sqrt{200} \leqslant m_i(t) < \sqrt{250}\} \cap T_i^\star > t$	True	12.103	6.308	3.380	2.324	1.910	1.607	12.103	6.308	3.380	2.324	1.910	1.607
$\{\sqrt{200} \leqslant m_i(t) < \sqrt{250}\} \cap T_i^\star > t$	Median	12.084	6.357	3.445	2.384	1.961	1.650	12.087	6.322	3.412	2.359	1.941	1.637
$\{\sqrt{200} \leqslant m_i(t) < \sqrt{250}\} \cap T_i^\star > t$	Bias	-0.019	0.049	0.065	0.061	0.050	0.043	-0.016	0.014	0.032	0.035	0.030	0.030
$\{\sqrt{200} \leqslant m_i(t) < \sqrt{250}\} \cap T_i^\star > t$	Coverage	93.600	95.000	93.400	92.400	91.000	92.400	94.200	94.800	95.200	93.800	93.200	93.600
$\{\sqrt{250} \leqslant m_i(t) < \sqrt{350}\} \cap T_i^\star > t$	True	15.610	16.146	10.346	7.401	6.306	5.492	15.610	16.146	10.346	7.401	6.306	5.492
$\{\sqrt{250} \leqslant m_i(t) < \sqrt{350}\} \cap T_i^\star > t$	Median	15.582	16.182	10.427	7.484	6.376	5.549	15.583	16.140	10.365	7.434	6.334	5.520
$\{\sqrt{250} \leqslant m_i(t) < \sqrt{350}\} \cap T_i^\star > t$	Bias	-0.027	0.035	0.081	0.083	0.070	0.057	-0.027	-0.006	0.018	0.033	0.027	0.028
$\{\sqrt{250} \leqslant m_i(t) < \sqrt{350}\} \cap T_i^\star > t$	Coverage	94.800	94.400	93.800	93.400	93.800	92.800	93.600	93.400	95.000	93.600	93.600	93.600
$\{\sqrt{350} \leqslant m_i(t) < \sqrt{500}\} \cap T_i^\star > t$	True	9.229	23.477	19.167	14.953	13.157	11.794	9.229	23.477	19.167	14.953	13.157	11.794
$\{\sqrt{350} \leqslant m_i(t) < \sqrt{500}\} \cap T_i^\star > t$	Median	9.218	23.426	19.135	14.931	13.124	11.747	9.215	23.417	19.089	14.886	13.088	11.726
$\{\sqrt{350} \leqslant m_i(t) < \sqrt{500}\} \cap T_i^\star > t$	Bias	-0.011	-0.051	-0.031	-0.023	-0.033	-0.047	-0.014	-0.059	-0.078	-0.068	-0.069	-0.069
$\{\sqrt{350} \leqslant m_i(t) < \sqrt{500}\} \cap T_i^\star > t$	Coverage	95.200	94.400	95.000	93.400	94.400	93.600	95.400	94.000	94.400	94.200	94.200	94.600
$\{\sqrt{500} \leqslant m_i(t)\} \cap T_i^\star > t$	True	2.880	23.914	28.940	29.411	26.674	24.551	2.880	23.914	28.940	29.411	26.674	24.551
$\{\sqrt{500} \leqslant m_i(t)\} \cap T_i^\star > t$	Median	2.891	23.876	28.907	29.379	26.612	24.454	2.888	23.909	28.923	29.391	26.627	24.472
$\{\sqrt{500} \leqslant m_i(t)\} \cap T_i^\star > t$	Bias	0.011	-0.037	-0.033	-0.032	-0.062	-0.097	0.008	-0.005	-0.017	-0.020	-0.047	-0.078
$\{\sqrt{500} \leqslant m_i(t)\} \cap T_i^\star > t$	Coverage	94.600	93.400	93.600	94.000	93.600	93.200	95.000	94.000	94.800	93.800	93.200	93.600

† Results from 500 replications with each dataset including 2000 individuals. The "true" marker evolution was based on linear splines with knots at 1 and 5 years since baseline and it was correctly specified in the fitted SPM-1 and SPM-2 models. "True" denotes the true parameter values; "Median" the mean of posterior medians over the 500 replications; "Bias" the mean bias for posterior median estimates; and "Coverage" the empirical coverage probability (%) of posterior credible intervals.

7.2 *Simulation study results for transition marker state probabilities by baseline marker state*

In this subsection, we provide results for transition marker probabilities by baseline marker state in Tables S5-S8. Both models (SPM-1 and SPM-2) had great performance leading to estimates with small biases and acceptable coverage probabilities in almost all cases. However, when the data were generated from the SPM-1 model and the SPM-2 model was fitted, the coverage probability for the "$m_i(0) < \sqrt{50} \rightarrow \{m_i(6) < \sqrt{50}\} \cap (T_i^\star > t)$" transition was equal to 83.8% (Table S5).

Table S5. Simulation study results for transition marker state probabilities (%) by baseline marker state for group 1 ($w = 1$) when the data are simulated by SPM-1 and there is misclassification†.

transition	$t=2$	$t=4$	$t=6$	$t=8$	$t=10$	$t=2$	$t=4$	$t=6$	$t=8$	$t=10$
	Results from SPM-1					Results from SPM-2				
$m_i(0) < \sqrt{50} \rightarrow \{m_i(t) < \sqrt{50}\} \cap T_i^\star > t$	1.31/1.3/94.8	0.15/0.16/93.6	0.03/0.04/93.8	<0.01/0.01/93.4	<0.01/<0.01/94.2	1.31/1.32/94.8	0.15/0.19/90.8	0.03/0.05/83.8	<0.01/0.01/85.8	<0.01/<0.01/90.2
$m_i(0) < \sqrt{50} \rightarrow \{\sqrt{50} \leqslant m_i(t) < \sqrt{100}\} \cap T_i^\star > t$	5.86/5.84/94.2	1.09/1.12/93	0.36/0.39/92.6	0.13/0.16/93.2	0.03/0.05/93.2	5.86/5.77/94.8	1.09/1.17/91.4	0.36/0.44/87.6	0.13/0.2/85.2	0.03/0.08/85.4
$m_i(0) < \sqrt{50} \rightarrow \{\sqrt{100} \leqslant m_i(t) < \sqrt{200}\} \cap T_i^\star > t$	24.64/24.55/95	7.77/7.86/94.2	3.44/3.56/92.4	2.25/2.36/92.8	1.42/1.53/94	24.64/24.24/94.2	7.77/7.87/94.2	3.44/3.67/91.2	2.25/2.52/91.4	1.42/1.7/89.6
$m_i(0) < \sqrt{50} \rightarrow \{\sqrt{200} \leqslant m_i(t) < \sqrt{250}\} \cap T_i^\star > t$	12.55/12.49/94.8	6.45/6.46/95	3.4/3.44/93.4	2.57/2.61/94	1.95/2/94.2	12.55/12.36/92	6.45/6.42/94.8	3.4/3.47/93.6	2.57/2.67/92.8	1.95/2.08/92
$m_i(0) < \sqrt{50} \rightarrow \{\sqrt{250} \leqslant m_i(t) < \sqrt{350}\} \cap T_i^\star > t$	15.7/15.63/93.4	14.04/13.99/95.2	8.96/8.97/94.4	7.26/7.28/94.8	5.96/6/95	15.7/15.48/91.4	14.04/13.87/95.2	8.96/8.97/94.8	7.26/7.33/95.2	5.96/6.08/94.6
$m_i(0) < \sqrt{50} \rightarrow \{\sqrt{350} \leqslant m_i(t) < \sqrt{500}\} \cap T_i^\star > t$	6.3/6.29/93.8	14.75/14.63/93.2	13.06/12.96/94.6	11.18/11.1/93.8	9.71/9.64/95	6.3/6.23/93.4	14.75/14.51/92	13.06/12.9/93.6	11.18/11.08/93.8	9.71/9.65/95
$m_i(0) < \sqrt{50} \rightarrow \{\sqrt{500} \leqslant m_i(t)\} \cap T_i^\star > t$	0.66/0.67/93.8	7.56/7.52/93.4	12.77/12.7/94	11.4/11.32/93.4	10.31/10.23/93.8	0.66/0.66/93.8	7.56/7.46/92.8	12.77/12.63/93.6	11.4/11.27/93.2	10.31/10.17/92.8
$\sqrt{50} \leqslant m_i(0) < \sqrt{100} \rightarrow \{m_i(t) < \sqrt{50}\} \cap T_i^\star > t$	0.11/0.11/94.2	0.06/0.07/92.4	0.03/0.04/94.4	0.01/0.01/93.4	<0.01/<0.01/92.6	0.11/0.11/93.8	0.06/0.07/92.6	0.03/0.04/92.6	0.01/0.01/88.4	<0.01/<0.01/90.8
$\sqrt{50} \leqslant m_i(0) < \sqrt{100} \rightarrow \{\sqrt{50} \leqslant m_i(t) < \sqrt{100}\} \cap T_i^\star > t$	1.27/1.28/94.2	0.52/0.54/93.2	0.31/0.33/93.2	0.17/0.18/92.4	0.07/0.09/92.6	1.27/1.26/93.8	0.52/0.53/92.6	0.31/0.33/93.4	0.17/0.19/91.4	0.07/0.1/91.2
$\sqrt{50} \leqslant m_i(0) < \sqrt{100} \rightarrow \{\sqrt{100} \leqslant m_i(t) < \sqrt{200}\} \cap T_i^\star > t$	13.93/13.95/94.2	5.2/5.29/93.4	3.03/3.12/92.8	2.23/2.31/92.4	1.64/1.72/93.2	13.93/13.85/94.6	5.2/5.22/93.2	3.03/3.08/93.6	2.23/2.29/93	1.64/1.72/93.2
$\sqrt{50} \leqslant m_i(0) < \sqrt{100} \rightarrow \{\sqrt{200} \leqslant m_i(t) < \sqrt{250}\} \cap T_i^\star > t$	12.83/12.83/95.2	5.45/5.5/94.4	3.24/3.29/93.4	2.6/2.65/92	2.11/2.17/93.6	12.83/12.78/94.6	5.45/5.45/93.6	3.24/3.26/94	2.6/2.62/93.8	2.11/2.15/94.2
$\sqrt{50} \leqslant m_i(0) < \sqrt{100} \rightarrow \{\sqrt{250} \leqslant m_i(t) < \sqrt{350}\} \cap T_i^\star > t$	25.61/25.58/95	14.79/14.84/96	9.5/9.57/94	7.98/8.05/94.4	6.82/6.89/95	25.61/25.51/94.6	14.79/14.75/94.6	9.5/9.49/94.2	7.98/7.98/94.8	6.82/6.84/94.6
$\sqrt{50} \leqslant m_i(0) < \sqrt{100} \rightarrow \{\sqrt{350} \leqslant m_i(t) < \sqrt{500}\} \cap T_i^\star > t$	18.56/18.52/93.2	20.82/20.78/95.2	16.27/16.25/95.2	14.21/14.2/94	12.61/12.61/94.6	18.56/18.47/93.2	20.82/20.71/94.6	16.27/16.18/94.8	14.21/14.13/94.2	12.61/12.54/94.6
$\sqrt{50} \leqslant m_i(0) < \sqrt{100} \rightarrow \{\sqrt{500} \leqslant m_i(t)\} \cap T_i^\star > t$	3.83/3.84/94	15.7/15.67/94.2	20.71/20.68/93.6	18.71/18.68/93.2	17.11/17.08/91.6	3.83/3.82/93.4	15.7/15.65/93.4	20.71/20.66/93.2	18.71/18.65/93.4	17.11/17.02/92.2
$\sqrt{100} \leqslant m_i(0) < \sqrt{200} \rightarrow \{m_i(t) < \sqrt{50}\} \cap T_i^\star > t$	0.01/0.01/95.4	0.02/0.02/93	0.02/0.02/93.2	0.01/0.01/93.8	<0.01/<0.01/92.4	0.01/0.01/95.4	0.02/0.02/93.8	0.02/0.02/94.6	0.01/0.01/93.8	<0.01/<0.01/92.6
$\sqrt{100} \leqslant m_i(0) < \sqrt{200} \rightarrow \{\sqrt{50} \leqslant m_i(t) < \sqrt{100}\} \cap T_i^\star > t$	0.22/0.23/94.4	0.22/0.23/93.4	0.2/0.21/93.2	0.12/0.13/92.6	0.07/0.08/91.8	0.22/0.22/94.6	0.22/0.22/94.2	0.2/0.21/94.2	0.12/0.13/93	0.07/0.08/93.4
$\sqrt{100} \leqslant m_i(0) < \sqrt{200} \rightarrow \{\sqrt{100} \leqslant m_i(t) < \sqrt{200}\} \cap T_i^\star > t$	4.96/4.97/95.4	2.93/2.97/95	2.19/2.26/94.2	1.68/1.73/93.2	1.3/1.36/93.6	4.96/4.95/95.4	2.93/2.94/95	2.19/2.21/94.6	1.68/1.69/94.2	1.3/1.32/94.4
$\sqrt{100} \leqslant m_i(0) < \sqrt{200} \rightarrow \{\sqrt{200} \leqslant m_i(t) < \sqrt{250}\} \cap T_i^\star > t$	7.19/7.19/95.8	3.73/3.75/94.6	2.6/2.64/93.8	2.13/2.17/93.8	1.78/1.82/94	7.19/7.19/95.6	3.73/3.73/96	2.6/2.6/94.8	2.13/2.13/94.4	1.78/1.78/94.6
$\sqrt{100} \leqslant m_i(0) < \sqrt{200} \rightarrow \{\sqrt{250} \leqslant m_i(t) < \sqrt{350}\} \cap T_i^\star > t$	22.83/22.84/94.8	12.37/12.41/95.6	8.53/8.58/94.4	7.27/7.33/94.8	6.31/6.38/94.8	22.83/22.88/95.2	12.37/12.38/96.4	8.53/8.52/95	7.27/7.25/95.6	6.31/6.29/94.8
$\sqrt{100} \leqslant m_i(0) < \sqrt{200} \rightarrow \{\sqrt{350} \leqslant m_i(t) < \sqrt{500}\} \cap T_i^\star > t$	31.24/31.23/94.8	23.02/23.02/94.2	17.15/17.15/94.2	15.12/15.13/95	13.55/13.57/95.4	31.24/31.29/95	23.02/23.03/95	17.15/17.11/93.2	15.12/15.07/94	13.55/13.48/94.8
$\sqrt{100} \leqslant m_i(0) < \sqrt{200} \rightarrow \{\sqrt{500} \leqslant m_i(t)\} \cap T_i^\star > t$	14.48/14.49/94.2	26.38/26.42/94	28.92/28.98/93.4	26.31/26.35/93.2	24.23/24.25/93.6	14.48/14.51/94	26.38/26.48/92.8	28.92/29.04/92.6	26.31/26.36/92.2	24.23/24.21/93.6
$\sqrt{200} \leqslant m_i(0) < \sqrt{250} \rightarrow \{m_i(t) < \sqrt{50}\} \cap T_i^\star > t$	<0.01/<0.01/96.2	0.01/0.01/94.2	0.01/0.01/94.4	0.01/0.01/94.6	<0.01/<0.01/93.4	<0.01/<0.01/95.4	0.01/0.01/93.4	0.01/0.01/94	0.01/0.01/94.8	<0.01/<0.01/94.2
$\sqrt{200} \leqslant m_i(0) < \sqrt{250} \rightarrow \{\sqrt{50} \leqslant m_i(t) < \sqrt{100}\} \cap T_i^\star > t$	0.03/0.03/96.4	0.09/0.09/94.2	0.12/0.13/93.8	0.08/0.08/94	0.05/0.05/94	0.03/0.03/96	0.09/0.09/94	0.12/0.12/94	0.08/0.08/94.2	0.05/0.05/95.2
$\sqrt{200} \leqslant m_i(0) < \sqrt{250} \rightarrow \{\sqrt{100} \leqslant m_i(t) < \sqrt{200}\} \cap T_i^\star > t$	1.32/1.33/96.8	1.56/1.58/95.2	1.53/1.57/94	1.2/1.23/94.2	0.96/0.99/93.6	1.32/1.33/96.2	1.56/1.56/95.2	1.53/1.53/94.2	1.2/1.19/93.8	0.96/0.95/94.8
$\sqrt{200} \leqslant m_i(0) < \sqrt{250} \rightarrow \{\sqrt{200} \leqslant m_i(t) < \sqrt{250}\} \cap T_i^\star > t$	2.94/2.95/95.2	2.37/2.38/95.4	2/2.02/94.8	1.66/1.68/93.6	1.4/1.43/93.4	2.94/2.95/94.8	2.37/2.37/96	2/2/95	1.66/1.64/94	1.4/1.39/93.8
$\sqrt{200} \leqslant m_i(0) < \sqrt{250} \rightarrow \{\sqrt{250} \leqslant m_i(t) < \sqrt{350}\} \cap T_i^\star > t$	14.64/14.64/97.2	9.45/9.46/96.4	7.26/7.29/95.4	6.24/6.27/94.2	5.46/5.51/94.6	14.64/14.69/96	9.45/9.46/96	7.26/7.24/95.8	6.24/6.19/94.4	5.46/5.41/94.4
$\sqrt{200} \leqslant m_i(0) < \sqrt{250} \rightarrow \{\sqrt{350} \leqslant m_i(t) < \sqrt{500}\} \cap T_i^\star > t$	34.65/34.65/95.2	22.37/22.36/94.2	16.78/16.76/94.2	14.88/14.87/94.4	13.4/13.4/93.6	34.65/34.8/94.6	22.37/22.42/93.8	16.78/16.75/93.4	14.88/14.81/94.6	13.4/13.3/93
$\sqrt{200} \leqslant m_i(0) < \sqrt{250} \rightarrow \{\sqrt{500} \leqslant m_i(t)\} \cap T_i^\star > t$	30.23/30.25/96.6	36.64/36.75/95.2	36.1/36.23/93.8	32.96/33.08/93	30.46/30.56/93.8	30.23/30.36/96.2	36.64/36.91/93.6	36.1/36.35/92.6	32.96/33.12/92.6	30.46/30.52/93.4
$\sqrt{250} \leqslant m_i(0) < \sqrt{350} \rightarrow \{m_i(t) < \sqrt{50}\} \cap T_i^\star > t$	<0.01/<0.01/96	<0.01/<0.01/95.2	0.01/0.01/95.2	<0.01/<0.01/95.2	<0.01/<0.01/93.6	<0.01/<0.01/96.2	<0.01/<0.01/93.8	0.01/0.01/94.4	<0.01/<0.01/94.6	<0.01/<0.01/94.6
$\sqrt{250} \leqslant m_i(0) < \sqrt{350} \rightarrow \{\sqrt{50} \leqslant m_i(t) < \sqrt{100}\} \cap T_i^\star > t$	0.01/0.01/96.2	0.04/0.04/95.2	0.08/0.08/94	0.05/0.06/95.2	0.04/0.04/94.8	0.01/0.01/95.8	0.04/0.04/94.6	0.08/0.08/93.8	0.05/0.05/94.8	0.04/0.04/95.4
$\sqrt{250} \leqslant m_i(0) < \sqrt{350} \rightarrow \{\sqrt{100} \leqslant m_i(t) < \sqrt{200}\} \cap T_i^\star > t$	0.43/0.44/96.2	0.91/0.93/95.2	1.12/1.14/94	0.88/0.91/94.6	0.71/0.74/94	0.43/0.44/96.6	0.91/0.92/95.8	1.12/1.11/94.6	0.88/0.87/94.8	0.71/0.7/95.2
$\sqrt{250} \leqslant m_i(0) < \sqrt{350} \rightarrow \{\sqrt{200} \leqslant m_i(t) < \sqrt{250}\} \cap T_i^\star > t$	1.22/1.23/95.8	1.56/1.57/95.2	1.56/1.58/95.2	1.3/1.32/95.4	1.11/1.13/93.8	1.22/1.23/95.4	1.56/1.56/96	1.56/1.56/95.2	1.3/1.29/95.2	1.11/1.09/94.6
$\sqrt{250} \leqslant m_i(0) < \sqrt{350} \rightarrow \{\sqrt{250} \leqslant m_i(t) < \sqrt{350}\} \cap T_i^\star > t$	8.27/8.27/95.2	7.11/7.11/94.8	6.12/6.14/94.6	5.29/5.3/95.2	4.65/4.68/94.8	8.27/8.3/94.6	7.11/7.11/95	6.12/6.1/94.4	5.29/5.23/93.8	4.65/4.59/94.6
$\sqrt{250} \leqslant m_i(0) < \sqrt{350} \rightarrow \{\sqrt{350} \leqslant m_i(t) < \sqrt{500}\} \cap T_i^\star > t$	29.51/29.48/95.4	20.18/20.14/94.4	15.77/15.72/93	14.02/13.99/95.4	12.67/12.65/93.4	29.51/29.63/96.6	20.18/20.2/95	15.77/15.71/93	14.02/13.92/94.6	12.67/12.54/93
$\sqrt{250} \leqslant m_i(0) < \sqrt{350} \rightarrow \{\sqrt{500} \leqslant m_i(t)\} \cap T_i^\star > t$	46.01/46.05/95	44.93/45.09/94.8	41.65/41.84/94.4	38.13/38.3/93.8	35.34/35.47/94.2	46.01/46.28/95.2	44.93/45.33/94	41.65/42.01/94.2	38.13/38.35/93.6	35.34/35.42/95
$\sqrt{350} \leqslant m_i(0) < \sqrt{500} \rightarrow \{m_i(t) < \sqrt{50}\} \cap T_i^\star > t$	<0.01/<0.01/96.2	<0.01/<0.01/94.8	<0.01/<0.01/94.4	<0.01/<0.01/95.2	<0.01/<0.01/94.2	<0.01/<0.01/95.2	<0.01/<0.01/93.8	<0.01/<0.01/93.8	<0.01/<0.01/94.6	<0.01/<0.01/95.2
$\sqrt{350} \leqslant m_i(0) < \sqrt{500} \rightarrow \{\sqrt{50} \leqslant m_i(t) < \sqrt{100}\} \cap T_i^\star > t$	<0.01/<0.01/96	0.01/0.01/94.6	0.04/0.04/94.4	0.03/0.03/95	0.02/0.02/95.2	<0.01/<0.01/95.8	0.01/0.01/94.2	0.04/0.04/94	0.03/0.03/93.8	0.02/0.02/94
$\sqrt{350} \leqslant m_i(0) < \sqrt{500} \rightarrow \{\sqrt{100} \leqslant m_i(t) < \sqrt{200}\} \cap T_i^\star > t$	0.06/0.07/95.6	0.38/0.39/95	0.67/0.69/95.2	0.54/0.55/95.2	0.44/0.45/94.8	0.06/0.07/95.4	0.38/0.39/95.2	0.67/0.67/94.4	0.54/0.53/94	0.44/0.43/94.6
$\sqrt{350} \leqslant m_i(0) < \sqrt{500} \rightarrow \{\sqrt{200} \leqslant m_i(t) < \sqrt{250}\} \cap T_i^\star > t$	0.26/0.26/95.8	0.78/0.78/94.2	1.04/1.05/94.6	0.87/0.88/95.2	0.75/0.76/94.8	0.26/0.26/95.2	0.78/0.78/95	1.04/1.03/94.2	0.87/0.86/93.6	0.75/0.73/94.2
$\sqrt{350} \leqslant m_i(0) < \sqrt{500} \rightarrow \{\sqrt{250} \leqslant m_i(t) < \sqrt{350}\} \cap T_i^\star > t$	2.72/2.73/95.4	4.29/4.28/93.8	4.56/4.55/93.8	3.95/3.95/94	3.49/3.5/94.6	2.72/2.74/94.6	4.29/4.29/94.2	4.56/4.52/93.4	3.95/3.89/93.4	3.49/3.43/94.2
$\sqrt{350} \leqslant m_i(0) < \sqrt{500} \rightarrow \{\sqrt{350} \leqslant m_i(t) < \sqrt{500}\} \cap T_i^\star > t$	17.44/17.41/94.6	15.79/15.7/94.4	13.69/13.61/94	12.21/12.14/94.6	11.07/11.01/94.4	17.44/17.49/94.6	15.79/15.75/94.4	13.69/13.59/93.6	12.21/12.07/94.4	11.07/10.9/93.8
$\sqrt{350} \leqslant m_i(0) < \sqrt{500} \rightarrow \{\sqrt{500} \leqslant m_i(t)\} \cap T_i^\star > t$	66.74/66.77/93.8	56.02/56.21/95.4	49.19/49.42/95.4	45.17/45.37/95.6	41.98/42.13/95.2	66.74/67.16/93.4	56.02/56.55/94.6	49.19/49.63/95.8	45.17/45.43/94.8	41.98/42.05/95.4
$\sqrt{500} \leqslant m_i(0) \rightarrow \{m_i(t) < \sqrt{50}\} \cap T_i^\star > t$	<0.01/<0.01/95.8	<0.01/<0.01/94.6	<0.01/<0.01/94.6	<0.01/<0.01/94.6	<0.01/<0.01/95.4	<0.01/<0.01/95.4	<0.01/<0.01/94.6	<0.01/<0.01/94.2	<0.01/<0.01/94.4	<0.01/<0.01/95
$\sqrt{500} \leqslant m_i(0) \rightarrow \{\sqrt{50} \leqslant m_i(t) < \sqrt{100}\} \cap T_i^\star > t$	<0.01/<0.01/95.4	<0.01/<0.01/94.6	0.02/0.02/94.8	0.01/0.01/94.2	0.01/0.01/94.2	<0.01/<0.01/95	<0.01/<0.01/94.4	0.02/0.02/94.6	0.01/0.01/93.4	0.01/0.01/93.6
$\sqrt{500} \leqslant m_i(0) \rightarrow \{\sqrt{100} \leqslant m_i(t) < \sqrt{200}\} \cap T_i^\star > t$	<0.01/<0.01/95.2	0.11/0.11/95.2	0.31/0.32/94.2	0.25/0.26/93.4	0.21/0.22/94.8	<0.01/<0.01/94.6	0.11/0.11/95	0.31/0.31/94.6	0.25/0.25/93.8	0.21/0.21/93.4
$\sqrt{500} \leqslant m_i(0) \rightarrow \{\sqrt{200} \leqslant m_i(t) < \sqrt{250}\} \cap T_i^\star > t$	0.02/0.02/94.8	0.27/0.27/94.6	0.55/0.56/94.2	0.47/0.47/93.6	0.4/0.41/93.8	0.02/0.02/94.6	0.27/0.27/94.6	0.55/0.55/94.6	0.47/0.46/94	0.4/0.39/93.8
$\sqrt{500} \leqslant m_i(0) \rightarrow \{\sqrt{250} \leqslant m_i(t) < \sqrt{350}\} \cap T_i^\star > t$	0.4/0.41/94.6	1.88/1.87/94.2	2.81/2.8/93.6	2.45/2.44/93.8	2.17/2.17/94	0.4/0.41/94	1.88/1.87/94.2	2.81/2.78/94.2	2.45/2.4/93.4	2.17/2.12/94.4
$\sqrt{500} \leqslant m_i(0) \rightarrow \{\sqrt{350} \leqslant m_i(t) < \sqrt{500}\} \cap T_i^\star > t$	5.36/5.38/94.2	9.59/9.49/94	10.33/10.21/94.4	9.24/9.14/94.6	8.4/8.32/94.8	5.36/5.4/94.2	9.59/9.52/94.2	10.33/10.2/93.4	9.24/9.07/93.2	8.4/8.21/93.2
$\sqrt{500} \leqslant m_i(0) \rightarrow \{\sqrt{500} \leqslant m_i(t)\} \cap T_i^\star > t$	83.1/83.07/93.4	67.94/68.08/94.6	58.09/58.3/94.6	53.53/53.72/94.8	49.9/50.05/94.8	83.1/83.56/91.4	67.94/68.53/93.2	58.09/58.55/95	53.53/53.76/94.8	49.9/49.91/94.4

† Results from 500 replications with each dataset including 2000 individuals. The "true" marker evolution was based on linear splines with knots at 1 and 5 years since baseline and it was correctly specified in the fitted SPM-1 and SPM-2 models. The true parameter values, the mean of posterior medians over the 500 replications and the empirical coverage probability (%) of posterior credible intervals are presented, respectively.

Table S6. Simulation study results for transition marker state probabilities (%) by baseline marker state for group 0 ($w = 0$) when the data are simulated by SPM-1 and there is misclassification†.

transition	$t = 2$	$t = 4$	$t = 6$	$t = 8$	$t = 10$	$t = 2$	$t = 4$	$t = 6$	$t = 8$	$t = 10$
	Results from SPM-1					Results from SPM-2				
$m_i(0) < \sqrt{50} \rightarrow \{m_i(t) < \sqrt{50}\} \cap T_i^\star > t$	1.38/1.37/94.6	0.17/0.18/94.4	0.03/0.04/92	<0.01/0.01/92	<0.01/<0.01/93.8	1.38/1.36/95.2	0.17/0.19/93.2	0.03/0.05/89.2	<0.01/0.01/90	<0.01/<0.01/93.4
$m_i(0) < \sqrt{50} \rightarrow \{\sqrt{50} \leqslant m_i(t) < \sqrt{100}\} \cap T_i^\star > t$	6.02/5.99/95.4	1.14/1.16/94.2	0.38/0.41/93.4	0.14/0.16/91.2	0.03/0.05/92.6	6.02/5.86/94	1.14/1.17/94	0.38/0.44/91.4	0.14/0.19/90	0.03/0.07/89.6
$m_i(0) < \sqrt{50} \rightarrow \{\sqrt{100} \leqslant m_i(t) < \sqrt{200}\} \cap T_i^\star > t$	24.91/24.78/94.6	7.9/7.95/95.4	3.48/3.57/93.8	2.25/2.32/93.6	1.39/1.47/93	24.91/24.37/92.8	7.9/7.86/94.4	3.48/3.61/94.8	2.25/2.43/92.6	1.39/1.61/89.4
$m_i(0) < \sqrt{50} \rightarrow \{\sqrt{200} \leqslant m_i(t) < \sqrt{250}\} \cap T_i^\star > t$	12.6/12.52/92.8	6.47/6.46/94.8	3.39/3.41/93.8	2.53/2.55/94.2	1.88/1.91/93.2	12.6/12.35/90.2	6.47/6.37/93.6	3.39/3.4/93.8	2.53/2.59/93.4	1.88/1.99/90.8
$m_i(0) < \sqrt{50} \rightarrow \{\sqrt{250} \leqslant m_i(t) < \sqrt{350}\} \cap T_i^\star > t$	15.69/15.61/94.4	13.99/13.9/94.8	8.85/8.82/94.6	7.07/7.05/94.6	5.72/5.71/95.2	15.69/15.42/92	13.99/13.71/91.8	8.85/8.77/93.6	7.07/7.09/94.8	5.72/5.82/93
$m_i(0) < \sqrt{50} \rightarrow \{\sqrt{350} \leqslant m_i(t) < \sqrt{500}\} \cap T_i^\star > t$	6.27/6.26/93.6	14.6/14.45/93.2	12.8/12.66/91.8	10.81/10.69/92.4	9.27/9.15/93.4	6.27/6.19/93.8	14.6/14.28/91.4	12.8/12.57/91.2	10.81/10.67/91.8	9.27/9.21/94
$m_i(0) < \sqrt{50} \rightarrow \{\sqrt{500} \leqslant m_i(t)\} \cap T_i^\star > t$	0.66/0.67/93.6	7.44/7.39/94	12.44/12.34/93.2	10.96/10.86/93.8	9.8/9.69/93.6	0.66/0.66/93.6	7.44/7.31/93	12.44/12.24/93.2	10.96/10.82/93.6	9.8/9.68/93.4
$\sqrt{50} \leqslant m_i(0) < \sqrt{100} \rightarrow \{m_i(t) < \sqrt{50}\} \cap T_i^\star > t$	0.11/0.11/95	0.06/0.07/93.2	0.04/0.04/94	0.01/0.01/92.2	<0.01/<0.01/93.4	0.11/0.11/94	0.06/0.07/94.4	0.04/0.04/92.2	0.01/0.01/92	<0.01/<0.01/92.6
$\sqrt{50} \leqslant m_i(0) < \sqrt{100} \rightarrow \{\sqrt{50} \leqslant m_i(t) < \sqrt{100}\} \cap T_i^\star > t$	1.29/1.29/94.4	0.53/0.55/94	0.32/0.33/93.6	0.17/0.18/93.4	0.07/0.08/92.8	1.29/1.27/94.2	0.53/0.53/94.2	0.32/0.33/94.4	0.17/0.18/91.6	0.07/0.09/92.4
$\sqrt{50} \leqslant m_i(0) < \sqrt{100} \rightarrow \{\sqrt{100} \leqslant m_i(t) < \sqrt{200}\} \cap T_i^\star > t$	13.95/13.95/94.4	5.19/5.26/93.8	3/3.07/94.2	2.18/2.24/94	1.58/1.63/93.6	13.95/13.82/93.8	5.19/5.17/94.2	3/3.02/94.2	2.18/2.21/94.6	1.58/1.64/93
$\sqrt{50} \leqslant m_i(0) < \sqrt{100} \rightarrow \{\sqrt{200} \leqslant m_i(t) < \sqrt{250}\} \cap T_i^\star > t$	12.8/12.79/94	5.4/5.43/95	3.18/3.22/93.4	2.52/2.55/95	2.02/2.05/93.6	12.8/12.7/92.8	5.4/5.36/93.8	3.18/3.17/94	2.52/2.52/95.4	2.02/2.05/94.4
$\sqrt{50} \leqslant m_i(0) < \sqrt{100} \rightarrow \{\sqrt{250} \leqslant m_i(t) < \sqrt{350}\} \cap T_i^\star > t$	25.47/25.42/94.4	14.59/14.6/95	9.27/9.3/92.8	7.68/7.71/93.6	6.48/6.51/93.2	25.47/25.29/94	14.59/14.47/94	9.27/9.22/93.4	7.68/7.67/94	6.48/6.51/93
$\sqrt{50} \leqslant m_i(0) < \sqrt{100} \rightarrow \{\sqrt{350} \leqslant m_i(t) < \sqrt{500}\} \cap T_i^\star > t$	18.41/18.36/94.8	20.45/20.37/93.8	15.79/15.73/93	13.62/13.56/92.6	11.93/11.89/92.6	18.41/18.27/94.2	20.45/20.25/92.8	15.79/15.65/92.4	13.62/13.53/93.4	11.93/11.9/92.2
$\sqrt{50} \leqslant m_i(0) < \sqrt{100} \rightarrow \{\sqrt{500} \leqslant m_i(t)\} \cap T_i^\star > t$	3.79/3.8/94.6	15.36/15.32/95	20.02/19.97/95.4	17.87/17.8/95.6	16.17/16.09/95	3.79/3.77/94	15.36/15.25/94.6	20.02/19.92/95.2	17.87/17.8/95.2	16.17/16.11/95.6
$\sqrt{100} \leqslant m_i(0) < \sqrt{200} \rightarrow \{m_i(t) < \sqrt{50}\} \cap T_i^\star > t$	0.01/0.01/95.4	0.02/0.02/95.2	0.02/0.03/94	0.01/0.01/92.4	<0.01/<0.01/93.2	0.01/0.01/95.4	0.02/0.02/94.8	0.02/0.02/95.2	0.01/0.01/93.2	<0.01/<0.01/93.2
$\sqrt{100} \leqslant m_i(0) < \sqrt{200} \rightarrow \{\sqrt{50} \leqslant m_i(t) < \sqrt{100}\} \cap T_i^\star > t$	0.23/0.23/95.2	0.22/0.23/95.6	0.2/0.21/95	0.12/0.13/94	0.07/0.07/92.6	0.23/0.22/95.6	0.22/0.22/95.8	0.2/0.2/95	0.12/0.12/94.8	0.07/0.07/92.6
$\sqrt{100} \leqslant m_i(0) < \sqrt{200} \rightarrow \{\sqrt{100} \leqslant m_i(t) < \sqrt{200}\} \cap T_i^\star > t$	4.95/4.95/96.2	2.9/2.93/95.8	2.15/2.2/95.6	1.63/1.67/95	1.25/1.28/95	4.95/4.93/96	2.9/2.89/96	2.15/2.15/97.2	1.63/1.63/96.4	1.25/1.26/95.2
$\sqrt{100} \leqslant m_i(0) < \sqrt{200} \rightarrow \{\sqrt{200} \leqslant m_i(t) < \sqrt{250}\} \cap T_i^\star > t$	7.14/7.14/96.6	3.67/3.69/95.6	2.53/2.56/94.6	2.05/2.07/95.6	1.69/1.71/94	7.14/7.12/96.4	3.67/3.65/96.4	2.53/2.52/94.8	2.05/2.04/96.6	1.69/1.69/93.6
$\sqrt{100} \leqslant m_i(0) < \sqrt{200} \rightarrow \{\sqrt{250} \leqslant m_i(t) < \sqrt{350}\} \cap T_i^\star > t$	22.64/22.64/93.6	12.14/12.15/95	8.27/8.3/93.2	6.96/6.99/93.6	5.96/6/93.4	22.64/22.61/92.8	12.14/12.09/94	8.27/8.23/94.6	6.96/6.93/94	5.96/5.96/93
$\sqrt{100} \leqslant m_i(0) < \sqrt{200} \rightarrow \{\sqrt{350} \leqslant m_i(t) < \sqrt{500}\} \cap T_i^\star > t$	30.91/30.89/95.2	22.51/22.48/94.6	16.56/16.53/94	14.43/14.4/93	12.77/12.75/92.8	30.91/30.86/95.2	22.51/22.43/94	16.56/16.48/92.6	14.43/14.36/92.8	12.77/12.74/93
$\sqrt{100} \leqslant m_i(0) < \sqrt{200} \rightarrow \{\sqrt{500} \leqslant m_i(t)\} \cap T_i^\star > t$	14.31/14.31/95	25.73/25.75/95.4	27.87/27.89/94.6	25.05/25.05/95.2	22.83/22.8/96.8	14.31/14.29/94.6	25.73/25.73/94.2	27.87/27.89/94.8	25.05/25.07/95.2	22.83/22.84/96.2
$\sqrt{200} \leqslant m_i(0) < \sqrt{250} \rightarrow \{m_i(t) < \sqrt{50}\} \cap T_i^\star > t$	<0.01/<0.01/96.2	0.01/0.01/95.8	0.01/0.01/94.8	0.01/0.01/94	<0.01/<0.01/93.2	<0.01/<0.01/96.2	0.01/0.01/94.4	0.01/0.01/95	0.01/0.01/94.2	<0.01/<0.01/93.8
$\sqrt{200} \leqslant m_i(0) < \sqrt{250} \rightarrow \{\sqrt{50} \leqslant m_i(t) < \sqrt{100}\} \cap T_i^\star > t$	0.03/0.03/96	0.09/0.09/95.8	0.12/0.13/95.8	0.08/0.08/94.2	0.05/0.05/93.6	0.03/0.03/95.6	0.09/0.09/94.6	0.12/0.12/95.6	0.08/0.08/94.2	0.05/0.05/94.2
$\sqrt{200} \leqslant m_i(0) < \sqrt{250} \rightarrow \{\sqrt{100} \leqslant m_i(t) < \sqrt{200}\} \cap T_i^\star > t$	1.32/1.32/96.6	1.54/1.55/95.6	1.5/1.53/96.6	1.16/1.18/95.6	0.91/0.94/94.8	1.32/1.32/96.2	1.54/1.53/96.2	1.5/1.49/96.8	1.16/1.15/95.4	0.91/0.9/94.2
$\sqrt{200} \leqslant m_i(0) < \sqrt{250} \rightarrow \{\sqrt{200} \leqslant m_i(t) < \sqrt{250}\} \cap T_i^\star > t$	2.92/2.92/96.2	2.33/2.33/96.4	1.94/1.96/96.2	1.59/1.6/95	1.33/1.34/94.8	2.92/2.92/96.2	2.33/2.32/96.2	1.94/1.93/96.6	1.59/1.57/96.2	1.33/1.32/94.2
$\sqrt{200} \leqslant m_i(0) < \sqrt{250} \rightarrow \{\sqrt{250} \leqslant m_i(t) < \sqrt{350}\} \cap T_i^\star > t$	14.49/14.48/96.8	9.24/9.24/95.4	7.01/7.02/95.2	5.95/5.97/95	5.15/5.17/94.2	14.49/14.49/96.6	9.24/9.21/95.4	7.01/6.97/95.6	5.95/5.91/94.6	5.15/5.12/94.6
$\sqrt{200} \leqslant m_i(0) < \sqrt{250} \rightarrow \{\sqrt{350} \leqslant m_i(t) < \sqrt{500}\} \cap T_i^\star > t$	34.23/34.22/93.6	21.83/21.79/92.6	16.16/16.12/92.8	14.16/14.12/92.2	12.61/12.57/92.6	34.23/34.27/93.8	21.83/21.78/93.6	16.16/16.09/93	14.16/14.08/92.4	12.61/12.54/92.6
$\sqrt{200} \leqslant m_i(0) < \sqrt{250} \rightarrow \{\sqrt{500} \leqslant m_i(t)\} \cap T_i^\star > t$	29.84/29.85/96.8	35.69/35.77/96.4	34.72/34.82/96.4	31.35/31.41/96.2	28.67/28.71/95.4	29.84/29.88/95.6	35.69/35.82/96.4	34.72/34.86/96	31.35/31.45/95.4	28.67/28.74/95.8
$\sqrt{250} \leqslant m_i(0) < \sqrt{350} \rightarrow \{m_i(t) < \sqrt{50}\} \cap T_i^\star > t$	<0.01/<0.01/96.6	<0.01/<0.01/95.4	0.01/0.01/94.4	<0.01/<0.01/93.4	<0.01/<0.01/94	<0.01/<0.01/95.8	<0.01/<0.01/95	0.01/0.01/95.2	<0.01/<0.01/94.2	<0.01/<0.01/93.6
$\sqrt{250} \leqslant m_i(0) < \sqrt{350} \rightarrow \{\sqrt{50} \leqslant m_i(t) < \sqrt{100}\} \cap T_i^\star > t$	0.01/0.01/96.4	0.04/0.04/95.6	0.08/0.08/94.6	0.05/0.06/95.4	0.03/0.04/94.4	0.01/0.01/95.8	0.04/0.04/94.6	0.08/0.08/95	0.05/0.05/93.6	0.03/0.03/94.8
$\sqrt{250} \leqslant m_i(0) < \sqrt{350} \rightarrow \{\sqrt{100} \leqslant m_i(t) < \sqrt{200}\} \cap T_i^\star > t$	0.43/0.43/96	0.9/0.91/95.2	1.08/1.11/96.2	0.85/0.87/95.6	0.68/0.69/95.2	0.43/0.43/96.2	0.9/0.9/95.4	1.08/1.08/95.6	0.85/0.84/95	0.68/0.67/94.2
$\sqrt{250} \leqslant m_i(0) < \sqrt{350} \rightarrow \{\sqrt{200} \leqslant m_i(t) < \sqrt{250}\} \cap T_i^\star > t$	1.21/1.22/95.8	1.53/1.53/95.6	1.51/1.52/95.8	1.25/1.26/95.6	1.05/1.06/95.6	1.21/1.22/96	1.53/1.52/95.6	1.51/1.5/95.2	1.25/1.23/94.8	1.05/1.04/94.8
$\sqrt{250} \leqslant m_i(0) < \sqrt{350} \rightarrow \{\sqrt{250} \leqslant m_i(t) < \sqrt{350}\} \cap T_i^\star > t$	8.18/8.17/96	6.94/6.93/94.6	5.91/5.91/95.4	5.04/5.04/95.4	4.38/4.39/95	8.18/8.18/95.4	6.94/6.91/95.4	5.91/5.86/94.6	5.04/4.98/95.2	4.38/4.33/95.2
$\sqrt{250} \leqslant m_i(0) < \sqrt{350} \rightarrow \{\sqrt{350} \leqslant m_i(t) < \sqrt{500}\} \cap T_i^\star > t$	29.14/29.1/94.8	19.67/19.61/93.4	15.17/15.11/92.2	13.33/13.27/92	11.91/11.85/91.8	29.14/29.16/95	19.67/19.61/94.4	15.17/15.07/93.4	13.33/13.22/92.2	11.91/11.81/92.2
$\sqrt{250} \leqslant m_i(0) < \sqrt{350} \rightarrow \{\sqrt{500} \leqslant m_i(t)\} \cap T_i^\star > t$	45.38/45.41/95.6	43.73/43.87/96.6	40.04/40.19/95.6	36.24/36.36/95.6	33.25/33.31/96	45.38/45.52/95.8	43.73/43.96/96.6	40.04/40.26/95.4	36.24/36.39/95.6	33.25/33.33/95.4
$\sqrt{350} \leqslant m_i(0) < \sqrt{500} \rightarrow \{m_i(t) < \sqrt{50}\} \cap T_i^\star > t$	<0.01/<0.01/96	<0.01/<0.01/95	<0.01/<0.01/94.8	<0.01/<0.01/95.2	<0.01/<0.01/94.8	<0.01/<0.01/95.4	<0.01/<0.01/94.8	<0.01/<0.01/94.4	<0.01/<0.01/94	<0.01/<0.01/92.4
$\sqrt{350} \leqslant m_i(0) < \sqrt{500} \rightarrow \{\sqrt{50} \leqslant m_i(t) < \sqrt{100}\} \cap T_i^\star > t$	<0.01/<0.01/96	0.01/0.01/95	0.04/0.04/94.8	0.03/0.03/94.8	0.02/0.02/95	<0.01/<0.01/95.4	0.01/0.01/94.6	0.04/0.04/95	0.03/0.03/93.6	0.02/0.02/94.4
$\sqrt{350} \leqslant m_i(0) < \sqrt{500} \rightarrow \{\sqrt{100} \leqslant m_i(t) < \sqrt{200}\} \cap T_i^\star > t$	0.06/0.07/95.6	0.38/0.38/95.6	0.65/0.66/95.4	0.52/0.53/95.6	0.42/0.43/95.4	0.06/0.07/95.2	0.38/0.38/95.2	0.65/0.65/95	0.52/0.51/94.2	0.42/0.41/93.8
$\sqrt{350} \leqslant m_i(0) < \sqrt{500} \rightarrow \{\sqrt{200} \leqslant m_i(t) < \sqrt{250}\} \cap T_i^\star > t$	0.25/0.26/95.6	0.76/0.76/94.8	1/1.01/95	0.83/0.84/94.8	0.71/0.71/95.2	0.25/0.26/95	0.76/0.76/95	1/0.99/93.6	0.83/0.82/94.4	0.71/0.69/93.6
$\sqrt{350} \leqslant m_i(0) < \sqrt{500} \rightarrow \{\sqrt{250} \leqslant m_i(t) < \sqrt{350}\} \cap T_i^\star > t$	2.69/2.7/95.4	4.19/4.17/94.6	4.39/4.37/94.2	3.76/3.75/94	3.28/3.28/95.4	2.69/2.7/94.4	4.19/4.16/94.4	4.39/4.34/93.8	3.76/3.7/94.4	3.28/3.23/95
$\sqrt{350} \leqslant m_i(0) < \sqrt{500} \rightarrow \{\sqrt{350} \leqslant m_i(t) < \sqrt{500}\} \cap T_i^\star > t$	17.2/17.17/94.2	15.37/15.27/95.4	13.16/13.06/93.2	11.6/11.51/92.2	10.39/10.31/93.4	17.2/17.21/94.8	15.37/15.28/94.8	13.16/13.03/94	11.6/11.45/92.8	10.39/10.25/93.6
$\sqrt{350} \leqslant m_i(0) < \sqrt{500} \rightarrow \{\sqrt{500} \leqslant m_i(t)\} \cap T_i^\star > t$	65.81/65.83/94.6	54.5/54.66/96.2	47.27/47.46/95.2	42.92/43.07/95.2	39.5/39.59/96	65.81/66.03/94.8	54.5/54.82/94.8	47.27/47.55/95.4	42.92/43.09/95.2	39.5/39.55/95.6
$\sqrt{500} \leqslant m_i(0) \rightarrow \{m_i(t) < \sqrt{50}\} \cap T_i^\star > t$	<0.01/<0.01/95.6	<0.01/<0.01/94.6	<0.01/<0.01/94	<0.01/<0.01/94.6	<0.01/<0.01/94.6	<0.01/<0.01/95.4	<0.01/<0.01/94.8	<0.01/<0.01/95	<0.01/<0.01/93	<0.01/<0.01/93
$\sqrt{500} \leqslant m_i(0) \rightarrow \{\sqrt{50} \leqslant m_i(t) < \sqrt{100}\} \cap T_i^\star > t$	<0.01/<0.01/95.4	<0.01/<0.01/95.2	0.02/0.02/94.4	0.01/0.01/95	0.01/0.01/95.6	<0.01/<0.01/95	<0.01/<0.01/94.8	0.02/0.02/95	0.01/0.01/94	0.01/0.01/93.2
$\sqrt{500} \leqslant m_i(0) \rightarrow \{\sqrt{100} \leqslant m_i(t) < \sqrt{200}\} \cap T_i^\star > t$	<0.01/<0.01/95.4	0.11/0.11/94.8	0.3/0.31/94.8	0.24/0.25/94.4	0.2/0.21/94.8	<0.01/<0.01/94.4	0.11/0.11/95	0.3/0.3/94.8	0.24/0.24/94.4	0.2/0.19/94.6
$\sqrt{500} \leqslant m_i(0) \rightarrow \{\sqrt{200} \leqslant m_i(t) < \sqrt{250}\} \cap T_i^\star > t$	0.02/0.02/94.8	0.26/0.26/95	0.53/0.54/95	0.45/0.45/95	0.38/0.38/94.6	0.02/0.02/94.4	0.26/0.26/94.4	0.53/0.53/94.2	0.45/0.44/93.8	0.38/0.37/94
$\sqrt{500} \leqslant m_i(0) \rightarrow \{\sqrt{250} \leqslant m_i(t) < \sqrt{350}\} \cap T_i^\star > t$	0.4/0.41/95.2	1.83/1.82/94.4	2.7/2.69/95	2.33/2.32/94.4	2.04/2.04/94.2	0.4/0.41/93.8	1.83/1.82/94.6	2.7/2.67/94.6	2.33/2.28/93.6	2.04/2/93.4
$\sqrt{500} \leqslant m_i(0) \rightarrow \{\sqrt{350} \leqslant m_i(t) < \sqrt{500}\} \cap T_i^\star > t$	5.29/5.3/94	9.33/9.23/95	9.92/9.8/94.8	8.77/8.66/93.6	7.88/7.79/93.8	5.29/5.31/94.8	9.33/9.23/95.2	9.92/9.76/93.8	8.77/8.6/93.8	7.88/7.72/94
$\sqrt{500} \leqslant m_i(0) \rightarrow \{\sqrt{500} \leqslant m_i(t)\} \cap T_i^\star > t$	81.92/81.88/94.6	66.09/66.22/94.6	55.83/56.01/94	50.88/51.02/94.8	46.97/47.06/95.6	81.92/82.19/94	66.09/66.45/94.6	55.83/56.11/95.4	50.88/50.99/94.6	46.97/46.94/95

† Results from 500 replications with each dataset including 2000 individuals. The "true" marker evolution was based on linear splines with knots at 1 and 5 years since baseline and it was correctly specified in the fitted SPM-1 and SPM-2 models. The true parameter values, the mean of posterior medians over the 500 replications and the empirical coverage probability (%) of posterior credible intervals are presented, respectively.

Table S7. Simulation study results for transition marker state probabilities (%) by baseline marker state for group 1 ($w = 1$) when the data are simulated by SPM-2 and there is misclassification†.

transition	$t=2$	$t=4$	$t=6$	$t=8$	$t=10$	$t=2$	$t=4$	$t=6$	$t=8$	$t=10$
	Results from SPM-1					Results from SPM-2				
$m_i(0) < \sqrt{50} \to \{m_i(t) < \sqrt{50}\} \cap T_i^\star > t$	1.43/1.45/93.8	0.23/0.22/95.2	0.08/0.08/95	0.03/0.02/92.2	<0.01/0.01/91.8	1.43/1.44/95.4	0.23/0.24/95.8	0.08/0.09/94.8	0.03/0.03/94.4	<0.01/0.01/93.8
$m_i(0) < \sqrt{50} \to \{\sqrt{50} \leqslant m_i(t) < \sqrt{100}\} \cap T_i^\star > t$	5.95/6.07/93.8	1.26/1.29/94	0.53/0.53/94.2	0.3/0.29/94.6	0.15/0.14/93	5.95/5.97/95	1.26/1.31/95.6	0.53/0.56/95.4	0.3/0.33/93.6	0.15/0.18/93.6
$m_i(0) < \sqrt{50} \to \{\sqrt{100} \leqslant m_i(t) < \sqrt{200}\} \cap T_i^\star > t$	24.39/24.66/93.2	8.11/8.27/95	3.94/3.99/95	2.9/2.88/94.2	2.14/2.09/93.8	24.39/24.33/94	8.11/8.22/95.6	3.94/4.05/94.4	2.9/2.98/94.2	2.14/2.22/93.6
$m_i(0) < \sqrt{50} \to \{\sqrt{200} \leqslant m_i(t) < \sqrt{250}\} \cap T_i^\star > t$	12.33/12.38/93.8	6.5/6.58/93.8	3.61/3.64/94	2.88/2.88/93.2	2.34/2.32/94.4	12.33/12.25/94	6.5/6.52/94	3.61/3.64/95	2.88/2.91/94.6	2.34/2.36/94.2
$m_i(0) < \sqrt{50} \to \{\sqrt{250} \leqslant m_i(t) < \sqrt{350}\} \cap T_i^\star > t$	15.39/15.39/95	13.94/13.99/93.8	9.18/9.2/93.2	7.66/7.64/93.8	6.52/6.46/94.4	15.39/15.24/94.8	13.94/13.86/94.4	9.18/9.17/94	7.66/7.65/94	6.52/6.5/94
$m_i(0) < \sqrt{50} \to \{\sqrt{350} \leqslant m_i(t) < \sqrt{500}\} \cap T_i^\star > t$	6.17/6.17/95	14.49/14.42/93.8	13.05/12.96/93.6	11.33/11.22/93	10.01/9.88/92.4	6.17/6.11/93.6	14.49/14.3/94.8	13.05/12.89/93.8	11.33/11.18/93	10.01/9.87/92.8
$m_i(0) < \sqrt{50} \to \{\sqrt{500} \leqslant m_i(t)\} \cap T_i^\star > t$	0.65/0.66/94.2	7.38/7.34/94.6	12.55/12.46/94	11.27/11.17/94.6	10.27/10.15/94	0.65/0.65/93	7.38/7.29/95	12.55/12.39/94.4	11.27/11.11/94.2	10.27/10.11/93.6
$\sqrt{50} \leqslant m_i(0) < \sqrt{100} \to \{m_i(t) < \sqrt{50}\} \cap T_i^\star > t$	0.11/0.11/94.8	0.07/0.08/94	0.05/0.06/94.4	0.02/0.02/93.4	0.01/0.01/92.2	0.11/0.11/94.8	0.07/0.07/94.8	0.05/0.06/95	0.02/0.03/93.6	0.01/0.01/92.8
$\sqrt{50} \leqslant m_i(0) < \sqrt{100} \to \{\sqrt{50} \leqslant m_i(t) < \sqrt{100}\} \cap T_i^\star > t$	1.26/1.29/93.2	0.54/0.57/93.6	0.36/0.38/93.4	0.24/0.25/92	0.15/0.16/92.8	1.26/1.27/94.4	0.54/0.56/94.4	0.36/0.38/94.6	0.24/0.25/93.2	0.15/0.16/92.6
$\sqrt{50} \leqslant m_i(0) < \sqrt{100} \to \{\sqrt{100} \leqslant m_i(t) < \sqrt{200}\} \cap T_i^\star > t$	13.66/13.83/94.8	5.18/5.35/91.8	3.12/3.26/91.4	2.43/2.53/92.2	1.92/2.01/91.8	13.66/13.74/95.2	5.18/5.28/94	3.12/3.22/94.4	2.43/2.5/93.6	1.92/1.99/93.4
$\sqrt{50} \leqslant m_i(0) < \sqrt{100} \to \{\sqrt{200} \leqslant m_i(t) < \sqrt{250}\} \cap T_i^\star > t$	12.56/12.63/94	5.36/5.46/92.6	3.25/3.33/92	2.67/2.75/91.4	2.25/2.31/91.6	12.56/12.58/94.4	5.36/5.41/94	3.25/3.3/94.4	2.67/2.72/94.6	2.25/2.29/92.6
$\sqrt{50} \leqslant m_i(0) < \sqrt{100} \to \{\sqrt{250} \leqslant m_i(t) < \sqrt{350}\} \cap T_i^\star > t$	25.07/25.09/94.8	14.47/14.59/93.2	9.39/9.52/92.6	8.01/8.12/93.4	6.97/7.07/93.4	25.07/25.02/94.4	14.47/14.51/93.2	9.39/9.45/93.8	8.01/8.06/94.6	6.97/7.02/94.2
$\sqrt{50} \leqslant m_i(0) < \sqrt{100} \to \{\sqrt{350} \leqslant m_i(t) < \sqrt{500}\} \cap T_i^\star > t$	18.18/18.12/95.6	20.3/20.27/94.8	15.93/15.94/93.2	14.03/14.03/93.2	12.57/12.57/94	18.18/18.08/94.6	20.3/20.21/94.4	15.93/15.88/93	14.03/13.97/93.6	12.57/12.51/93.8
$\sqrt{50} \leqslant m_i(0) < \sqrt{100} \to \{\sqrt{500} \leqslant m_i(t)\} \cap T_i^\star > t$	3.75/3.76/93.4	15.29/15.22/95.6	20.19/20.1/95.2	18.3/18.2/95.6	16.82/16.72/94.8	3.75/3.75/92.6	15.29/15.2/95	20.19/20.08/94.8	18.3/18.18/95.4	16.82/16.69/95.2
$\sqrt{100} \leqslant m_i(0) < \sqrt{200} \to \{m_i(t) < \sqrt{50}\} \cap T_i^\star > t$	0.01/0.01/94	0.02/0.02/94.4	0.03/0.03/93.2	0.01/0.02/92	0.01/0.01/92.2	0.01/0.01/94	0.02/0.02/95	0.03/0.03/94	0.01/0.02/94	0.01/0.01/93.2
$\sqrt{100} \leqslant m_i(0) < \sqrt{200} \to \{\sqrt{50} \leqslant m_i(t) < \sqrt{100}\} \cap T_i^\star > t$	0.22/0.23/95	0.22/0.24/93.6	0.21/0.23/92	0.15/0.16/90.6	0.1/0.11/91.2	0.22/0.23/94.6	0.22/0.23/94.2	0.21/0.22/93.6	0.15/0.16/92.6	0.1/0.11/93.8
$\sqrt{100} \leqslant m_i(0) < \sqrt{200} \to \{\sqrt{100} \leqslant m_i(t) < \sqrt{200}\} \cap T_i^\star > t$	4.86/4.9/94.6	2.88/2.96/93.4	2.2/2.3/90.6	1.74/1.83/90.4	1.41/1.5/89.6	4.86/4.89/93.6	2.88/2.93/94.2	2.2/2.26/93.8	1.74/1.79/94.2	1.41/1.46/93.4
$\sqrt{100} \leqslant m_i(0) < \sqrt{200} \to \{\sqrt{200} \leqslant m_i(t) < \sqrt{250}\} \cap T_i^\star > t$	7.03/7.05/93.2	3.64/3.69/92	2.56/2.63/91.2	2.13/2.2/91.4	1.82/1.89/90.2	7.03/7.05/92.4	3.64/3.67/92.8	2.56/2.6/92.4	2.13/2.17/93.6	1.82/1.85/93.8
$\sqrt{100} \leqslant m_i(0) < \sqrt{200} \to \{\sqrt{250} \leqslant m_i(t) < \sqrt{350}\} \cap T_i^\star > t$	22.35/22.34/93.6	12.05/12.12/93.8	8.34/8.44/93	7.18/7.29/92.2	6.31/6.42/92	22.35/22.37/94.2	12.05/12.09/93.4	8.34/8.39/93.6	7.18/7.22/94.2	6.31/6.35/93.8
$\sqrt{100} \leqslant m_i(0) < \sqrt{200} \to \{\sqrt{350} \leqslant m_i(t) < \sqrt{500}\} \cap T_i^\star > t$	30.61/30.52/94	22.4/22.36/93.2	16.71/16.7/93	14.81/14.82/92.2	13.36/13.39/94.6	30.61/30.58/95	22.4/22.37/92.8	16.71/16.68/93.6	14.81/14.78/93.6	13.36/13.32/93.8
$\sqrt{100} \leqslant m_i(0) < \sqrt{200} \to \{\sqrt{500} \leqslant m_i(t)\} \cap T_i^\star > t$	14.21/14.17/94.4	25.69/25.61/94.8	28.15/28.06/95.2	25.66/25.58/94.8	23.71/23.63/95.6	14.21/14.19/95.2	25.69/25.67/96.2	28.15/28.13/96.2	25.66/25.61/95.8	23.71/23.63/95.4
$\sqrt{200} \leqslant m_i(0) < \sqrt{250} \to \{m_i(t) < \sqrt{50}\} \cap T_i^\star > t$	<0.01/<0.01/93.8	0.01/0.01/94.4	0.01/0.02/92.4	0.01/0.01/92.4	<0.01/0.01/91.4	<0.01/<0.01/93.2	0.01/0.01/94.8	0.01/0.02/94.2	0.01/0.01/94.2	<0.01/<0.01/92.8
$\sqrt{200} \leqslant m_i(0) < \sqrt{250} \to \{\sqrt{50} \leqslant m_i(t) < \sqrt{100}\} \cap T_i^\star > t$	0.03/0.03/94.4	0.09/0.09/95.2	0.12/0.14/91.6	0.09/0.1/92.2	0.06/0.07/90	0.03/0.03/93.8	0.09/0.09/94.8	0.12/0.13/94.2	0.09/0.09/93.6	0.06/0.07/92.4
$\sqrt{200} \leqslant m_i(0) < \sqrt{250} \to \{\sqrt{100} \leqslant m_i(t) < \sqrt{200}\} \cap T_i^\star > t$	1.29/1.31/94.2	1.52/1.56/94.6	1.51/1.58/92.8	1.21/1.28/92.2	1/1.06/90.6	1.29/1.31/94.2	1.52/1.55/95.2	1.51/1.55/92.8	1.21/1.24/93.2	1/1.03/93.2
$\sqrt{200} \leqslant m_i(0) < \sqrt{250} \to \{\sqrt{200} \leqslant m_i(t) < \sqrt{250}\} \cap T_i^\star > t$	2.88/2.88/93.8	2.31/2.33/93.6	1.95/2/92.8	1.64/1.69/91.8	1.41/1.46/92	2.88/2.88/94.4	2.31/2.32/94	1.95/1.98/93	1.64/1.66/92.8	1.41/1.43/93.2
$\sqrt{200} \leqslant m_i(0) < \sqrt{250} \to \{\sqrt{250} \leqslant m_i(t) < \sqrt{350}\} \cap T_i^\star > t$	14.34/14.28/94	9.19/9.2/92.6	7.07/7.12/92	6.11/6.19/92.4	5.4/5.5/92.6	14.34/14.33/94	9.19/9.2/93	7.07/7.09/92.8	6.11/6.13/93	5.4/5.42/93.6
$\sqrt{200} \leqslant m_i(0) < \sqrt{250} \to \{\sqrt{350} \leqslant m_i(t) < \sqrt{500}\} \cap T_i^\star > t$	33.97/33.81/93.6	21.77/21.67/94.4	16.32/16.27/93.6	14.52/14.5/93.6	13.15/13.16/93.4	33.97/33.95/94	21.77/21.72/92.8	16.32/16.26/93.6	14.52/14.46/93.4	13.15/13.09/94.4
$\sqrt{200} \leqslant m_i(0) < \sqrt{250} \to \{\sqrt{500} \leqslant m_i(t)\} \cap T_i^\star > t$	29.69/29.6/96.4	35.72/35.61/95	35.13/35.05/94.8	32.12/32.06/95.6	29.76/29.72/95.4	29.69/29.72/95.8	35.72/35.76/95.6	35.13/35.17/95.6	32.12/32.12/95.2	29.76/29.73/95.2
$\sqrt{250} \leqslant m_i(0) < \sqrt{350} \to \{m_i(t) < \sqrt{50}\} \cap T_i^\star > t$	<0.01/<0.01/93.6	<0.01/<0.01/94	0.01/0.01/93.2	0.01/0.01/92.4	<0.01/<0.01/91.4	<0.01/<0.01/93	<0.01/<0.01/94.4	0.01/0.01/95	0.01/0.01/94.8	<0.01/<0.01/92.2
$\sqrt{250} \leqslant m_i(0) < \sqrt{350} \to \{\sqrt{50} \leqslant m_i(t) < \sqrt{100}\} \cap T_i^\star > t$	0.01/0.01/93.8	0.04/0.04/94.4	0.08/0.09/92.8	0.06/0.06/92.4	0.04/0.05/90.6	0.01/0.01/94	0.04/0.04/94.8	0.08/0.08/94.8	0.06/0.06/93.6	0.04/0.04/92.8
$\sqrt{250} \leqslant m_i(0) < \sqrt{350} \to \{\sqrt{100} \leqslant m_i(t) < \sqrt{200}\} \cap T_i^\star > t$	0.42/0.43/93.8	0.89/0.91/95	1.09/1.14/94	0.88/0.93/93	0.73/0.78/91.2	0.42/0.43/94.2	0.89/0.9/94.8	1.09/1.12/94.2	0.88/0.9/94.2	0.73/0.75/93.4
$\sqrt{250} \leqslant m_i(0) < \sqrt{350} \to \{\sqrt{200} \leqslant m_i(t) < \sqrt{250}\} \cap T_i^\star > t$	1.2/1.2/93.2	1.52/1.53/95.4	1.52/1.55/93.2	1.28/1.32/93	1.11/1.15/91.4	1.2/1.2/93.4	1.52/1.53/94	1.52/1.53/93.2	1.28/1.29/94.4	1.11/1.12/93
$\sqrt{250} \leqslant m_i(0) < \sqrt{350} \to \{\sqrt{250} \leqslant m_i(t) < \sqrt{350}\} \cap T_i^\star > t$	8.1/8.05/93.8	6.91/6.9/94.2	5.95/5.98/93.4	5.17/5.22/93	4.58/4.65/92.2	8.1/8.08/94.4	6.91/6.9/93.2	5.95/5.95/92.8	5.17/5.16/94	4.58/4.58/93.4
$\sqrt{250} \leqslant m_i(0) < \sqrt{350} \to \{\sqrt{350} \leqslant m_i(t) < \sqrt{500}\} \cap T_i^\star > t$	28.95/28.73/94.2	19.65/19.48/93.6	15.33/15.23/93.6	13.67/13.61/94.2	12.4/12.38/94.4	28.95/28.86/96	19.65/19.55/94.6	15.33/15.23/92.8	13.67/13.57/94	12.4/12.31/94.2
$\sqrt{250} \leqslant m_i(0) < \sqrt{350} \to \{\sqrt{500} \leqslant m_i(t)\} \cap T_i^\star > t$	45.21/45.05/94.6	43.83/43.68/94.4	40.55/40.46/93.8	37.15/37.1/95.2	34.5/34.46/95.4	45.21/45.27/95	43.83/43.91/95	40.55/40.63/94.6	37.15/37.18/95.2	34.5/34.48/95.4
$\sqrt{350} \leqslant m_i(0) < \sqrt{500} \to \{m_i(t) < \sqrt{50}\} \cap T_i^\star > t$	<0.01/<0.01/93.2	<0.01/<0.01/94	<0.01/<0.01/94.4	<0.01/<0.01/93.4	<0.01/<0.01/92	<0.01/<0.01/93.2	<0.01/<0.01/94.2	<0.01/<0.01/95.8	<0.01/<0.01/94.4	<0.01/<0.01/93.4
$\sqrt{350} \leqslant m_i(0) < \sqrt{500} \to \{\sqrt{50} \leqslant m_i(t) < \sqrt{100}\} \cap T_i^\star > t$	<0.01/<0.01/93.4	0.01/0.01/94	0.04/0.04/94.8	0.03/0.03/94.2	0.02/0.03/92.4	<0.01/<0.01/93.2	0.01/0.01/93.6	0.04/0.04/95	0.03/0.03/94.6	0.02/0.02/94.4
$\sqrt{350} \leqslant m_i(0) < \sqrt{500} \to \{\sqrt{100} \leqslant m_i(t) < \sqrt{200}\} \cap T_i^\star > t$	0.06/0.06/93.8	0.37/0.38/95	0.65/0.68/95.2	0.53/0.56/94.4	0.44/0.47/93.8	0.06/0.06/93.4	0.37/0.38/94.6	0.65/0.67/94.4	0.53/0.54/94.8	0.44/0.45/93.8
$\sqrt{350} \leqslant m_i(0) < \sqrt{500} \to \{\sqrt{200} \leqslant m_i(t) < \sqrt{250}\} \cap T_i^\star > t$	0.25/0.25/92.8	0.76/0.76/94.4	1.01/1.03/94.8	0.85/0.88/94.4	0.74/0.77/94	0.25/0.26/92.8	0.76/0.76/93.8	1.01/1.01/93.8	0.85/0.86/93.4	0.74/0.74/94
$\sqrt{350} \leqslant m_i(0) < \sqrt{500} \to \{\sqrt{250} \leqslant m_i(t) < \sqrt{350}\} \cap T_i^\star > t$	2.67/2.65/93.2	4.18/4.15/94	4.42/4.42/94.2	3.85/3.87/94.8	3.42/3.46/94	2.67/2.66/93	4.18/4.15/93.8	4.42/4.4/94	3.85/3.83/93	3.42/3.41/92.6
$\sqrt{350} \leqslant m_i(0) < \sqrt{500} \to \{\sqrt{350} \leqslant m_i(t) < \sqrt{500}\} \cap T_i^\star > t$	17.12/16.92/94.8	15.38/15.17/92.8	13.31/13.15/92.2	11.89/11.79/92.2	10.81/10.75/92.8	17.12/17/94.4	15.38/15.22/93	13.31/13.16/92.2	11.89/11.74/91.6	10.81/10.67/92.8
$\sqrt{350} \leqslant m_i(0) < \sqrt{500} \to \{\sqrt{500} \leqslant m_i(t)\} \cap T_i^\star > t$	65.65/65.31/93.8	54.69/54.44/94.2	47.91/47.77/93.4	44/43.92/94.2	40.96/40.92/94.6	65.65/65.67/94.2	54.69/54.77/94.2	47.91/47.99/94.2	44/44.03/94.2	40.96/40.91/95.2
$\sqrt{500} \leqslant m_i(0) \to \{m_i(t) < \sqrt{50}\} \cap T_i^\star > t$	<0.01/<0.01/93.4	<0.01/<0.01/95	<0.01/<0.01/95	<0.01/<0.01/94.2	<0.01/<0.01/94	<0.01/<0.01/93.6	<0.01/<0.01/94.2	<0.01/<0.01/95	<0.01/<0.01/94.4	<0.01/<0.01/93.4
$\sqrt{500} \leqslant m_i(0) \to \{\sqrt{50} \leqslant m_i(t) < \sqrt{100}\} \cap T_i^\star > t$	<0.01/<0.01/93.8	<0.01/<0.01/94.8	0.02/0.02/95	0.01/0.01/94.8	0.01/0.01/94.2	<0.01/<0.01/93.6	<0.01/<0.01/93.8	0.02/0.02/95	0.01/0.01/94.4	0.01/0.01/93.6
$\sqrt{500} \leqslant m_i(0) \to \{\sqrt{100} \leqslant m_i(t) < \sqrt{200}\} \cap T_i^\star > t$	<0.01/<0.01/93.4	0.1/0.11/94.4	0.31/0.32/94.4	0.25/0.26/94.8	0.21/0.23/94.4	<0.01/<0.01/93	0.1/0.11/94	0.31/0.31/94	0.25/0.25/93.6	0.21/0.22/94
$\sqrt{500} \leqslant m_i(0) \to \{\sqrt{200} \leqslant m_i(t) < \sqrt{250}\} \cap T_i^\star > t$	0.02/0.02/93.4	0.26/0.26/94	0.54/0.55/94.2	0.46/0.47/94.4	0.4/0.41/94.8	0.02/0.02/92.4	0.26/0.26/93.8	0.54/0.54/94	0.46/0.46/93.4	0.4/0.4/93.2
$\sqrt{500} \leqslant m_i(0) \to \{\sqrt{250} \leqslant m_i(t) < \sqrt{350}\} \cap T_i^\star > t$	0.39/0.4/93.2	1.83/1.81/94.2	2.73/2.72/94.8	2.38/2.39/94.6	2.12/2.14/94.8	0.39/0.4/93	1.83/1.81/93.8	2.73/2.7/93.4	2.38/2.35/93	2.12/2.1/93.4
$\sqrt{500} \leqslant m_i(0) \to \{\sqrt{350} \leqslant m_i(t) < \sqrt{500}\} \cap T_i^\star > t$	5.27/5.21/95	9.35/9.15/93.4	10.04/9.86/93.2	8.99/8.86/93.6	8.19/8.1/92.6	5.27/5.23/94	9.35/9.18/94	10.04/9.85/93.8	8.99/8.81/93	8.19/8.02/92.6
$\sqrt{500} \leqslant m_i(0) \to \{\sqrt{500} \leqslant m_i(t)\} \cap T_i^\star > t$	81.83/81.21/91.8	66.4/65.93/93.4	56.6/56.35/93.8	52.13/51.98/94.8	48.63/48.56/94.6	81.83/81.68/93	66.4/66.34/93.8	56.6/56.59/94.2	52.13/52.06/94.2	48.63/48.49/94.2

† Results from 500 replications with each dataset including 2000 individuals. The "true" marker evolution was based on linear splines with knots at 1 and 5 years since baseline and it was correctly specified in the fitted SPM-1 and SPM-2 models. The true parameter values, the mean of posterior medians over the 500 replications and the empirical coverage probability (%) of posterior credible intervals are presented, respectively.

Table S8. Simulation study results for transition marker state probabilities (%) by baseline marker state for group 0 ($w = 0$) when the data are simulated by SPM-2 and there is misclassification†.

transition	$t = 2$	$t = 4$	$t = 6$	$t = 8$	$t = 10$	$t = 2$	$t = 4$	$t = 6$	$t = 8$	$t = 10$
	Results from SPM-1					Results from SPM-2				
$m_i(0) < \sqrt{50} \rightarrow \{m_i(t) < \sqrt{50}\} \cap T_i^\star > t$	1.47/1.51/94.4	0.23/0.24/95.4	0.08/0.08/95.2	0.03/0.03/93.8	<0.01/0.01/94	1.47/1.49/94.4	0.23/0.25/96	0.08/0.09/95.2	0.03/0.03/94.2	<0.01/0.01/94
$m_i(0) < \sqrt{50} \rightarrow \{\sqrt{50} \leqslant m_i(t) < \sqrt{100}\} \cap T_i^\star > t$	6.04/6.21/94.8	1.28/1.34/95.4	0.54/0.56/94.4	0.31/0.3/95.2	0.15/0.15/93.4	6.04/6.08/95.8	1.28/1.33/95.8	0.54/0.57/93.8	0.31/0.33/94.4	0.15/0.18/93.8
$m_i(0) < \sqrt{50} \rightarrow \{\sqrt{100} \leqslant m_i(t) < \sqrt{200}\} \cap T_i^\star > t$	24.53/24.91/91.2	8.15/8.4/93.8	3.94/4.06/93.4	2.89/2.92/93.6	2.12/2.11/94.8	24.53/24.53/93.6	8.15/8.29/95.6	3.94/4.07/93.2	2.89/2.98/93.4	2.12/2.21/93.6
$m_i(0) < \sqrt{50} \rightarrow \{\sqrt{200} \leqslant m_i(t) < \sqrt{250}\} \cap T_i^\star > t$	12.34/12.43/92	6.5/6.62/92	3.59/3.66/92.2	2.85/2.87/93	2.3/2.3/93.6	12.34/12.27/93.6	6.5/6.52/94.4	3.59/3.64/94	2.85/2.89/94.2	2.3/2.34/94.4
$m_i(0) < \sqrt{50} \rightarrow \{\sqrt{250} \leqslant m_i(t) < \sqrt{350}\} \cap T_i^\star > t$	15.36/15.4/94.8	13.87/13.99/93.4	9.09/9.16/91.6	7.54/7.55/92.4	6.38/6.34/93	15.36/15.23/95	13.87/13.81/93.4	9.09/9.1/93.8	7.54/7.55/93.2	6.38/6.39/93.6
$m_i(0) < \sqrt{50} \rightarrow \{\sqrt{350} \leqslant m_i(t) < \sqrt{500}\} \cap T_i^\star > t$	6.14/6.15/94.8	14.34/14.33/94	12.84/12.81/92.4	11.08/11/93.2	9.74/9.61/93.2	6.14/6.08/94.6	14.34/14.17/93.2	12.84/12.71/93	11.08/10.96/93	9.74/9.62/93.6
$m_i(0) < \sqrt{50} \rightarrow \{\sqrt{500} \leqslant m_i(t)\} \cap T_i^\star > t$	0.64/0.65/94.2	7.27/7.26/94.8	12.29/12.24/95.4	10.96/10.87/95.4	9.94/9.81/95.2	0.64/0.65/93.6	7.27/7.19/94	12.29/12.15/95	10.96/10.82/96.2	9.94/9.79/94.8
$\sqrt{50} \leqslant m_i(0) < \sqrt{100} \rightarrow \{m_i(t) < \sqrt{50}\} \cap T_i^\star > t$	0.11/0.12/94.2	0.07/0.08/95	0.05/0.06/94.6	0.02/0.03/93.6	0.01/0.01/93.4	0.11/0.11/95	0.07/0.08/96.4	0.05/0.06/94.8	0.02/0.03/93.8	0.01/0.01/93.8
$\sqrt{50} \leqslant m_i(0) < \sqrt{100} \rightarrow \{\sqrt{50} \leqslant m_i(t) < \sqrt{100}\} \cap T_i^\star > t$	1.26/1.3/93.6	0.54/0.58/94.2	0.36/0.39/93.8	0.24/0.25/93.6	0.15/0.16/93.8	1.26/1.29/94.6	0.54/0.57/95.8	0.36/0.38/93.6	0.24/0.25/93.2	0.15/0.16/93.8
$\sqrt{50} \leqslant m_i(0) < \sqrt{100} \rightarrow \{\sqrt{100} \leqslant m_i(t) < \sqrt{200}\} \cap T_i^\star > t$	13.65/13.86/95.2	5.16/5.35/92.8	3.09/3.25/92.4	2.39/2.51/90.8	1.89/1.98/92.2	13.65/13.75/95.2	5.16/5.27/95	3.09/3.2/93.2	2.39/2.48/93	1.89/1.96/93.6
$\sqrt{50} \leqslant m_i(0) < \sqrt{100} \rightarrow \{\sqrt{200} \leqslant m_i(t) < \sqrt{250}\} \cap T_i^\star > t$	12.51/12.62/92.4	5.31/5.43/91.8	3.2/3.3/91.6	2.62/2.7/92.4	2.19/2.26/93.2	12.51/12.54/92.6	5.31/5.37/94.4	3.2/3.26/93.2	2.62/2.67/93.2	2.19/2.24/93.4
$\sqrt{50} \leqslant m_i(0) < \sqrt{100} \rightarrow \{\sqrt{250} \leqslant m_i(t) < \sqrt{350}\} \cap T_i^\star > t$	24.9/25.01/93.6	14.29/14.45/91	9.22/9.37/92.4	7.82/7.93/92	6.77/6.85/92.6	24.9/24.88/93	14.29/14.34/93.4	9.22/9.29/93	7.82/7.88/93.2	6.77/6.83/93.2
$\sqrt{50} \leqslant m_i(0) < \sqrt{100} \rightarrow \{\sqrt{350} \leqslant m_i(t) < \sqrt{500}\} \cap T_i^\star > t$	18.02/18.02/94.8	19.97/20.01/93.2	15.57/15.61/94	13.63/13.63/94.4	12.14/12.11/94.2	18.02/17.93/95	19.97/19.9/93.6	15.57/15.54/94.6	13.63/13.59/95.4	12.14/12.1/95
$\sqrt{50} \leqslant m_i(0) < \sqrt{100} \rightarrow \{\sqrt{500} \leqslant m_i(t)\} \cap T_i^\star > t$	3.71/3.73/93.6	14.99/14.97/95.2	19.65/19.6/95.8	17.69/17.6/95.4	16.17/16.05/95.4	3.71/3.71/92.8	14.99/14.91/94.8	19.65/19.56/95.4	17.69/17.59/95.8	16.17/16.06/95.4
$\sqrt{100} \leqslant m_i(0) < \sqrt{200} \rightarrow \{m_i(t) < \sqrt{50}\} \cap T_i^\star > t$	0.01/0.01/94.6	0.02/0.03/95	0.03/0.03/93.2	0.01/0.02/93.6	0.01/0.01/92.6	0.01/0.01/94.8	0.02/0.02/95.2	0.03/0.03/94.8	0.01/0.02/93.6	0.01/0.01/94.4
$\sqrt{100} \leqslant m_i(0) < \sqrt{200} \rightarrow \{\sqrt{50} \leqslant m_i(t) < \sqrt{100}\} \cap T_i^\star > t$	0.22/0.23/94.8	0.22/0.24/94.6	0.21/0.23/93.4	0.15/0.16/92.8	0.1/0.11/92	0.22/0.23/94.4	0.22/0.23/96.4	0.21/0.22/94.6	0.15/0.15/94	0.1/0.11/93.2
$\sqrt{100} \leqslant m_i(0) < \sqrt{200} \rightarrow \{\sqrt{100} \leqslant m_i(t) < \sqrt{200}\} \cap T_i^\star > t$	4.84/4.9/95.2	2.85/2.94/94.8	2.16/2.27/92.2	1.71/1.8/92	1.38/1.46/91.8	4.84/4.87/94.4	2.85/2.91/95.4	2.16/2.23/93.8	1.71/1.76/93.8	1.38/1.43/93.6
$\sqrt{100} \leqslant m_i(0) < \sqrt{200} \rightarrow \{\sqrt{200} \leqslant m_i(t) < \sqrt{250}\} \cap T_i^\star > t$	6.98/7.02/94.8	3.59/3.65/94	2.51/2.58/92.2	2.08/2.15/92	1.77/1.83/92	6.98/7.01/94.4	3.59/3.63/94.4	2.51/2.55/94.2	2.08/2.12/94	1.77/1.8/93.4
$\sqrt{100} \leqslant m_i(0) < \sqrt{200} \rightarrow \{\sqrt{250} \leqslant m_i(t) < \sqrt{350}\} \cap T_i^\star > t$	22.15/22.2/93.6	11.85/11.95/94.4	8.15/8.26/92.2	6.97/7.07/92.8	6.1/6.19/92.6	22.15/22.18/92.6	11.85/11.9/94.2	8.15/8.2/93.8	6.97/7.02/94	6.1/6.15/94.8
$\sqrt{100} \leqslant m_i(0) < \sqrt{200} \rightarrow \{\sqrt{350} \leqslant m_i(t) < \sqrt{500}\} \cap T_i^\star > t$	30.27/30.27/92.8	21.96/21.98/94.2	16.26/16.28/93.8	14.32/14.33/94.4	12.85/12.84/93.6	30.27/30.25/93.4	21.96/21.94/94.6	16.26/16.24/94.4	14.32/14.3/94.4	12.85/12.82/94.2
$\sqrt{100} \leqslant m_i(0) < \sqrt{200} \rightarrow \{\sqrt{500} \leqslant m_i(t)\} \cap T_i^\star > t$	14.03/14.03/95	25.11/25.11/94.8	27.3/27.27/94.2	24.71/24.64/95.4	22.71/22.6/95.4	14.03/14.02/94.4	25.11/25.1/95.2	27.3/27.28/94	24.71/24.67/94.8	22.71/22.64/95
$\sqrt{200} \leqslant m_i(0) < \sqrt{250} \rightarrow \{m_i(t) < \sqrt{50}\} \cap T_i^\star > t$	<0.01/<0.01/93.8	0.01/0.01/94.8	0.01/0.02/93	0.01/0.01/93.2	<0.01/0.01/92.8	<0.01/<0.01/93.6	0.01/0.01/94.8	0.01/0.02/95	0.01/0.01/94	<0.01/<0.01/94.4
$\sqrt{200} \leqslant m_i(0) < \sqrt{250} \rightarrow \{\sqrt{50} \leqslant m_i(t) < \sqrt{100}\} \cap T_i^\star > t$	0.03/0.03/93.8	0.09/0.09/94.8	0.12/0.14/93.4	0.09/0.1/92.4	0.06/0.07/92	0.03/0.03/94.2	0.09/0.09/95.4	0.12/0.13/95.2	0.09/0.09/94	0.06/0.07/94.8
$\sqrt{200} \leqslant m_i(0) < \sqrt{250} \rightarrow \{\sqrt{100} \leqslant m_i(t) < \sqrt{200}\} \cap T_i^\star > t$	1.29/1.3/94	1.5/1.55/95.6	1.48/1.55/93.4	1.19/1.25/93	0.97/1.03/92.6	1.29/1.3/94	1.5/1.53/95.6	1.48/1.52/95.2	1.19/1.22/94.8	0.97/1/95
$\sqrt{200} \leqslant m_i(0) < \sqrt{250} \rightarrow \{\sqrt{200} \leqslant m_i(t) < \sqrt{250}\} \cap T_i^\star > t$	2.85/2.86/94.8	2.27/2.3/95.2	1.91/1.95/94.6	1.59/1.64/93.2	1.36/1.41/92.8	2.85/2.86/94	2.27/2.29/96.2	1.91/1.93/95.2	1.59/1.61/94.2	1.36/1.38/94
$\sqrt{200} \leqslant m_i(0) < \sqrt{250} \rightarrow \{\sqrt{250} \leqslant m_i(t) < \sqrt{350}\} \cap T_i^\star > t$	14.18/14.16/95	9.01/9.05/95.2	6.88/6.95/94	5.92/5.99/94.2	5.21/5.27/94.2	14.18/14.18/95	9.01/9.02/95.6	6.88/6.9/94.6	5.92/5.94/94.8	5.21/5.23/94.4
$\sqrt{200} \leqslant m_i(0) < \sqrt{250} \rightarrow \{\sqrt{350} \leqslant m_i(t) < \sqrt{500}\} \cap T_i^\star > t$	33.55/33.49/92.8	21.29/21.25/94	15.84/15.81/93.4	14/13.98/93	12.61/12.59/93	33.55/33.54/92.6	21.29/21.25/93.8	15.84/15.79/93.6	14/13.95/93.2	12.61/12.56/93.4
$\sqrt{200} \leqslant m_i(0) < \sqrt{250} \rightarrow \{\sqrt{500} \leqslant m_i(t)\} \cap T_i^\star > t$	29.28/29.28/96.4	34.86/34.86/94	34/33.99/93.2	30.87/30.83/94.4	28.45/28.38/94.4	29.28/29.31/96.8	34.86/34.9/94	34/34.04/93.4	30.87/30.88/93.2	28.45/28.42/94
$\sqrt{250} \leqslant m_i(0) < \sqrt{350} \rightarrow \{m_i(t) < \sqrt{50}\} \cap T_i^\star > t$	<0.01/<0.01/94	<0.01/<0.01/94.6	0.01/0.01/93.8	0.01/0.01/93.8	<0.01/<0.01/93.8	<0.01/<0.01/93.2	<0.01/<0.01/95	0.01/0.01/95	0.01/0.01/94	<0.01/<0.01/95.2
$\sqrt{250} \leqslant m_i(0) < \sqrt{350} \rightarrow \{\sqrt{50} \leqslant m_i(t) < \sqrt{100}\} \cap T_i^\star > t$	0.01/0.01/93.8	0.04/0.04/95.2	0.08/0.09/94.4	0.06/0.06/92.6	0.04/0.05/93.2	0.01/0.01/94.2	0.04/0.04/95	0.08/0.08/94.8	0.06/0.06/94	0.04/0.04/94.6
$\sqrt{250} \leqslant m_i(0) < \sqrt{350} \rightarrow \{\sqrt{100} \leqslant m_i(t) < \sqrt{200}\} \cap T_i^\star > t$	0.42/0.43/94	0.88/0.9/95.2	1.07/1.12/93.4	0.86/0.91/93.6	0.71/0.76/93.2	0.42/0.42/94	0.88/0.89/95.4	1.07/1.1/95.6	0.86/0.88/94.6	0.71/0.73/93.8
$\sqrt{250} \leqslant m_i(0) < \sqrt{350} \rightarrow \{\sqrt{200} \leqslant m_i(t) < \sqrt{250}\} \cap T_i^\star > t$	1.18/1.19/93.6	1.49/1.51/95.6	1.48/1.51/94.4	1.24/1.28/92.6	1.07/1.1/92.4	1.18/1.19/93.2	1.49/1.5/94.8	1.48/1.5/95	1.24/1.25/95.2	1.07/1.08/93.8
$\sqrt{250} \leqslant m_i(0) < \sqrt{350} \rightarrow \{\sqrt{250} \leqslant m_i(t) < \sqrt{350}\} \cap T_i^\star > t$	8.01/7.98/94.4	6.77/6.77/94.4	5.79/5.82/94.2	4.99/5.04/94.4	4.41/4.45/93.8	8.01/7.99/93.8	6.77/6.76/94.2	5.79/5.79/94	4.99/4.99/94.6	4.41/4.41/94.6
$\sqrt{250} \leqslant m_i(0) < \sqrt{350} \rightarrow \{\sqrt{350} \leqslant m_i(t) < \sqrt{500}\} \cap T_i^\star > t$	28.57/28.43/95.4	19.19/19.08/94.2	14.86/14.79/93.8	13.16/13.11/93.4	11.88/11.83/92.8	28.57/28.49/95.8	19.19/19.09/94.2	14.86/14.76/92.8	13.16/13.07/93.6	11.88/11.79/93.6
$\sqrt{250} \leqslant m_i(0) < \sqrt{350} \rightarrow \{\sqrt{500} \leqslant m_i(t)\} \cap T_i^\star > t$	44.56/44.54/94.2	42.73/42.72/93.4	39.2/39.2/94.2	35.67/35.65/94	32.93/32.88/93.8	44.56/44.63/94	42.73/42.81/93.8	39.2/39.27/94.4	35.67/35.69/93.8	32.93/32.9/93.2
$\sqrt{350} \leqslant m_i(0) < \sqrt{500} \rightarrow \{m_i(t) < \sqrt{50}\} \cap T_i^\star > t$	<0.01/<0.01/93.2	<0.01/<0.01/94.2	<0.01/<0.01/94.4	<0.01/<0.01/93.8	<0.01/<0.01/93.8	<0.01/<0.01/93.4	<0.01/<0.01/95	<0.01/<0.01/94.6	<0.01/<0.01/94.4	<0.01/<0.01/94.4
$\sqrt{350} \leqslant m_i(0) < \sqrt{500} \rightarrow \{\sqrt{50} \leqslant m_i(t) < \sqrt{100}\} \cap T_i^\star > t$	<0.01/<0.01/93.6	0.01/0.01/94.4	0.04/0.04/94.8	0.03/0.03/93	0.02/0.03/92.4	<0.01/<0.01/93.4	0.01/0.01/94.8	0.04/0.04/94.6	0.03/0.03/94.8	0.02/0.02/94.2
$\sqrt{350} \leqslant m_i(0) < \sqrt{500} \rightarrow \{\sqrt{100} \leqslant m_i(t) < \sqrt{200}\} \cap T_i^\star > t$	0.06/0.06/93.6	0.37/0.38/94.6	0.64/0.66/95.6	0.52/0.54/95	0.43/0.46/94.2	0.06/0.06/93.4	0.37/0.37/94.4	0.64/0.65/95.6	0.52/0.53/95	0.43/0.44/95
$\sqrt{350} \leqslant m_i(0) < \sqrt{500} \rightarrow \{\sqrt{200} \leqslant m_i(t) < \sqrt{250}\} \cap T_i^\star > t$	0.25/0.25/93.4	0.74/0.75/95	0.98/1/95	0.83/0.85/94.6	0.71/0.74/93.6	0.25/0.25/93.2	0.74/0.74/94.2	0.98/0.99/95.4	0.83/0.83/95.4	0.71/0.72/94.8
$\sqrt{350} \leqslant m_i(0) < \sqrt{500} \rightarrow \{\sqrt{250} \leqslant m_i(t) < \sqrt{350}\} \cap T_i^\star > t$	2.63/2.63/94	4.08/4.06/95.6	4.29/4.29/94.6	3.71/3.73/94	3.29/3.31/93.4	2.63/2.63/93.4	4.08/4.06/95.8	4.29/4.27/94.6	3.71/3.69/94	3.29/3.27/94.6
$\sqrt{350} \leqslant m_i(0) < \sqrt{500} \rightarrow \{\sqrt{350} \leqslant m_i(t) < \sqrt{500}\} \cap T_i^\star > t$	16.88/16.73/94.8	15/14.83/94.8	12.88/12.75/93.2	11.43/11.33/94.4	10.34/10.26/93	16.88/16.77/94.8	15/14.84/93.8	12.88/12.73/93.4	11.43/11.29/93.6	10.34/10.2/92.6
$\sqrt{350} \leqslant m_i(0) < \sqrt{500} \rightarrow \{\sqrt{500} \leqslant m_i(t)\} \cap T_i^\star > t$	64.67/64.51/92	53.28/53.2/94.6	46.26/46.25/94.2	42.19/42.18/94	39.05/39.01/93.6	64.67/64.7/92.2	53.28/53.34/94	46.26/46.33/94.4	42.19/42.21/94.2	39.05/39/93.4
$\sqrt{500} \leqslant m_i(0) \rightarrow \{m_i(t) < \sqrt{50}\} \cap T_i^\star > t$	<0.01/<0.01/93.4	<0.01/<0.01/94.6	<0.01/<0.01/94.8	<0.01/<0.01/94.4	<0.01/<0.01/93.6	<0.01/<0.01/93.4	<0.01/<0.01/94.4	<0.01/<0.01/95	<0.01/<0.01/95	<0.01/<0.01/93.8
$\sqrt{500} \leqslant m_i(0) \rightarrow \{\sqrt{50} \leqslant m_i(t) < \sqrt{100}\} \cap T_i^\star > t$	<0.01/<0.01/94	<0.01/<0.01/94.2	0.01/0.02/95	0.01/0.01/94.8	0.01/0.01/94	<0.01/<0.01/93.6	<0.01/<0.01/94.4	0.01/0.02/94.6	0.01/0.01/95	0.01/0.01/94.6
$\sqrt{500} \leqslant m_i(0) \rightarrow \{\sqrt{100} \leqslant m_i(t) < \sqrt{200}\} \cap T_i^\star > t$	<0.01/<0.01/93	0.1/0.11/94.6	0.3/0.31/94.6	0.24/0.26/94.6	0.2/0.22/94.6	<0.01/<0.01/93.2	0.1/0.11/93.8	0.3/0.3/94.6	0.24/0.25/94	0.2/0.21/94.4
$\sqrt{500} \leqslant m_i(0) \rightarrow \{\sqrt{200} \leqslant m_i(t) < \sqrt{250}\} \cap T_i^\star > t$	0.02/0.02/93.4	0.25/0.26/94.2	0.52/0.53/94.8	0.44/0.45/94.8	0.38/0.39/94.4	0.02/0.02/92.8	0.25/0.26/93.6	0.52/0.52/94	0.44/0.44/94.2	0.38/0.38/94.4
$\sqrt{500} \leqslant m_i(0) \rightarrow \{\sqrt{250} \leqslant m_i(t) < \sqrt{350}\} \cap T_i^\star > t$	0.39/0.4/93.6	1.78/1.77/94	2.64/2.63/94.6	2.29/2.29/94.2	2.03/2.04/93.4	0.39/0.4/93.2	1.78/1.77/94	2.64/2.61/93.8	2.29/2.27/93.4	2.03/2.01/94
$\sqrt{500} \leqslant m_i(0) \rightarrow \{\sqrt{350} \leqslant m_i(t) < \sqrt{500}\} \cap T_i^\star > t$	5.19/5.14/95	9.11/8.94/94.6	9.7/9.55/93.4	8.63/8.5/93.6	7.82/7.71/92.8	5.19/5.15/94.4	9.11/8.95/94.4	9.7/9.52/93.4	8.63/8.45/92.8	7.82/7.65/92.8
$\sqrt{500} \leqslant m_i(0) \rightarrow \{\sqrt{500} \leqslant m_i(t)\} \cap T_i^\star > t$	80.59/80.19/92	64.65/64.4/93.4	54.62/54.53/94.2	49.95/49.89/94.2	46.34/46.28/93.4	80.59/80.45/92.4	64.65/64.58/93.8	54.62/54.61/93.8	49.95/49.88/94.6	46.34/46.19/94

† Results from 500 replications with each dataset including 2000 individuals. The “true” marker evolution was based on linear splines with knots at 1 and 5 years since baseline and it was correctly specified in the fitted SPM-1 and SPM-2 models. The true parameter values, the mean of posterior medians over the 500 replications and the empirical coverage probability (%) of posterior credible intervals are presented, respectively.

7.3 *Simulation study results for population-averaged CIFs by baseline marker state*

Results for population-averaged CIFs by baseline marker state are provided in Figures S3-S6. Both models, even when the fitted model was misspecified, yielded almost unbiased estimates with satisfactory coverage rates. However, there were few cases where the coverage probabilities from the misspecified model were lower than the nominal level. For example, when data were generated from the SPM-1 model, the coverage probability for the population-averaged CIF of cause 1, conditional on the $m_i(0) > \sqrt{500}$ initial state based on the SPM-2 model, reduced to 87.8% (Figure S4).

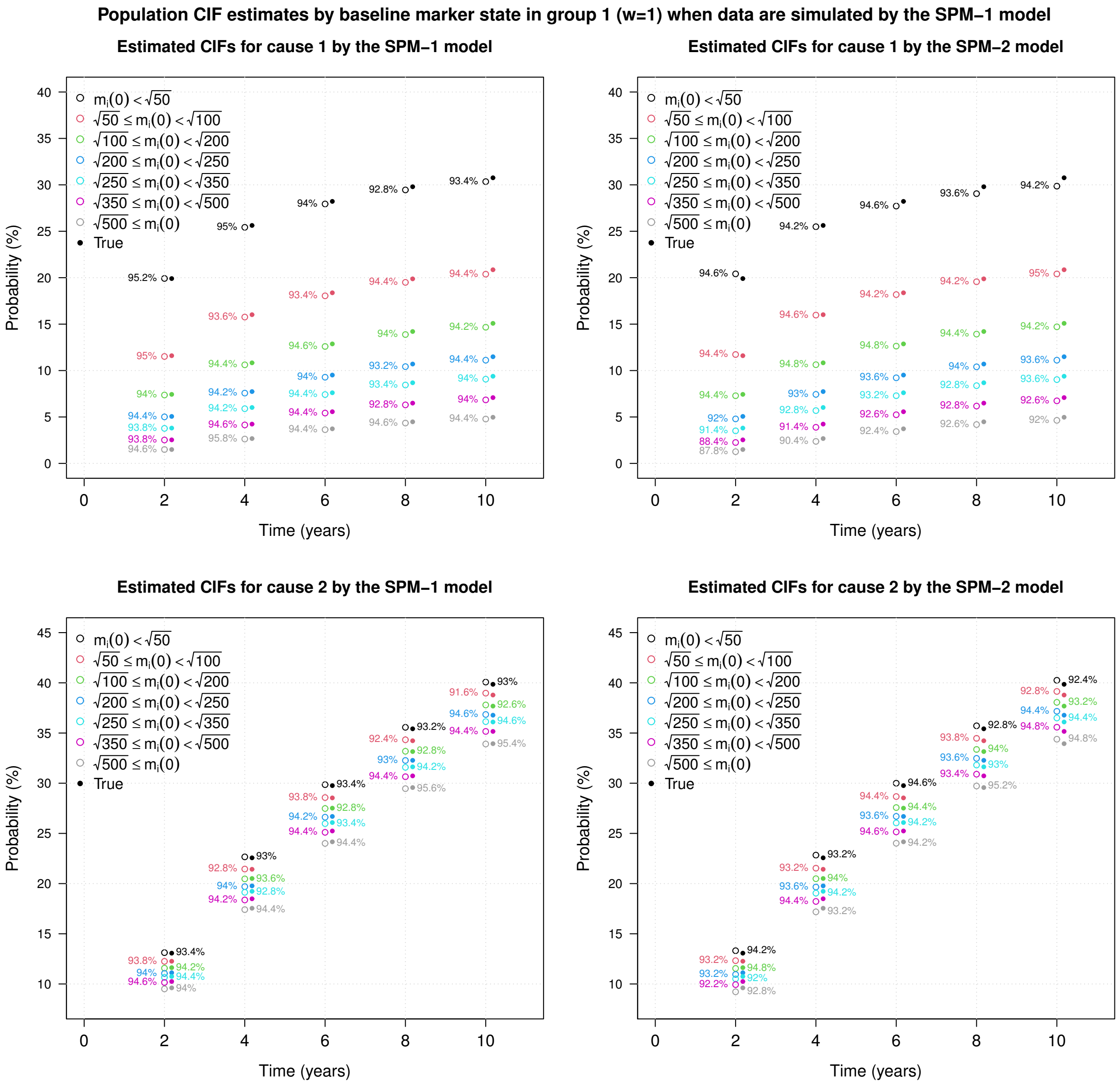

Figure S3. Simulation study results for population-averaged CIF estimates by baseline "true" marker state for group 1 when data are simulated under the SPM-1 model and there is failure cause misclassification. CIF is estimated at certain years since baseline. Open circles show the empirical estimates based on posterior medians over 500 replications whereas closed circles show the true values. Shown are also the corresponding empirical coverage probabilities (%). The true marker is based on linear splines with knots at 1 and 5 years since baseline and it is correctly specified when fitting SPM-1 and SPM-2 models.

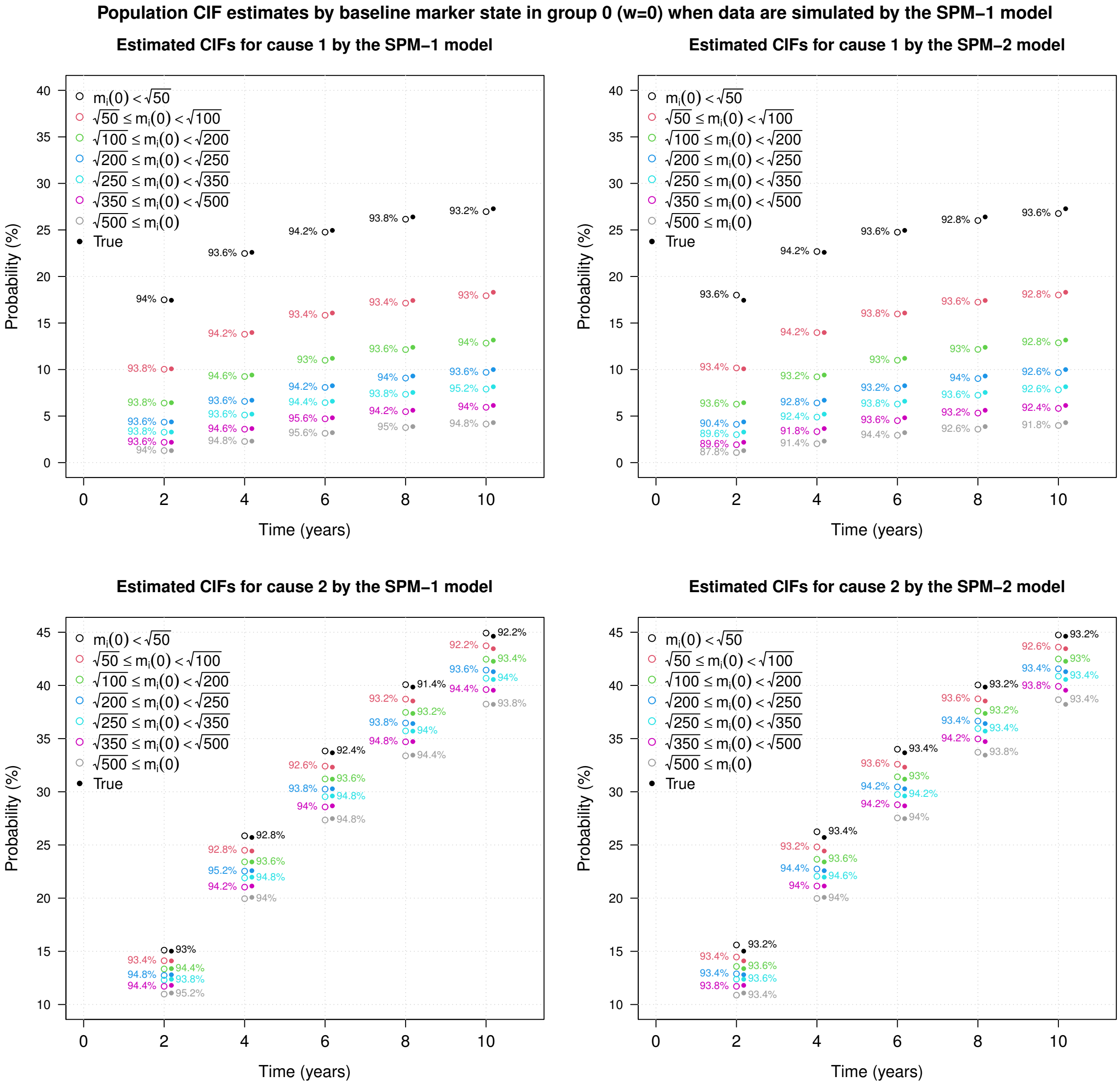

Figure S4. Simulation study results for population-averaged CIF estimates by baseline marker state for group 0 when data are simulated under the SPM-1 model and there is failure cause misclassification. CIF is estimated at certain years since baseline. Open circles show the empirical estimates based on posterior medians over 500 replications whereas closed circles show the true values. Shown are also the corresponding empirical coverage probabilities (%). The true marker is based on linear splines with knots at 1 and 5 years since baseline and it is correctly specified when fitting SPM-1 and SPM-2 models.

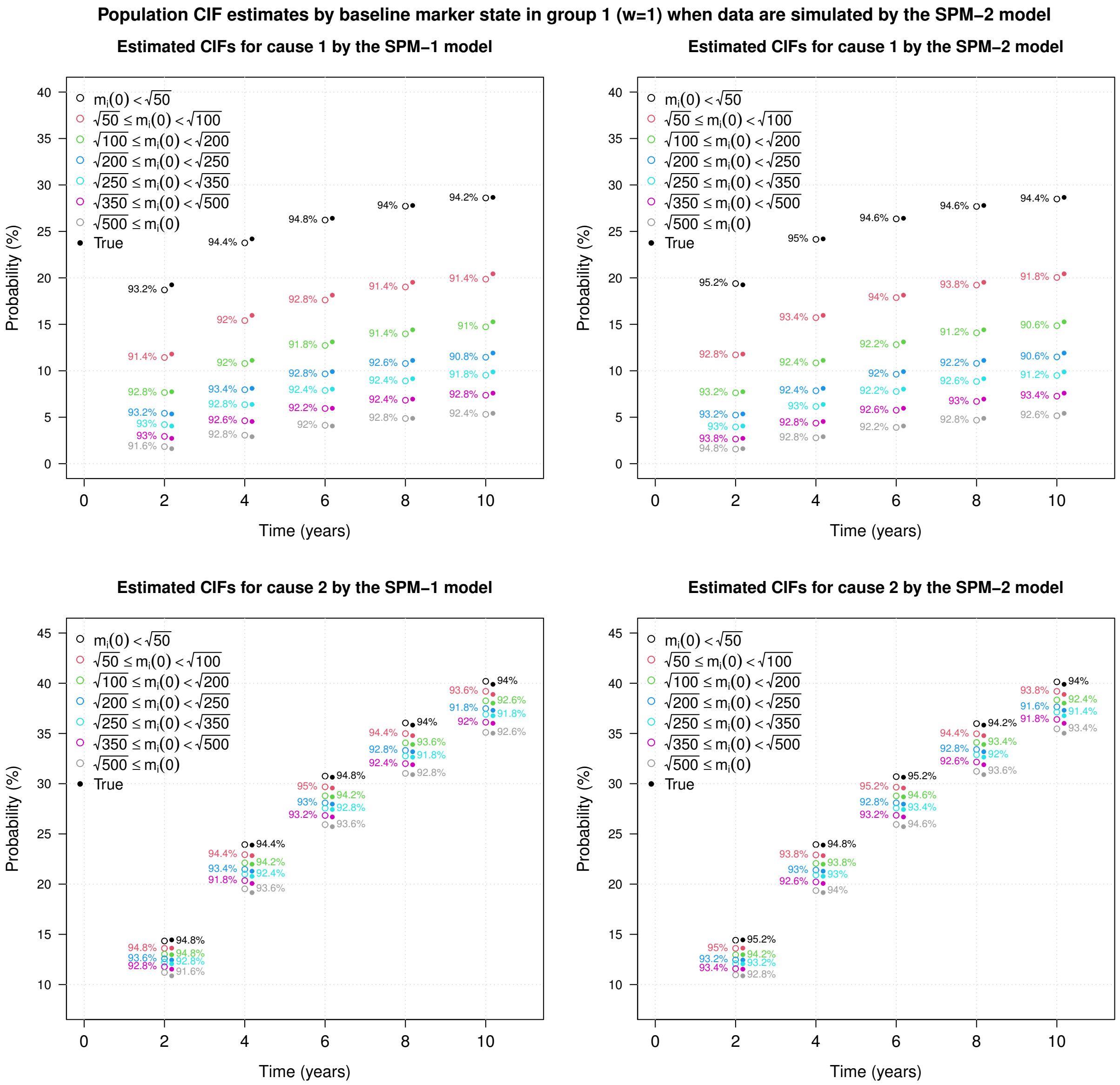

Figure S5. Simulation study results for population-averaged CIF estimates by baseline marker state for group 1 when data are simulated under the SPM-2 model and there is failure cause misclassification. CIF is estimated at certain years since baseline. Open circles show the empirical estimates based on posterior medians over 500 replications whereas closed circles show the true values. Shown are also the corresponding empirical coverage probabilities (%). The true marker is based on linear splines with knots at 1 and 5 years since baseline and it is correctly specified when fitting SPM-1 and SPM-2 models.

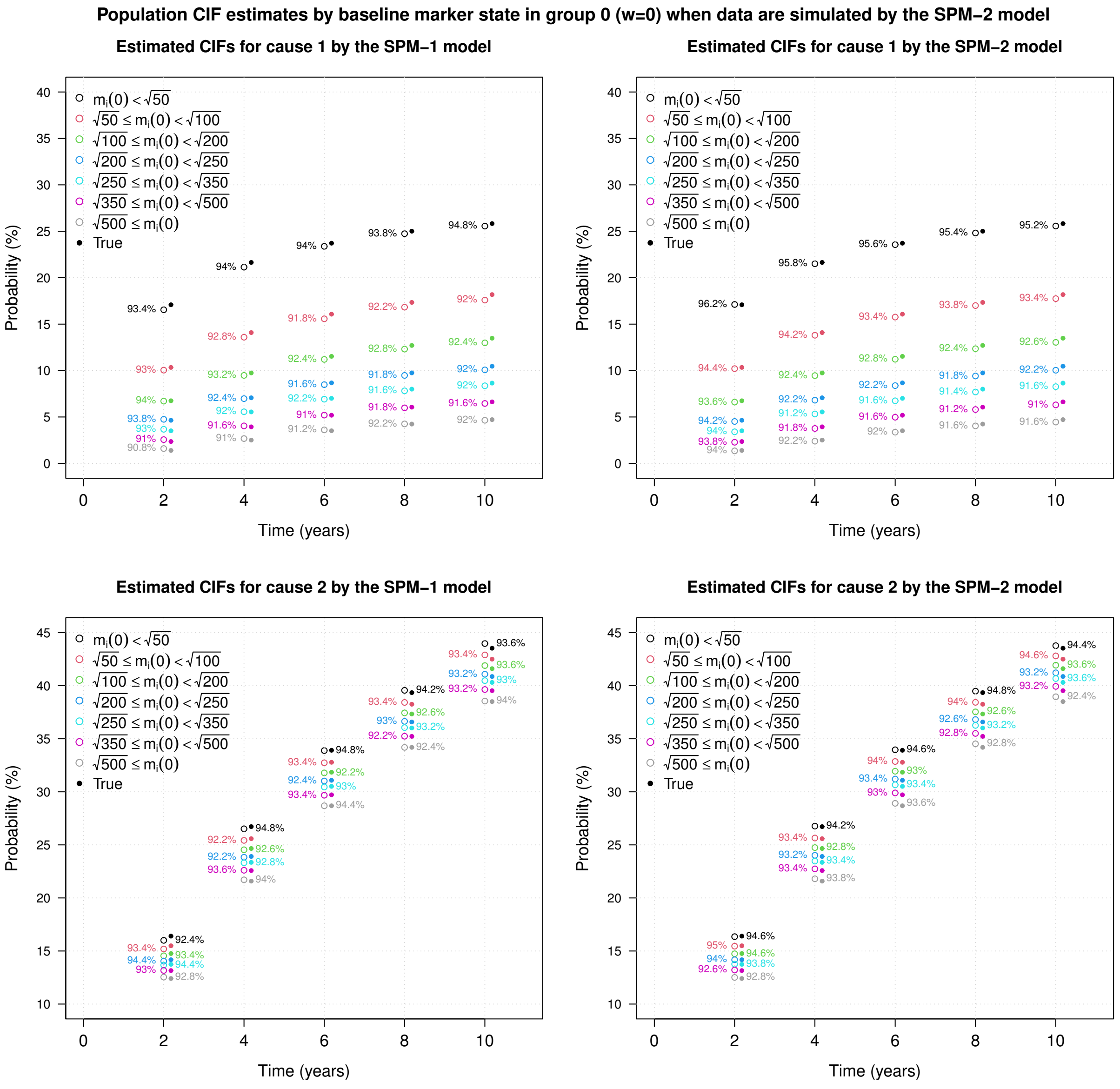

Figure S6. Simulation study results for population-averaged CIF estimates by baseline marker state for group 0 when data are simulated under the SPM-2 model and there is failure cause misclassification. CIF is estimated at certain years since baseline. Open circles show the empirical estimates based on posterior medians over 500 replications whereas closed circles show the true values. Shown are also the corresponding empirical coverage probabilities (%). The true marker is based on linear splines with knots at 1 and 5 years since baseline and it is correctly specified when fitting SPM-1 and SPM-2 models.

7.4 *Simulation study in which the fitted models ignore the double sample information*

In this subsection, we present results from an additional simulation study in which the failure cause can be misclassified, i.e. the reported failure cause can be different from the true one. However, the fitted models, i.e. the SPM-1 and SPM-2 model, assume the reported failure cause to be the true one. The results are presented in Tables S9 and S10. The main conclusion is that the estimated association parameters can be seriously biased with poor coverage rate ($\sim$24%). The fixed-effect estimates were approximately unbiased with good coverage rates, whereas the bias in the estimated population-averaged CIFs were relatively small, although the coverage rate reduced to 75% in some cases.

Table S9. Simulation study results from fitted SPM-1 and SPM-2 models when the data are simulated by the SPM-1 model and there is failure cause misclassification. Both the SPM-1 and SPM-2 models assume that the reported failure cause is the true one†.

Parameter	True	Median	Bias	ASD	MCSD	Coverage	Median	Bias	ASD	MCSD	Coverage
Longitudinal	Results from SPM-1						Results from SPM-2				
Intercept	12.850	12.857	0.007	0.126	0.122	94.400	12.857	0.007	0.126	0.122	94.800
Slope1 (β_1)	6.030	6.038	0.008	0.109	0.104	96.000	6.031	0.001	0.109	0.105	96.000
Slope2 (β_2)	0.770	0.767	-0.003	0.031	0.030	95.200	0.765	-0.005	0.031	0.030	94.800
Slope3 (β_3)	0.000	-0.002	-0.002	0.016	0.017	94.000	-0.002	-0.002	0.016	0.017	94.000
Cause1 (e.g. death)											
"True" marker value (α_1)	-0.160	-0.126	0.034	0.013	0.014	24.600	-0.138		0.014	0.016	
Binary covariate (γ_1)	0.150	0.080	-0.070	0.116	0.117	91.200	0.081		0.127	0.129	
CIF1 $t = 2$, $w = 1$	8.175	7.311	-0.864	0.693	0.678	75.000	7.317	-0.859	0.703	0.689	74.600
CIF1 $t = 4$, $w = 1$	11.533	10.705	-0.828	0.890	0.882	84.400	10.714	-0.818	0.897	0.888	83.600
CIF1 $t = 6$, $w = 1$	13.495	12.871	-0.624	1.009	1.018	89.800	12.874	-0.621	1.014	1.023	90.000
CIF1 $t = 8$, $w = 1$	14.774	14.419	-0.356	1.090	1.093	93.000	14.425	-0.349	1.090	1.097	92.800
CIF1 $t = 10$, $w = 1$	15.604	15.500	-0.103	1.149	1.140	94.400	15.504	-0.099	1.146	1.141	94.800
CIF1 $t = 2$, $w = 0$	7.106	6.780	-0.327	0.658	0.647	90.800	6.805	-0.301	0.671	0.669	89.600
CIF1 $t = 4$, $w = 0$	10.062	9.938	-0.124	0.853	0.839	94.000	9.991	-0.071	0.864	0.862	93.400
CIF1 $t = 6$, $w = 0$	11.799	11.966	0.167	0.972	0.953	94.800	12.033	0.235	0.979	0.973	94.000
CIF1 $t = 8$, $w = 0$	12.935	13.417	0.482	1.051	1.022	92.600	13.499	0.563	1.054	1.042	91.000
CIF1 $t = 10$, $w = 0$	13.673	14.431	0.758	1.106	1.060	89.400	14.520	0.847	1.109	1.074	86.800
Cause2 (e.g. disengagement)											
"True" marker value (α_2)	-0.020	-0.036	-0.016	0.008	0.009	46.000	-0.045		0.010	0.010	
Binary covariate (γ_2)	-0.150	-0.119	0.031	0.074	0.073	91.800	-0.143		0.091	0.089	
CIF2 $t = 2$, $w = 1$	11.525	12.311	0.787	0.826	0.856	80.000	12.237	0.712	0.856	0.885	85.200
CIF2 $t = 4$, $w = 1$	20.352	21.075	0.724	1.145	1.233	87.400	21.029	0.677	1.180	1.257	87.600
CIF2 $t = 6$, $w = 1$	27.328	27.848	0.520	1.347	1.463	90.200	27.859	0.531	1.366	1.463	90.200
CIF2 $t = 8$, $w = 1$	32.937	33.239	0.302	1.487	1.572	92.400	33.322	0.385	1.482	1.546	93.000
CIF2 $t = 10$, $w = 1$	37.431	37.512	0.081	1.590	1.672	93.000	37.653	0.222	1.563	1.621	92.600
CIF2 $t = 2$, $w = 0$	13.260	13.753	0.493	0.885	0.861	91.200	13.851	0.591	0.924	0.893	89.600
CIF2 $t = 4$, $w = 0$	23.228	23.392	0.164	1.202	1.172	94.800	23.490	0.262	1.237	1.214	93.600
CIF2 $t = 6$, $w = 0$	30.982	30.739	-0.243	1.390	1.396	92.600	30.799	-0.183	1.408	1.419	92.600
CIF2 $t = 8$, $w = 0$	37.130	36.528	-0.602	1.517	1.538	92.000	36.540	-0.591	1.510	1.533	91.400
CIF2 $t = 10$, $w = 0$	41.997	41.073	-0.924	1.611	1.611	90.800	41.029	-0.968	1.578	1.590	90.600

† Results from 500 replications with each dataset including 1500 individuals. The true marker evolution was based on linear splines with knots at 1 and 5 years since baseline and it was correctly specified in the fitted SPM-1 and SPM-2 models. "True" denotes the true parameter values; "Median" the mean of posterior medians over the 500 replications; "Bias" the mean bias for posterior median estimates; "ASD" the average posterior standard deviation, "MCSD" the empirical Monte carlo deviation of estimates and "Coverage" the empirical coverage probability (%) of posterior credible intervals.

Table S10. Simulation study results from fitted SPM-1 and SPM-2 models when the data are simulated by the SPM-2 model and there is failure cause misclassification. Both the SPM-1 and SPM-2 models assume that the reported failure cause is the true one†.

Parameter	True	Median	Bias	ASD	MCSD	Coverage	Median	Bias	ASD	MCSD	Coverage
Longitudinal	Results from SPM-1						Results from SPM-2				
Intercept	12.850	12.846	-0.004	0.126	0.125	95.800	12.846	-0.004	0.126	0.125	95.600
Slope1 (β_1)	6.030	6.043	0.013	0.110	0.108	95.800	6.037	0.007	0.110	0.108	95.600
Slope2 (β_2)	0.770	0.770	0.000	0.031	0.032	93.400	0.768	-0.002	0.031	0.032	93.800
Slope3 (β_3)	0.000	0.000	0.000	0.017	0.017	95.400	-0.000	-0.000	0.017	0.017	95.400
Cause1 (e.g. death)											
"True" marker value (α_1)	-0.160	-0.112		0.013	0.012		-0.124	0.036	0.014	0.014	24.800
Binary covariate (γ_1)	0.150	0.078		0.116	0.127		0.082	-0.068	0.128	0.138	91.200
CIF1 $t = 2$, $w = 1$	8.319	7.529	-0.790	0.709	0.752	78.000	7.559	-0.759	0.721	0.763	79.000
CIF1 $t = 4$, $w = 1$	11.572	10.872	-0.701	0.901	0.956	85.600	10.911	-0.662	0.913	0.962	84.400
CIF1 $t = 6$, $w = 1$	13.469	12.992	-0.476	1.017	1.088	89.400	13.027	-0.441	1.026	1.085	88.800
CIF1 $t = 8$, $w = 1$	14.709	14.473	-0.236	1.094	1.178	91.200	14.509	-0.200	1.099	1.172	91.600
CIF1 $t = 10$, $w = 1$	15.521	15.506	-0.015	1.152	1.232	90.200	15.539	0.018	1.152	1.219	91.200
CIF1 $t = 2$, $w = 0$	7.288	6.999	-0.290	0.674	0.695	89.600	7.016	-0.272	0.685	0.700	90.400
CIF1 $t = 4$, $w = 0$	10.196	10.117	-0.079	0.865	0.896	92.800	10.158	-0.038	0.876	0.900	93.000
CIF1 $t = 6$, $w = 0$	11.904	12.103	0.199	0.983	1.023	91.600	12.153	0.249	0.989	1.014	92.200
CIF1 $t = 8$, $w = 0$	13.027	13.493	0.465	1.059	1.103	90.400	13.554	0.527	1.062	1.091	90.200
CIF1 $t = 10$, $w = 0$	13.765	14.456	0.692	1.115	1.156	88.200	14.525	0.760	1.114	1.143	87.200
Cause2 (e.g. disengagement)											
"True" marker value (α_2)	-0.020	-0.030		0.008	0.008		-0.037	-0.017	0.010	0.010	60.400
Binary covariate (γ_2)	-0.150	-0.090		0.074	0.076		-0.111	0.039	0.091	0.092	93.200
CIF2 $t = 2$, $w = 1$	12.861	13.672	0.810	0.882	0.928	82.400	13.600	0.739	0.914	0.958	85.000
CIF2 $t = 4$, $w = 1$	21.826	22.561	0.735	1.193	1.208	87.800	22.509	0.684	1.224	1.234	87.800
CIF2 $t = 6$, $w = 1$	28.518	29.014	0.496	1.375	1.385	92.200	29.002	0.484	1.389	1.399	93.200
CIF2 $t = 8$, $w = 1$	33.726	34.013	0.287	1.498	1.537	93.200	34.054	0.328	1.492	1.525	94.400
CIF2 $t = 10$, $w = 1$	37.837	37.904	0.067	1.592	1.629	92.400	37.985	0.148	1.566	1.592	92.800
CIF2 $t = 2$, $w = 0$	14.636	14.859	0.223	0.926	0.986	92.400	14.950	0.314	0.966	1.027	92.000
CIF2 $t = 4$, $w = 0$	24.488	24.404	-0.084	1.232	1.266	93.200	24.492	0.003	1.267	1.301	93.600
CIF2 $t = 6$, $w = 0$	31.666	31.261	-0.404	1.404	1.457	93.000	31.323	-0.343	1.419	1.473	93.800
CIF2 $t = 8$, $w = 0$	37.150	36.531	-0.619	1.520	1.547	93.400	36.561	-0.588	1.511	1.535	92.400
CIF2 $t = 10$, $w = 0$	41.417	40.608	-0.808	1.608	1.610	91.400	40.606	-0.811	1.576	1.574	91.800

† Results from 500 replications with each dataset including 1500 individuals. The true marker evolution was based on linear splines with knots at 1 and 5 years since baseline and it was correctly specified in the fitted SPM-1 and SPM-2 models. "True" denotes the true parameter values; "Median" the mean of posterior medians over the 500 replications; "Bias" the mean bias for posterior median estimates; "ASD" the average posterior standard deviation, "MCSD" the empirical Monte carlo deviation of estimates and "Coverage" the empirical coverage probability (%) of posterior credible intervals.

7.5 *Simulation study with fully observed failure cause*

In this subsection, we present results from an additional simulation study in which there is no misclassification of events, i.e. the true failure cause always coincide with the reported one. As expected, no serious biases occurred.

Table S11. Simulation study results from fitted SPM-1 and SPM-2 models when the data are simulated by the SPM-1 model and there is no failure cause misclassification†.

Parameter	True	Median	Bias	ASD	MCSD	Coverage	Median	Bias	ASD	MCSD	Coverage
Longitudinal	Results from SPM-1						Results from SPM-2				
Intercept	12.850	12.851	0.001	0.145	0.156	92.600	12.851	0.001	0.145	0.156	93.000
Slope1 (β_1)	6.030	6.026	-0.004	0.126	0.134	92.600	6.019	-0.011	0.126	0.133	92.600
Slope2 (β_2)	0.770	0.768	-0.002	0.036	0.036	95.400	0.766	-0.004	0.036	0.036	96.200
Slope3 (β_3)	0.000	-0.001	-0.001	0.019	0.020	94.800	-0.002	-0.002	0.019	0.020	94.600
Cause1 (e.g. death)											
"True" marker value (α_1)	-0.160	-0.161	-0.001	0.014	0.014	95.800	-0.179		0.017	0.016	
Binary covariate (γ_1)	0.150	0.145	-0.005	0.132	0.137	93.400	0.157		0.148	0.153	
CIF1 $t = 2$, $w = 1$	8.175	8.093	-0.082	0.838	0.818	93.600	8.120	-0.055	0.854	0.827	95.200
CIF1 $t = 4$, $w = 1$	11.533	11.397	-0.135	1.039	1.016	95.000	11.428	-0.105	1.056	1.020	95.200
CIF1 $t = 6$, $w = 1$	13.495	13.365	-0.130	1.157	1.129	95.800	13.389	-0.106	1.171	1.126	95.600
CIF1 $t = 8$, $w = 1$	14.774	14.640	-0.134	1.230	1.187	95.400	14.661	-0.113	1.242	1.186	95.600
CIF1 $t = 10$, $w = 1$	15.604	15.455	-0.148	1.278	1.247	95.600	15.473	-0.131	1.288	1.247	95.600
CIF1 $t = 2$, $w = 0$	7.106	7.073	-0.033	0.774	0.789	93.600	7.092	-0.015	0.790	0.807	93.600
CIF1 $t = 4$, $w = 0$	10.062	9.995	-0.067	0.976	0.984	94.200	10.038	-0.024	0.992	1.000	94.800
CIF1 $t = 6$, $w = 0$	11.799	11.743	-0.056	1.094	1.097	95.000	11.799	0.001	1.108	1.109	95.600
CIF1 $t = 8$, $w = 0$	12.935	12.880	-0.056	1.167	1.166	95.000	12.948	0.013	1.179	1.172	95.200
CIF1 $t = 10$, $w = 0$	13.673	13.607	-0.067	1.216	1.212	94.200	13.684	0.010	1.226	1.219	95.400
Cause2 (e.g. disengagement)											
"True" marker value (α_2)	-0.020	-0.021	-0.001	0.009	0.009	94.600	-0.026		0.011	0.011	
Binary covariate (γ_2)	-0.150	-0.149	0.001	0.086	0.087	94.400	-0.180		0.105	0.106	
CIF2 $t = 2$, $w = 1$	11.525	11.494	-0.030	0.914	0.869	94.800	11.412	-0.113	0.950	0.909	95.200
CIF2 $t = 4$, $w = 1$	20.352	20.290	-0.062	1.294	1.290	95.000	20.249	-0.102	1.338	1.342	94.200
CIF2 $t = 6$, $w = 1$	27.328	27.258	-0.071	1.540	1.581	94.200	27.293	-0.035	1.565	1.619	93.200
CIF2 $t = 8$, $w = 1$	32.937	32.864	-0.073	1.711	1.747	94.000	32.991	0.054	1.709	1.762	93.800
CIF2 $t = 10$, $w = 1$	37.431	37.352	-0.080	1.839	1.899	92.600	37.566	0.135	1.811	1.880	92.200
CIF2 $t = 2$, $w = 0$	13.260	13.212	-0.048	1.003	1.009	93.400	13.356	0.096	1.047	1.059	94.400
CIF2 $t = 4$, $w = 0$	23.228	23.138	-0.090	1.379	1.406	93.400	23.297	0.069	1.424	1.452	94.200
CIF2 $t = 6$, $w = 0$	30.982	30.868	-0.114	1.610	1.620	95.600	30.978	-0.004	1.631	1.631	94.400
CIF2 $t = 8$, $w = 0$	37.130	37.014	-0.116	1.765	1.789	93.800	37.057	-0.073	1.756	1.765	94.400
CIF2 $t = 10$, $w = 0$	41.997	41.861	-0.136	1.876	1.902	93.800	41.834	-0.163	1.838	1.846	94.000

† Results from 500 replications with each dataset including 1500 individuals. The true marker evolution was based on linear splines with knots at 1 and 5 years since baseline and it was correctly specified in the fitted SPM-1 and SPM-2 models. "True" denotes the true parameter values; "Median" the mean of posterior medians over the 500 replications; "Bias" the mean bias for posterior median estimates; "ASD" the average posterior standard deviation, "MCSD" the empirical Monte carlo deviation of estimates and "Coverage" the empirical coverage probability (%) of posterior credible intervals.

Table S12. Simulation study results from fitted SPM-1 and SPM-2 models when the data are simulated by the SPM-2 model and there is no failure cause misclassification†.

Parameter	True	Median	Bias	ASD	MCSD	Coverage	Median	Bias	ASD	MCSD	Coverage
Longitudinal	Results from SPM-1						Results from SPM-2				
Intercept	12.850	12.845	-0.005	0.146	0.137	95.600	12.844	-0.006	0.146	0.138	95.800
Slope1 (β_1)	6.030	6.040	0.010	0.127	0.126	94.600	6.033	0.003	0.127	0.126	94.600
Slope2 (β_2)	0.770	0.772	0.002	0.036	0.034	95.600	0.770	-0.000	0.036	0.034	96.400
Slope3 (β_3)	0.000	0.001	0.001	0.019	0.020	92.600	0.001	0.001	0.019	0.020	92.600
Cause1 (e.g. death)											
"True" marker value (α_1)	-0.160	-0.143		0.014	0.014		-0.161	-0.001	0.017	0.016	96.000
Binary covariate (γ_1)	0.150	0.133		0.133	0.132		0.146	-0.004	0.149	0.148	94.200
CIF1 $t = 2$, $w = 1$	8.319	8.216	-0.103	0.853	0.841	95.000	8.270	-0.048	0.869	0.858	93.800
CIF1 $t = 4$, $w = 1$	11.572	11.464	-0.108	1.055	1.052	94.400	11.529	-0.043	1.069	1.064	93.400
CIF1 $t = 6$, $w = 1$	13.469	13.376	-0.093	1.172	1.171	95.600	13.439	-0.030	1.182	1.180	94.000
CIF1 $t = 8$, $w = 1$	14.709	14.608	-0.102	1.245	1.235	96.000	14.669	-0.040	1.251	1.244	95.200
CIF1 $t = 10$, $w = 1$	15.521	15.430	-0.091	1.296	1.288	95.400	15.489	-0.032	1.298	1.294	95.200
CIF1 $t = 2$, $w = 0$	7.288	7.258	-0.030	0.794	0.774	95.400	7.274	-0.014	0.808	0.786	95.400
CIF1 $t = 4$, $w = 0$	10.196	10.158	-0.039	0.995	0.977	94.600	10.198	0.002	1.007	0.990	94.600
CIF1 $t = 6$, $w = 0$	11.904	11.871	-0.033	1.112	1.094	93.800	11.920	0.017	1.120	1.102	94.200
CIF1 $t = 8$, $w = 0$	13.027	12.979	-0.049	1.185	1.180	94.400	13.041	0.013	1.190	1.181	94.400
CIF1 $t = 10$, $w = 0$	13.765	13.723	-0.042	1.235	1.265	93.600	13.791	0.026	1.238	1.264	93.800
Cause2 (e.g. disengagement)											
"True" marker value (α_2)	-0.020	-0.015		0.009	0.009		-0.019	0.001	0.011	0.011	95.400
Binary covariate (γ_2)	-0.150	-0.128		0.086	0.086		-0.157	-0.007	0.105	0.104	94.200
CIF2 $t = 2$, $w = 1$	12.861	12.924	0.063	0.984	0.981	94.800	12.826	-0.035	1.020	1.019	94.000
CIF2 $t = 4$, $w = 1$	21.826	21.839	0.014	1.352	1.321	94.000	21.766	-0.060	1.389	1.364	94.200
CIF2 $t = 6$, $w = 1$	28.518	28.472	-0.046	1.576	1.583	94.000	28.457	-0.061	1.593	1.600	94.200
CIF2 $t = 8$, $w = 1$	33.726	33.594	-0.132	1.728	1.781	93.000	33.647	-0.079	1.717	1.770	93.400
CIF2 $t = 10$, $w = 1$	37.837	37.586	-0.252	1.842	1.932	92.600	37.700	-0.137	1.805	1.894	92.400
CIF2 $t = 2$, $w = 0$	14.636	14.547	-0.089	1.060	1.105	93.200	14.685	0.049	1.106	1.143	93.000
CIF2 $t = 4$, $w = 0$	24.488	24.406	-0.082	1.422	1.438	93.200	24.545	0.057	1.463	1.461	94.200
CIF2 $t = 6$, $w = 0$	31.666	31.640	-0.026	1.633	1.640	94.000	31.738	0.072	1.650	1.646	94.200
CIF2 $t = 8$, $w = 0$	37.150	37.172	0.022	1.774	1.804	94.400	37.217	0.068	1.760	1.778	94.600
CIF2 $t = 10$, $w = 0$	41.417	41.438	0.022	1.877	1.908	94.400	41.427	0.011	1.834	1.854	93.800

† Results from 500 replications with each dataset including 1500 individuals. The true marker evolution was based on linear splines with knots at 1 and 5 years since baseline and it was correctly specified in the fitted SPM-1 and SPM-2 models. "True" denotes the true parameter values; "Median" the mean of posterior medians over the 500 replications; "Bias" the mean bias for posterior median estimates; "ASD" the average posterior standard deviation, "MCSD" the empirical Monte carlo deviation of estimates and "Coverage" the empirical coverage probability (%) of posterior credible intervals.

8. Additional results for the models applied to the IeDEA study data

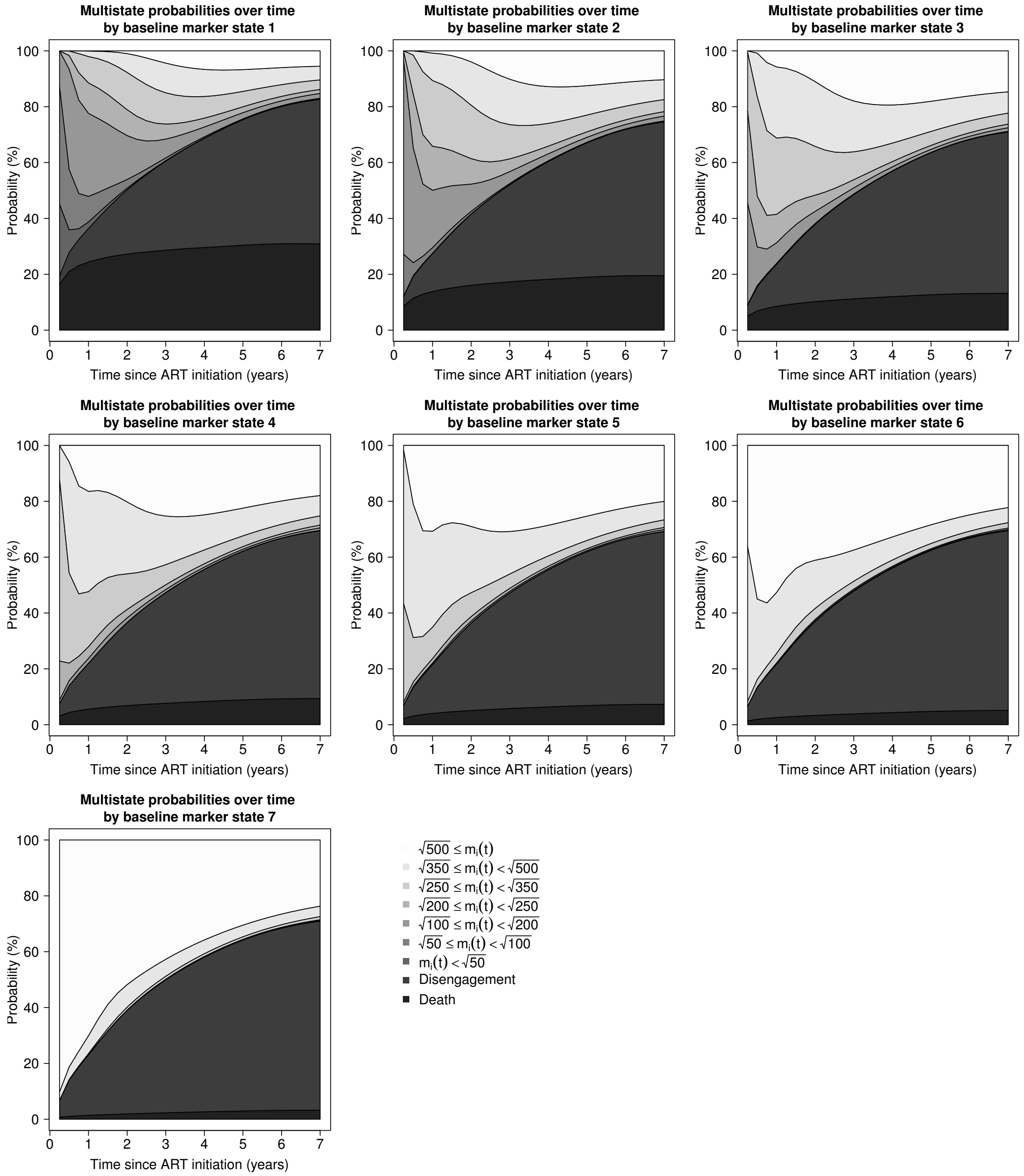

Figure S7. Transition probabilities by baseline marker state as estimated by the proposed model assuming $c_1 = 0.5$ for death and the proportional subdistribution hazard model (SPM-1) for disengagement from care, accounting for failure cause misclassification, applied to the East Africa IeDEA data. State 1 is $\{m_i(t) < \sqrt{50}\}$, State 2 is $\{\sqrt{100} \leqslant m_i(t) < \sqrt{200}\}$, State 3 is $\{\sqrt{200} \leqslant m_i(t) < \sqrt{250}\}$, State 4 is $\{\sqrt{200} \leqslant m_i(t) < \sqrt{250}\}$, State 5 is $\{\sqrt{250} \leqslant m_i(t) < \sqrt{350}\}$, State 6 is $\{\sqrt{350} \leqslant m_i(t) < \sqrt{500}\}$, State 7 is $\{\sqrt{500} \leqslant m_i(t)\}$.

In the application to the East Africa IeDEA data, the form of the prior distributions used in the simulation study, with $\boldsymbol{\mu}_0 = \boldsymbol{\mu}_0^s = \mathbf{0}$, $\boldsymbol{C}_0$ and $\boldsymbol{C}_0^s$ diagonal matrices with variances 100, $\lambda_1 = \lambda_2 = 0.01$, $df = 2$, $\boldsymbol{A} = 2 \times \text{diag}(25, 1)$, and $\pi_{11} \sim \text{Beta}(1, 1)$ (uniform distribution). To estimate model parameters, we used 1000 iterations as burn-in, recorded 50000 draws thereafter keeping every tenth posterior value for inferences.

9. Results from one simulated dataset

The following results were obtained using the code posted as supplementary material available at http://biostatistics.oxfordjournals.org [R version 4.1.2 (2021-11-01)] for the SPM-1 model:

```
set.seed(15)
# Fit the SPM-1 model (subdistribution hazard)
fitProp_Subd = try(jointModel_Subdistr_Misc(fitlme,fitCoxCause1,fitCoxCause2,
prior,startingValues = startingValues,ndraw = 3500,
thin = 3,nburn = 200,iterMaxSurv_1 = 2,iterMaxSurv_2 = 1,store.b = T,
nknotsCause1 = 2,nknotsCause2 = 3,scaleVarSurv = 1,useGauleg = T,Gauleg_points = 30),
                   silent = T)

> round(fitProp_Subd$summary$Longitudinal_Process,3)
                                 Post.mean Post.sd MC.se   2.5%    50%  97.5%
(Intercept)                         12.908   0.127 0.002 12.662 12.904 13.164
lspline(times, knots = c(1, 5))1     5.986   0.106 0.002  5.779  5.987  6.194
lspline(times, knots = c(1, 5))2     0.756   0.031 0.001  0.695  0.756  0.814
lspline(times, knots = c(1, 5))3    -0.002   0.017 0.000 -0.035 -0.002  0.030
Variance                             8.169   0.081 0.001  8.012  8.171  8.325
> round(fitProp_Subd$summary$sumSurvCause1,3)
```

```
               Post.mean Post.sd MC.se   2.5%    50%  97.5%
group              0.137   0.153 0.003 -0.161  0.134  0.439
Assoc1            -0.156   0.016 0.000 -0.189 -0.156 -0.124
GammasCause1_1    -0.751   0.302 0.005 -1.367 -0.734 -0.175
GammasCause1_2    -1.364   0.399 0.007 -2.164 -1.357 -0.610
GammasCause1_3    -0.731   0.457 0.008 -1.631 -0.723  0.126
GammasCause1_4    -1.811   0.867 0.015 -3.596 -1.808 -0.212
GammasCause1_5    -2.270   1.273 0.022 -4.768 -2.227  0.161
GammasCause1_6    -3.582   1.491 0.025 -7.667 -3.323 -1.582
> round(fitProp_Subd$summary$sumSurvCause2,3)
               Post.mean Post.sd MC.se   2.5%    50%  97.5%
group             -0.052   0.083 0.001 -0.209 -0.052  0.112
Assoc2            -0.028   0.009 0.000 -0.046 -0.028 -0.009
GammasCause2_1    -2.309   0.301 0.005 -2.919 -2.295 -1.764
GammasCause2_2    -2.103   0.306 0.005 -2.712 -2.103 -1.514
GammasCause2_3    -2.334   0.304 0.005 -2.939 -2.331 -1.734
GammasCause2_4    -2.213   0.298 0.005 -2.799 -2.210 -1.634
GammasCause2_5    -2.392   0.373 0.006 -3.130 -2.389 -1.663
GammasCause2_6    -2.410   0.395 0.007 -3.192 -2.404 -1.619
GammasCause2_7    -2.551   0.347 0.006 -3.264 -2.549 -1.872
> round(fitProp_Subd$summary$sumMiscl,3)
                   Post.mean Post.sd MC.se  2.5%   50% 97.5%
Pr(Kobs=1|Ktrue=1)     0.844   0.051 0.001 0.736 0.846 0.934
Pr(Kobs=2|Ktrue=2)     0.873   0.023 0.000 0.827 0.872 0.919
```

```
# Marginal DIC criterion

fitDICsubd = DICSubdistr_Misc(fitProp_Subd,nMC = 200,thinDIC = 7)

> fitDICsubd$dic

[1] 142315.5

> fitDICsubd$eff

[1] 28.86528


newdata1 = data.frame(group = c(1),etimes = 0,statusObs = 0)

newdata2 = data.frame(group = c(1),etimes = 0,statusObs = 0)


tt = seq(0,10,by = 2)

fit1 = predictLatentStates_Subdistr(fitProp_Subd,newdata1,newdata2,

tt = tt,nMC = 1000,thin = 10)


####################################################################

### Predict population CIFs and latent marker states for group 0 ###

####################################################################

newdata1 = data.frame(group = c(0),etimes = 0,statusObs = 0)

newdata2 = data.frame(group = c(0),etimes = 0,statusObs = 0)


fit0 = predictLatentStates_Subdistr(fitProp_Subd,newdata1,newdata2,

tt = tt,nMC = 1000,thin = 10)


> round(100*fit1$sumCif1,1)

  times Mean Median  SD   LB   UB
```

```
1     0  0.0    0.0 0.0  0.0  0.0
2   200  7.0    6.9 0.9  5.4  9.0
3   400 10.3   10.2 1.2  8.2 12.6
4   600 11.8   11.8 1.4  9.3 14.6
5   800 12.7   12.7 1.5  9.9 15.7
6  1000 13.2   13.2 1.6 10.3 16.5
> round(100*fit1$sumCif2,1)
  times Mean Median  SD   LB   UB
1     0  0.0    0.0 0.0  0.0  0.0
2   200 12.0   12.0 1.1 10.1 14.3
3   400 21.5   21.4 1.5 18.7 24.3
4   600 29.4   29.3 1.7 26.2 32.9
5   800 36.0   35.9 1.9 32.5 39.8
6  1000 41.5   41.3 2.1 37.8 45.6
> round(100*fit1$sumS1,1)
  times Mean Median  SD   LB   UB
1     0 12.8   12.8 0.7 11.5 14.4
2   200  0.2    0.2 0.0  0.1  0.3
3   400  0.0    0.0 0.0  0.0  0.1
4   600  0.0    0.0 0.0  0.0  0.0
5   800  0.0    0.0 0.0  0.0  0.0
6  1000  0.0    0.0 0.0  0.0  0.0
> round(100*fit1$sumS2,1)
  times Mean Median  SD   LB   UB
1     0 15.8   15.8 0.3 15.2 16.3
```

```
2   200  1.0    1.0 0.1  0.8  1.2
3   400  0.3    0.3 0.0  0.2  0.4
4   600  0.2    0.2 0.0  0.1  0.3
5   800  0.1    0.1 0.0  0.1  0.2
6  1000  0.1    0.1 0.0  0.0  0.1
> round(100*fit1$sumS3,1)
  times Mean Median  SD   LB   UB
1     0 30.9   30.9 0.6 29.9 32.1
2   200  7.1    7.2 0.3  6.5  7.8
3   400  3.1    3.1 0.2  2.6  3.6
4   600  2.1    2.1 0.2  1.7  2.5
5   800  1.5    1.5 0.2  1.2  1.9
6  1000  1.1    1.1 0.2  0.8  1.5
> round(100*fit1$sumS4,1)
  times Mean Median  SD   LB   UB
1     0 11.9   11.9 0.2 11.4 12.3
2   200  6.5    6.5 0.2  6.2  6.9
3   400  3.5    3.5 0.2  3.2  3.8
4   600  2.4    2.4 0.1  2.1  2.7
5   800  1.9    1.9 0.1  1.6  2.2
6  1000  1.5    1.5 0.1  1.3  1.8
> round(100*fit1$sumS5,1)
  times Mean Median  SD   LB   UB
1     0 15.6   15.6 0.3 15.0 16.3
2   200 16.8   16.8 0.4 16.1 17.5
```

```
3   400 10.8   10.8 0.3 10.1 11.5
4   600  7.7    7.6 0.3  7.1  8.3
5   800  6.4    6.4 0.3  5.8  7.0
6  1000  5.4    5.4 0.3  4.9  6.0
> round(100*fit1$sumS6,1)
  times Mean Median  SD   LB   UB
1     0  9.6    9.6 0.4  8.9 10.3
2   200 24.5   24.5 0.5 23.5 25.5
3   400 20.2   20.2 0.5 19.2 21.2
4   600 15.6   15.6 0.5 14.7 16.6
5   800 13.6   13.6 0.5 12.7 14.5
6  1000 11.9   11.9 0.5 11.0 12.9
> round(100*fit1$sumS7,1)
  times Mean Median  SD   LB   UB
1     0  3.3    3.3 0.3  2.7  3.9
2   200 24.8   24.8 0.8 23.2 26.4
3   400 30.3   30.3 0.9 28.6 32.1
4   600 30.8   30.8 1.0 28.9 32.7
5   800 27.8   27.7 1.0 25.9 29.7
6  1000 25.2   25.3 1.0 23.3 27.1
```

and for the SPM-2 model

```
set.seed(15)
# Fit the SPM-2 model
fitProp_IncrCumInc = try(jointModel_IncrCumInc_Misc(fitlme,fitCoxCause1,fitCoxCause2,
```

```
prior,startingValues = startingValues,ndraw = 3500,thin = 3,nburn = 200,
alpha1 = 1,alpha2 = 1,iterMaxSurv_1 = 2,iterMaxSurv_2 = 1,store.b = T,
nknotsCause1 = 2,nknotsCause2 = 3,scaleVarSurv = 1,useGauleg = T,Gauleg_points = 30),
silent = T)


> round(fitProp_IncrCumInc$summary$Longitudinal_Process,3)
                                Post.mean Post.sd MC.se   2.5%    50%  97.5%
(Intercept)                        12.904   0.128 0.002 12.655 12.900 13.159
lspline(times, knots = c(1, 5))1    5.976   0.107 0.002  5.771  5.977  6.189
lspline(times, knots = c(1, 5))2    0.752   0.031 0.001  0.690  0.752  0.812
lspline(times, knots = c(1, 5))3   -0.003   0.017 0.000 -0.036 -0.003  0.030
Variance                            8.170   0.081 0.001  8.012  8.172  8.327
> round(fitProp_IncrCumInc$summary$sumSurvCause1,3)
               Post.mean Post.sd MC.se   2.5%    50%  97.5%
group              0.143   0.176 0.003 -0.201  0.139  0.500
Assoc1            -0.174   0.020 0.000 -0.214 -0.173 -0.135
GammasCause1_1    -0.603   0.323 0.005 -1.255 -0.594 -0.004
GammasCause1_2    -1.114   0.423 0.007 -1.961 -1.114 -0.323
GammasCause1_3    -0.374   0.509 0.009 -1.376 -0.363  0.643
GammasCause1_4    -1.292   0.865 0.015 -3.027 -1.274  0.398
GammasCause1_5    -1.831   1.241 0.021 -4.387 -1.766  0.565
GammasCause1_6    -3.018   1.226 0.021 -5.962 -2.859 -1.094
> round(fitProp_IncrCumInc$summary$sumSurvCause2,3)
               Post.mean Post.sd MC.se   2.5%    50%  97.5%
group             -0.070   0.104 0.002 -0.270 -0.069  0.136
```

```
Assoc2            -0.037   0.012 0.000 -0.060 -0.037 -0.014
GammasCause2_1    -2.182   0.316 0.005 -2.829 -2.170 -1.615
GammasCause2_2    -1.894   0.333 0.006 -2.572 -1.888 -1.260
GammasCause2_3    -2.031   0.342 0.006 -2.682 -2.033 -1.369
GammasCause2_4    -1.791   0.340 0.006 -2.471 -1.800 -1.103
GammasCause2_5    -1.787   0.414 0.007 -2.600 -1.775 -1.003
GammasCause2_6    -1.712   0.427 0.007 -2.544 -1.704 -0.875
GammasCause2_7    -1.800   0.395 0.007 -2.597 -1.780 -1.066
> round(fitProp_IncrCumInc$summary$sumMiscl,3)
                   Post.mean Post.sd MC.se  2.5%   50% 97.5%
Pr(Kobs=1|Ktrue=1)     0.848   0.051 0.001 0.737 0.851 0.938
Pr(Kobs=2|Ktrue=2)     0.871   0.023 0.000 0.825 0.871 0.915


fitDICIncrCinc = DICIncrCumInc_Misc(fitProp_IncrCumInc,nMC = 200,thinDIC = 7)
fitDICIncrCinc$eff
fitDICIncrCinc$dic


> fitDICIncrCinc$eff
[1] 29.28249
> fitDICIncrCinc$dic
[1] 142316.1



####################################################################
### Predict population CIFs and latent marker states for group 1 ###
```

```
####################################################################
newdata1 = data.frame(group = c(1),etimes = 0,statusObs = 0)
newdata2 = data.frame(group = c(1),etimes = 0,statusObs = 0)

fit1 = predictLatentStates_IncrCumInc(fitProp_IncrCumInc,newdata1,newdata2,tt = tt,
nMC = 1000,thin = 10)

####################################################################
### Predict population CIFs and latent marker states for group 0 ###
####################################################################
newdata1 = data.frame(group = c(0),etimes = 0,statusObs = 0)
newdata2 = data.frame(group = c(0),etimes = 0,statusObs = 0)

fit0 = predictLatentStates_IncrCumInc(fitProp_IncrCumInc,newdata1,newdata2,tt = tt,
nMC = 1000,thin = 10)

> round(100*fit1$sumCif1,1)
  times Mean Median  SD   LB   UB
1     0  0.0    0.0 0.0  0.0  0.0
2   200  7.0    6.9 0.9  5.4  9.0
3   400 10.3   10.2 1.2  8.2 12.6
4   600 11.8   11.8 1.4  9.3 14.6
5   800 12.7   12.7 1.5  9.9 15.7
6  1000 13.2   13.2 1.6 10.3 16.5
> round(100*fit1$sumCif2,1)
```

```
  times Mean Median  SD   LB   UB
1     0  0.0    0.0 0.0  0.0  0.0
2   200 12.0   12.0 1.1 10.1 14.3
3   400 21.5   21.4 1.5 18.7 24.3
4   600 29.4   29.3 1.7 26.2 32.9
5   800 36.0   35.9 1.9 32.5 39.8
6  1000 41.5   41.3 2.1 37.8 45.6
> round(100*fit1$sumS1,1)
  times Mean Median  SD   LB   UB
1     0 12.8   12.8 0.7 11.5 14.4
2   200  0.2    0.2 0.0  0.1  0.3
3   400  0.0    0.0 0.0  0.0  0.1
4   600  0.0    0.0 0.0  0.0  0.0
5   800  0.0    0.0 0.0  0.0  0.0
6  1000  0.0    0.0 0.0  0.0  0.0
> round(100*fit1$sumS2,1)
  times Mean Median  SD   LB   UB
1     0 15.8   15.8 0.3 15.2 16.3
2   200  1.0    1.0 0.1  0.8  1.2
3   400  0.3    0.3 0.0  0.2  0.4
4   600  0.2    0.2 0.0  0.1  0.3
5   800  0.1    0.1 0.0  0.1  0.2
6  1000  0.1    0.1 0.0  0.0  0.1
> round(100*fit1$sumS3,1)
  times Mean Median  SD   LB   UB
```

```
1     0 30.9   30.9 0.6 29.9 32.1

2   200  7.1    7.2 0.3  6.5  7.8

3   400  3.1    3.1 0.2  2.6  3.6

4   600  2.1    2.1 0.2  1.7  2.5

5   800  1.5    1.5 0.2  1.2  1.9

6  1000  1.1    1.1 0.2  0.8  1.5

> round(100*fit1$sumS4,1)

  times Mean Median  SD   LB   UB

1     0 11.9   11.9 0.2 11.4 12.3

2   200  6.5    6.5 0.2  6.2  6.9

3   400  3.5    3.5 0.2  3.2  3.8

4   600  2.4    2.4 0.1  2.1  2.7

5   800  1.9    1.9 0.1  1.6  2.2

6  1000  1.5    1.5 0.1  1.3  1.8

> round(100*fit1$sumS5,1)

  times Mean Median  SD   LB   UB

1     0 15.6   15.6 0.3 15.0 16.3

2   200 16.8   16.8 0.4 16.1 17.5

3   400 10.8   10.8 0.3 10.1 11.5

4   600  7.7    7.6 0.3  7.1  8.3

5   800  6.4    6.4 0.3  5.8  7.0

6  1000  5.4    5.4 0.3  4.9  6.0

> round(100*fit1$sumS6,1)

  times Mean Median  SD   LB   UB

1     0  9.6    9.6 0.4  8.9 10.3
```

```
2    200 24.5   24.5 0.5 23.5 25.5
3    400 20.2   20.2 0.5 19.2 21.2
4    600 15.6   15.6 0.5 14.7 16.6
5    800 13.6   13.6 0.5 12.7 14.5
6   1000 11.9   11.9 0.5 11.0 12.9
> round(100*fit1$sumS7,1)
  times Mean Median  SD   LB   UB
1     0  3.3    3.3 0.3  2.7  3.9
2   200 24.8   24.8 0.8 23.2 26.4
3   400 30.3   30.3 0.9 28.6 32.1
4   600 30.8   30.8 1.0 28.9 32.7
5   800 27.8   27.7 1.0 25.9 29.7
6  1000 25.2   25.3 1.0 23.3 27.1
```

References

Beyersmann, Jan, Allignol, Arthur and Schumacher, Martin. (2011). *Competing risks and multistate models with R*. Springer Science & Business Media.

Gamerman, Dani. (1997). Sampling from the posterior distribution in generalized linear mixed models. *Statistics and Computing* **7**, 57–68.

Quintero, Adrian and Lesaffre, Emmanuel. (2018). Comparing hierarchical models via the marginalized deviance information criterion. *Statistics in Medicine* **37**(16), 2440–2454.

Rizopoulos, D. (2012). *Joint Models for Longitudinal and Time-to-Event Data: With Applications in R*, Chapman & Hall/CRC Biostatistics Series. CRC Press.

Spiegelhalter, David J., Best, Nicola G., Carlin, Bradley P. and Van Der Linde, Angelika. (2002). Bayesian measures of model complexity and fit. *Journal of the Royal Statistical Society: Series B (Statistical Methodology)* **64**(4), 583–639.

Thisted, Ronald A. (1988). *Elements of statistical computing: Numerical computation*, Volume 1. CRC Press.

[*Received August 1, 2010; revised October 1, 2010; accepted for publication November 1, 2010*]
